# Quality of measurement properties of medication adherence instruments in cardiovascular diseases and type 2 diabetes mellitus: a systematic review and meta-analysis

**DOI:** 10.1186/s13643-023-02340-z

**Published:** 2023-11-22

**Authors:** Henrique Ceretta Oliveira, Daisuke Hayashi, Samantha Dalbosco Lins Carvalho, Rita de Cássia Lopes de Barros, Mayza Luzia dos Santos Neves, Carla Renata Silva Andrechuk, Neusa Maria Costa Alexandre, Paula Aver Bretanha Ribeiro, Roberta Cunha Matheus Rodrigues

**Affiliations:** 1 CEPSchool of Nursing - University of Campinas (Unicamp), 126 Tessália Vieira de Camargo Street, Campinas, São Paulo, 13083-887 Brazil; 2 Research Centre of the Montreal University Hospital (CRCHUM), 850 Rue Saint-Denis, Montréal, Québec H2X 0A9 Canada

**Keywords:** Medication adherence, Cardiovascular diseases, Diabetes mellitus, Type 2, Patient-reported outcome measures, Reproducibility of results, Psychometrics

## Abstract

**Background:**

Medication adherence has a major impact on reducing mortality and healthcare costs related to the treatment of cardiovascular diseases and diabetes mellitus. Selecting the best patient-reported outcome measure (PROM) among the many available for this kind of patient is extremely important. This study aims to critically assess, compare and synthesize the quality of the measurement properties of patient-reported outcome measures to assess medication adherence among patients with cardiovascular diseases and/or type 2 diabetes mellitus.

**Methods:**

This review followed the COnsensus-based Standards for the selection of health Measurement INstruments (COSMIN) guidelines and was reported according to the Preferred Reporting Items for Systematic Review and Meta-Analyses (PRISMA). The searches were performed in Web of Science, SCOPUS, PubMed, CINAHL, EMBASE, LILACS, PsycINFO, and ProQuest (gray literature).

**Results:**

A total of 110 records encompassing 27 different PROMs were included in the review. The included records were published between 1986 and 2023, most of which reported studies conducted in the United States and were published in English. None of the PROMs were classified in the category “a”, thus being recommended for use due to the quality of its measurement properties. The PROMs that should not be recommended for use (category “c”) are the MTA, GMAS, DMAS-7, MALMAS, ARMS-D, and 5-item questionnaire. The remaining PROMs, e.g., MMAS-8, SMAQ, MEDS, MNPS, ARMS-12, MGT, MTA-OA, MTA-Insulin, LMAS-14, MARS-5, A-14, ARMS-10, IADMAS, MAQ, MMAS-5, ProMAS, ARMS‐7, 3-item questionnaire, AS, 12-item questionnaire, and Mascard were considered as having the potential to be recommended for use (category “b”).

**Conclusion:**

None of the included PROMs met the criteria for being classified as trusted and recommended for use for patients with cardiovascular diseases and/or type 2 diabetes mellitus. However, 21 PROMs have the potential to be recommended for use, but further studies are needed to ensure their quality based on the COSMIN guideline for systematic reviews of PROMs.

**Systematic review registration:**

PROSPERO CRD42019129109

**Supplementary Information:**

The online version contains supplementary material available at 10.1186/s13643-023-02340-z.

## Background

Medication adherence has a major impact on reducing mortality and healthcare costs related to the treatment of noncommunicable diseases (NCDs), especially cardiovascular diseases (CVDs) and diabetes mellitus [1–3].

Data from 2019 by the World Health Organization (WHO) show that 7 of the top 10 causes of death in the world are noncommunicable diseases (NCDs) [4]. Ischemic heart disease is the leading cause of death and the top 10 causes of death also include stroke, hypertensive heart disease, and diabetes mellitus [5]. The United Nations General Assembly established the reduction of premature mortality from NCDs by one-third as a target for 2030 [6].

Since many patients do not adhere to treatment as prescribed [7, 8] it is paramount to properly measure medication adherence and to take actions that increase patient’s adherence. Medication adherence involves a complex set of behaviors that are influenced by a number of psychosocial determinants such as motivation, self-efficacy, beliefs, and perceived barriers, which makes its measurement particularly challenging [9].

One of the most practical and low-cost ways to assess medication adherence is through the use of measures of patient-reported outcomes (PROs), i.e., any aspect of a patient's health status that is directly assessed by the patient, without interpretation of their response by anyone other than themselves [10]. Patient-Reported Outcome Measures (PROMs) range from simple single-item measures of omitted medication doses to multi-item instruments that aggregate reasons for non-adherence.

The task of selecting the best PROM among the many available for measuring medication adherence in patients with CVDs or type 2 diabetes mellitus (T2DM) [11, 12] requires taking into consideration its conceptual structure and measurement properties.

The COnsensus-based Standards for the selection of health Measurement INstruments (COSMIN) initiative has recently published a guideline for conducting systematic reviews of studies evaluating the measurement properties of PROMs [13]. This guideline proposes the criteria to assess the methodological quality of studies on measurement properties and the quality of the self-reported measurement itself.

There are systematic reviews evaluating the quality of the measurement properties of medication adherence PROMs in patients with diabetes mellitus using the COSMIN checklist [14–16]. However, in these systematic reviews, primary studies using PROMS to measure factors related to medication non-adherence, such as beliefs, self-efficacy, satisfaction, among others, were included. To our knowledge, no systematic review has been conducted according to the COSMIN guidelines to evaluate the quality of the measurement properties of PROMs that exclusively measure medication adherence in patients with CVDs and/or T2DM.

Therefore, this systematic review aims to critically assess, compare, and synthesize the quality of the measurement properties of PROMs for medication adherence among patients with CVDs and/or T2DM.

## Methods

### Protocol development

This systematic review was reported in accordance with the Preferred Reporting Items for Systematic Review and Meta-Analyses (PRISMA) [17] (checklist available in Additional file 1) and the COSMIN guidelines for systematic reviews on PROMs [13]. The protocol of this review was registered in the International Prospective Register of Systematic Reviews (PROSPERO CRD42019129109) and published elsewhere [18].

### Eligibility criteria

Inclusion criteria:

- Studies that aimed to develop or culturally adapt a PROM to measure medication adherence among patients aged 18 or older with a CVD and/or T2DM, regardless of the language and date of publication;
- Studies reporting the assessment of one or more properties of the PROMs.

Exclusion criteria:

- Studies in which a PROM was used to measure an outcome (e.g., randomized clinical trials);
- Studies in which a PROM was used to validate another measure;
- Studies that evaluated the measurement properties of PROMs that aimed to evaluate factors related to medication nonadherence (self-efficacy, beliefs, intention, etc.);
- Study that did not provide minimally sufficient data on the results of the investigated measurement properties, even after contacting the authors.

### Sources and search strategy

The electronic literature searches were performed in July 2020 in the following databases without time limits: Web of Science, Scopus, PubMed (including Medline), CINAHL, EMBASE, LILACS, PsycINFO, and ProQuest (gray literature). Manual searches were performed in the reference lists of the articles in order to complement the main literature. An update of the searches was performed in May 2023 considering the period from 2020 to 2023. The search strategy was based on the second version of the search filter for measurement properties proposed by the COSMIN initiative [19] and also included keywords and MeSH terms related to CVDs, T2DM, PROMs, medication adherence, and measurement properties. The search strategy used in each database was created with the support of an experienced librarian and can be found in Additional file 2. The online software Rayyan QCRI was used for reference management which included the exclusion of duplicates and the evaluation of titles and abstracts [20].

### Study selection

The study selection was reported according to the PRISMA flow diagram model [17]. The evaluation of titles and abstracts after the exclusion of duplicates was done independently by three pairs of reviewers (HCO, DH, SDLC, RCLB, MLSN, and CRSA) following a practice set of 50 titles and abstracts to improve inter-reviewer agreement. Inter-reviewer agreement ranged from 96 to 98%, with an overall agreement rate of 94%. Two reviewers independently appraised full-texts for inclusion (HCO and DH). Disagreements were discussed until a consensus was reached. Lastly, the list of references of the included studies was examined to identify other studies that had not been previously identified.

### Data extraction

Data were independently extracted by two reviewers (HCO and RCMR) using an adapted version of the extraction form available in the COSMIN manual for systematic reviews of PROMs [21] which included additional fields for other relevant information. The form contains information about the study design, sample size, participants’ demographic and clinical characteristics (gender, age, disease, disease duration, and number of medications in use), response rate, presence of conflicts of interest, funding, setting, country, and language, PROMs’ number of items and domains, mode of administration, recall period, response options, range of scores, original language, available translations, number of studies evaluating the PROM, measurement properties (PROM development, content validity, structural validity, internal consistency, cross-cultural validity/measurement invariance, reliability, measurement error, criterion validity, hypothesis testing for construct validity and responsiveness), interpretability and feasibility and information to assess the studies’ methodological quality.

### Methodological quality of the studies: assessment of risk of bias

The methodological quality of the studies was assessed independently by two reviewers (HCO and RCMR) using the COSMIN Risk of Bias checklist for systematic reviews of PROMs [22, 23]. This checklist comprises items that assess the methodological quality of studies that evaluate the measurement properties of PROMs. Disagreements were discussed until a consensus was obtained and a third reviewer (NMCA) was consulted when the reviewers were among the authors of the evaluated paper. According to the COSMIN Risk of Bias checklist, items can be rated as 'very good', 'adequate', 'doubtful', 'inadequate', or 'not applicable' (NA), and each measurement property receives an overall rating based on the worst scored item [21, 23].

### Quality of the measurement properties

For each PROM, the quality of each measurement property reported by the included studies was assessed.

These results were assessed independently by two reviewers (HCO and RCMR) based on the quality criteria for good measurement properties proposed by COSMIN [21, 23]. Disagreements were discussed until a consensus was obtained. A third reviewer (NMCA) was consulted when the reviewers were among the authors of the evaluated paper. The quality of the measurement properties of each assessed PROM was classified as sufficient ( +), insufficient (-), inconsistent ( ±), or indeterminate (?), according to the proposed criteria [13, 21–23] (Table 1).

**Table 1:** Criteria for good measurement properties

Measurement property	Rating	Criteria
Structural validity	+	**CTT:** CFA: CFI or TLI or comparable measure > 0.95 OR RMSEA < 0.06 OR SRMR < 0.08 **IRT/Rasch:** No violation of unidimensionality: CFI or TLI or comparable measure > 0.95 OR RMSEA < 0.06 OR SRMR < 0.08 AND no violation of local independence: residual correlations among the items after controlling for the dominant factor < 0.20 OR Q3's < 0.37 AND no violation of monotonicity: adequate looking graphs OR item scalability > 0.30 AND adequate model fit IRT: χ2 > 0.001 Rasch: infit and outfit mean squares ≥ 0.5 and ≤ 1.5 OR Z-standardized values > − 2 and < 2
	?	CTT: not all information for ' + ' reported IRT/Rasch: model fit not reported
	-	Criteria for ' + ' not met
Internal consistency	+	At least low evidence for sufficient structural validity AND Cronbach's alpha(s) ≥ 0.70 for each unidimensional scale or subscale
	?	Criteria for “At least low evidence for sufficient structural validity” not met
	-	At least low evidence for sufficient structural validity AND Cronbach's alpha(s) < 0.70 for each unidimensional scale or subscale
Reliability	+	ICC or weighted Kappa ≥ 0.70
	?	ICC or weighted Kappa not reported
	-	ICC or weighted Kappa < 0.70
Measurement error	+	SDC or LoA < MIC
	?	MIC not defined
	-	SDC or LoA > MIC
Hypotheses testing for construct validity	+	The result is in accordance with the hypothesis
	?	No hypothesis defined (by the review team)
	-	The result is not in accordance with the hypothesis
Cross-cultural validity\measurement invariance	+	No important differences found between group factors (such as age, gender, language) in multiple group factor analysis OR no important DIF for group factors (McFadden's R^2^ < 0.02)
	?	No multiple group factor analysis OR DIF analysis performed
	-	Important differences between group factors OR DIF was found
Criterion validity	+	Correlation with gold standard ≥ 0.70 OR AUC ≥ 0.70
	?	Not all information for ' + ' reported
	-	Correlation with gold standard < 0.70 OR AUC < 0.70
Responsiveness	+	The result is in accordance with the hypothesis OR AUC ≥ 0.70
	?	No hypothesis defined (by the review team)
	-	The result is not in accordance with the hypothesis OR AUC < 0.70

Source: Extracted from Prinsen et al., 2018 [13], p. 1152

*AUC* area under the curve, *CFA* confirmatory factor analysis, *CFI* comparative fit index, *CTT* classical test theory, *DIF* differential item functioning, *ICC* intraclass correlation coefficient, *IRT* item response theory, *LoA* limits of agreement, *MIC* minimal important change, *RMSEA* root mean square error of approximation, *SEM* standard error of measurement, *SDC* smallest detectable change, *SRMR* standardized root mean residuals, *TLI* Tucker–Lewis index; “ + ” = sufficient; “ − ” = insufficient; “?” = indeterminate

When evaluating the exploratory factor analysis (EFA) in structural validity, we established a different criterion from what was previously defined by the COSMIN team [24]. The COSMIN criteria consider a sufficient result in an EFA when the first factor accounts for at least 20% of the variability and the ratio of the variance explained by the first to the second factor is greater than four [24]. Since most PROMs included in our systematic review are one-dimensional, we considered a sufficient result when the total variance explained was at least 60% and when factor loadings were equal to or greater than 0.30 [25]. In addition to the structural features of PROMS, our decision also considered the recommendation of the COSMIN manual that new criteria may be proposed by reviewers if those established by the COSMIN team do not fully meet the evaluation of one or more properties of the measure [21].

When assessing the criterion validity, it was considered that the statistical results obtained in the assessment of the relationship between the PROM and the direct objective measures would be treated as a criterion validity result. Additionally, the statistical results obtained when evaluating the relationship between the PROMs and the direct objective measures of glycosylated hemoglobin and glycaemia (used to assess metabolic control in DM) and the measures of systolic and diastolic blood pressure (used for the control of blood pressure in hypertension) were treated as a result of criterion validity, regardless of how the authors named such validity in the primary studies.

Also in the criterion validity evaluation, when dichotomous variables are evaluated, it is recommended to apply sensitivity and specificity measures, according to the risk of bias checklist, to evaluate these results. However, the guideline does not establish the reference values for the sensitivity and specificity measures for the attribution of the quality of the results. For this reason, the sensitivity and specificity results observed in the primary studies were not considered for assigning the quality ratings of the criterion validity results.

### Data synthesis

Meta-analysis was performed to pool the results of internal consistency of the PROMs, estimated by Cronbach's alpha coefficient [26]. The analysis was performed considering a random effects model and a significance level of 5%. At first, the Cronbach's alphas values of each study were transformed to Fisher's ɀ values according to the following equation [26]:





The next step was to calculate the average of ɀ weighting according to the sample size of the studies (n_j_) [26]:

The final step was the conversion of the average weighted ɀ to the estimated value of the pooled Cronbach's alpha coefficient [26]:





These calculations were performed in the software SPSS 23 using an SPSS Meta-Analysis Macro [27]. The heterogeneity was evaluated using the Chi-squared test and the I^2^ coefficient.

### Quality of evidence: adapted Grading of Recommendations Assessment, Development, and Evaluation (GRADE) by COSMIN

The overall quality of evidence of all studies was assessed using an adapted version of the Grading of Recommendations Assessment, Development, and Evaluation (GRADE) proposed by the COSMIN initiative [13]. The quality of the evidence was classified as high, moderate, low, or very low based on the following factors: risk of bias, inconsistency (of the results of the studies), imprecision (related to the sample size of the studies), and indirect results (the evidence comes from the studies that were performed in a population or context other than the ones defined in the review) [21].

The risk of bias could be classified as serious, very serious, or extremely serious, resulting in the downgrade of 1 to 3 levels, respectively (Table 2) [21].

**Table 2:** Instructions on downgrading for Risk of Bias

Risk of bias	Downgrading for Risk of Bias
No	There are multiple studies of at least adequate quality, or there is one study of very good quality available
Serious	There are multiple studies of doubtful quality available, or there is only one study of adequate quality
Very serious	There are multiple studies of inadequate quality, or there is only one study of doubtful quality available
Extremely serious	There is only one study of inadequate quality available

Source: Extracted from Mokkink et al., 2018 [21], p. 34

Regarding the inconsistency, when the reviewers could not find an explanation for inconsistent results observed across the studies, these results are considered inconsistent. Consequently, the quality of evidence is not applicable. Concerning the imprecision, when the total sample size of the summarized studies is lower than 100 (serious), one level must be downgraded and when it is lower than 50 (very serious) two levels must be downgraded. For indirectness, the reviewers can downgrade the level of evidence by one or two levels.

Thus, according to the GRADE approach, it was initially assumed that the summarized results were of high quality and, subsequently, downgraded by one or two levels per factor, considering the following aspects: risk of bias, inconsistency, imprecision, or indirect results. When the evidence was based on only one inadequate study (extremely serious risk of bias) quality of evidence was downgraded by three levels [21].

The results were assessed independently by two reviewers (HCO and RCMR). Disagreements were discussed until a consensus was obtained and a third reviewer (NMCA) was consulted when the reviewers were among the authors of the evaluated paper.

### Recommendations for selecting a PROM

The review's final stage was the establishment of recommendations to select the most appropriate PROM. PROMs were classified into three categories:

- PROMs that presented sufficient content validity and at least low quality of evidence for sufficient internal consistency;
- PROMs that are not classified in categories (a) or (c);
- PROMs that presented high-quality evidence for an insufficient measurement property.

A PROM that falls under category (a) means it is reliable and can be recommended. A PROM that falls under category (b) means it has the potential to be recommended, though further studies are needed to ensure its quality. A PROM classified under category (c) should not be recommended.

## Results

### Study selection and data extraction

**Fig. 1:**
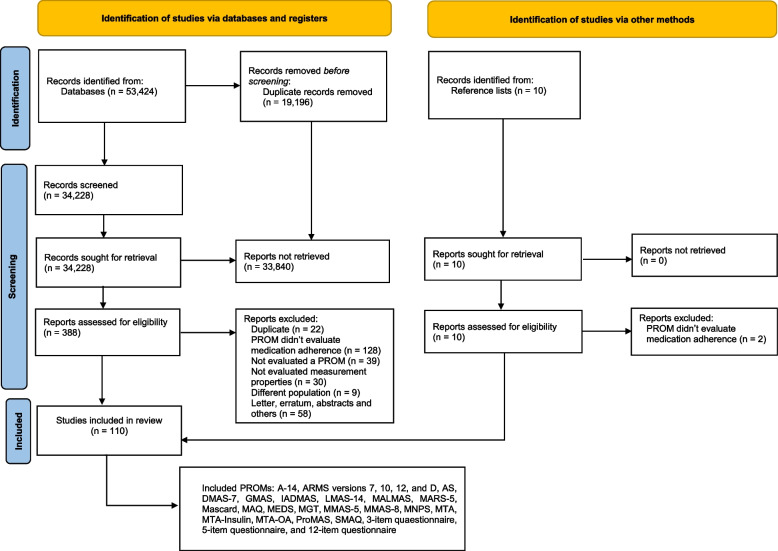
PRISMA flow diagram. Note: ARMS = Adherence to Refills and Medication Scale; AS = Adherence Scale; DMAS-7 = 7-item Diabetes Medication Adherence Scale; GMAS = General Medication Adherence Scale; IADMAS = Iraqi Anti-Diabetic Medication Adherence Scale; LMAS-14 = Fourteen-item Lebanese Medication Adherence Scale; MALMAS = Malaysian Medication Adherence Scale; MAQ = Medication Adherence Questionnaire; MARS-5 = 5-item Medication Adherence Report Scale; Mascard = Medication Adherence Scale in Cardiovascular disorders; MEDS = Medication Adherence Estimation and Differentiation Scale; MGT = Morisky-Green test; MMAS-5 = 5-item adapted Morisky Medication Adherence Scale; MMAS-8 = 8-item Morisky Medication Adherence Scale; MNPS = Medication Non-persistence Scale; MTA = Measurement of Treatment Adherence; MTA-Insulin = Measurement of Treatment Adherence—Insulin; MTA-OA = Measurement of Treatment Adherence—Oral Antidiabetics; PROM = Patient-reported outcome measures; ProMAS = Probabilistic Medication Adherence Scale; SMAQ = Simplified Medication Adherence Questionnaire

The results of the selection and data extraction of the studies are presented in the PRISMA flow diagram (Fig. 1). The searches done in July 2020 resulted in a total of 41,886 records published between 1973 and June of 2020 were considered potentially eligible and retrieved from eight databases. A total of 14,826 duplicates were removed. The titles and abstracts of 27,060 records were peer-reviewed by three pairs of peer reviewers, who evaluated 9,020 records each. A total of 336 records were identified for full-text assessment and 84 records were included. Eight additional relevant records were added after manually searching the lists of references from the included studies.

The update done in May 2023 resulted in 11,538 records published between 2020 and 2023 where 4,370 duplicates were removed. Out of 52 records assessed for full-text, 18 records were included resulting in a total of 110 records and 27 PROMs included in the systematic review (Fig. 1).

### Study and PROMs characteristics

The included studies were published between 1986 and 2023, and most of them were conducted in the United States in the English language (*n* = 19). The sample size of included studies ranged from 30 to 6,261 participants. The percentage of females ranged from 17.0% to 79.3% and the mean age ranged from 43.1 to 81.9 years. About half of the studies were conducted in hospital settings (*n* = 54) and observed an average disease duration of 9.8 years (*n* = 26) and an average of 4.5 medications in use (*n* = 29).

Most of the 27 PROMs included in the review are one-dimensional and composed of items with a Likert-type scale response. A total of 39 studies [28–66] conducted with patients who only had TD2M, another 47 studies [67–113] with patients who only had CVD, and the remaining 24 studies with patients having both T2DM and/or CVD [114–137]. The most prevalent original language of the 27 PROMs included in this review was English (*n* = 15). In addition to the original versions, translated versions of the PROMs were also included in the review. Of 27 PROMs included, 10 had been translated into at least another language. Regarding the application characteristics, for the majority of PROMs (*n* = 20) it was not clear what the recall period was (Table 3). It was not possible to describe the time to complete the PROMs because the majority of the studies did not present this information.

**Table 3:** PROMs’ characteristics

PROM	Target population	Recall period	(Sub)scale (s) (number of items)	Response options	Range of scores/scoring	Original language	Available translations
MMAS-8	DM and CVD	1 month	1 (8)	Dichotomous and Likert scale	0 to 8	English	17 languages
SMAQ	DM	NC	1 (6)	Dichotomous	6 to 12	English	2 languages
MEDS	DM and CVD	6 months	5 (16)	Likert scale	16 to 80	English	None
MNPS	DM and CVD	1 year	1 (9)	Dichotomous	0 to 9	English	None
DMAS-7	DM	NC	3 (7)	Dichotomous	0 to 7	Arabic	None
ARMS-12	DM and CVD	NC	2 (12)	Likert scale	12 to 48	English	5 languages
MGT	DM and CVD	NC	1 (4)	Dichotomous	0 to 4	English	7 languages
MTA-OA	DM	NC	1 (7)	Likert scale	1 to 6	Portuguese	None
MTA-Insulin	DM	NC	1 (7)	Likert scale	1 to 6	Portuguese	None
LMAS-14	CVD	NC	4 (14)	Likert scale	0 to 42	Arabic	None
MTA	DM and CVD	NC	1 (7)	Likert scale	1 to 6	Portuguese	None
MARS-5	DM and CVD	NC	1 (5)	Likert scale	5 to 25	English	3 languages
A-14	CVD	NC	5 (14)	Likert scale	0 to 56	German	2 languages
ARMS-10	CVD	NC	2 (10)	Likert scale	10 to 40	English	1 language
MALMAS	DM	1 month	1 (8)	Dichotomous and Likert scale	0 to 8	English	1 language
ARMS-D	DM	NC	2 (11)	Likert scale	11 to 44	English	None
IADMAS	DM	NC	1 (8)	Dichotomous and Likert scale	0 to 8	Arabic	None
GMAS	DM and CVD	NC	3 (11)	Likert scale	0 to 33	Urdu	4 languages
MAQ	CVD	NC	NC	NC	NC	Kannada, Malayalam	None
MMAS-5	CVD	1 month	1 (5)	Likert scale	No score	English	None
ProMAS	DM and CVD	NC	1 (18)	Dichotomous	0 to 18	Dutch	None
ARMS‐7	DM and CVD	NC	2 (7)	Likert scale	7 to 28	English	1 language
5-item questionnaire	CVD	1 month	1 (5)	Dichotomous	0 to 1	English	None
3-item questionnaire	DM and CVD	1 month	1 (3)	6-point Likert scale and continuous	0 to 100	English	None
AS	CVD	NC	1 (3)	Dichotomous and Likert	0 to 4	Russian	None
12-item questionnaire	DM	NC	1 (12)	Dichotomous	NC	English	None
Mascard	CVD	NC	1 (5)	Dichotomous	0 to 5	French	None

*ARMS* Adherence to Refills and Medication Scale, *AS* Adherence Scale, *CVD* Cardiovascular diseases, *DM* Diabetes mellitus, *DMAS-7* 7-item Diabetes Medication Adherence Scale, *GMAS* General Medication Adherence Scale, *IADMAS* Iraqi Anti-Diabetic Medication Adherence Scale, *LMAS-14* Fourteen-item Lebanese Medication Adherence Scale, *MALMAS* Malaysian Medication Adherence Scale, *MAQ* Medication Adherence Questionnaire, *MARS-5* 5-item Medication Adherence Report Scale, *Mascard* Medication Adherence Scale in Cardiovascular disorders, *MEDS* Medication Adherence Estimation and Differentiation Scale, *MGT* Morisky-Green test, *MMAS-5* 5-item adapted Morisky Medication Adherence Scale, *MMAS-8* 8-item Morisky Medication Adherence Scale, *MNPS* Medication Non-persistence Scale, *MTA* Measurement of Treatment Adherence, *MTA-Insulin* Measurement of Treatment Adherence – Insulin, *MTA-OA* MTA-Oral Antidiabetics, *NC* Not clear, *PROM* Patient-reported outcome measures, *ProMAS* Probabilistic Medication Adherence Scale, *SMAQ* Simplified Medication Adherence Questionnaire

A total of 38 studies reported a response rate for PROMs that ranged from 21.1% to 100.0%. Out of the 110 records, only 63.6% of the studies presented information about conflict of interests, and 68.2% informed if the research had any source of funding (Additional file 3).

The two reviewers (HCO and RCMR) are authors of one of the included studies [120]. The analysis of this article was done by a third reviewer (NMCA).

### Evidence synthesis

The summary of findings of measurement properties of the PROMs is presented in Table 4. The summary of the assessment of risk of bias can be found in the Additional files 4 and 5. The results based on each measurement property of the PROMs are presented below.

**Table 4:** Summary of findings

Measurement property	PROM	Summary	Overall rating	Quality of evidence
Content validity	MMAS-8 [28, 41, 47, 57, 61, 67, 68, 73, 76, 86, 88, 91, 93, 100, 104, 108], MTA – OA [32], MTA – Insulin [32], MARS-5 [77], ARMS-12 [44, 106, 123, 132], MTA [105], ARMS-7 [127], MEDS [114], IADMAS [38], GMAS [128, 129, 134, 136], ProMAS [130], A-14 [131], 12-item questionnaire [66], and Mascard [113]	Relevance, Comprehensiveness, and Comprehensibility with inconsistent results	±	No information
	MGT [52]	Sufficient relevance, sufficient comprehensiveness and sufficient comprehensibility	+	Moderate
Structural validity	MMAS-8 [28, 35, 39, 40, 46, 47, 50, 55, 57, 61, 62, 67, 68, 73, 79, 80, 86–88, 93, 100, 108, 110]	CFA: Tested one and two factor solutions. One factor presented five studies ' + ', two studies '-' and one study '?'. Two factors presented two studies ' + '. EFA: Solutions varying from one to four factors with results ' + ', '-' and '?'. IRT: one study '-' and one study '?'	±	No information
	SMAQ [29, 63]	EFA: One factor solution in one study '?'. Two factor solution in one study '?'	?	No information
	MEDS [114]	CFA: One factor solution in one study ' + '	+	High
	MNPS [115]	CFA: One factor solution in one study ' + '	+	High
	DMAS-7 [31]	EFA: Three factor solution in one study ' + '	+	Moderate
	ARMS-12 [44, 106, 123, 132]	CFA: Two factor solution in one study ' + '. EFA: Two factor solution in two studies ' + ' and one study '-'. Three factor solution in one study '-'	±	No information
Structural validity	MGT [51, 52, 81, 125]	EFA: One factor solution in two studies ' + ', one study '-' and one study '?'. CFA: One factor solution in one study ' + '	±	No information
	LMAS-14 [71, 111]	EFA: Four factor solution in one study ' + '. Three factor solution in one study ' + '	±	No information
	MTA [72]	CFA: One factor solution in one study '-'	-	High
	MARS-5 [92, 117, 133]	CFA: One factor solution in one study ' + '. EFA: One factor solution in two studies '?'. IRT: One study ' + '	+	Moderate
	ARMS-10 [75]	EFA: Two factor solution in one study ' + '	+	Moderate
	ARMS-D [37]	EFA: Two factor solution in one study ' + '	+	Moderate
	GMAS [65, 119, 128, 129, 134, 135]	EFA: Three factor solution in three studies ' + '. CFA: Three factor solution in four studies ' + ' and one study '-'. IRT: One study ' + '	+	High
	ProMAS [130]	IRT: One study ' + '	+	High
	ARMS-7 [127]	EFA: Two factor solution in one study ' + '. CFA: Two factor solution in one study ' + '	+	High
	Mascard [113]	EFA: Three factor solution in one study '-'	-	Moderate
Internal consistency	MMAS-8 [28, 30, 35, 39–41, 46–48, 50, 55, 57, 58, 60–62, 67, 68, 73, 76, 79, 80, 84, 86–88, 91, 93, 94, 99–101, 104, 108, 121, 124]	Cronbach alpha lower than 0.7 in 22 studies. Cronbach alpha equal or higher than 0.7 in 14 studies. Sample size: 10,472	?	No information
	SMAQ [29, 63]	Cronbach alpha lower than 0.7 in two studies. Sample size: 66	?	No information
	MEDS [114]	Cronbach alpha equal or higher than 0.7 in one study. Sample size: 685	+	High
Internal consistency	MNPS [115]	Cronbach alpha equal or higher than 0.7 in one study. Sample size: 675	+	High
	DMAS-7 [31, 36]	Cronbach alpha lower than 0.7 in two studies. Sample size: 800	-	Moderate
	ARMS-12 [44, 106, 123, 132]	Cronbach alpha lower than 0.7 in at least one subscale in 3 studies. Cronbach alpha equal or higher than 0.7 in 1 study. Sample size: 1,220	?	No information
	MGT [51, 52, 54, 69, 81, 83, 89, 110, 125]	Cronbach alpha lower than 0.7 in seven studies. Cronbach alpha equal or higher than 0.7 in two studies. Sample size: 8,382	?	No information
	MTA – OA [32]	Cronbach alpha equal or higher than 0.7 in one study. Sample size: 90	?	No information
	MTA – Insulin [32]	Cronbach alpha lower than 0.7 in one study. Sample size: 90	?	No information
	LMAS-14 [71, 111]	Cronbach alpha lower than 0.7 in at least one subscale in one study. Cronbach alpha equal or higher than 0.7 in one study. Sample size: 577	?	No information
	MTA [42, 105, 126]	Cronbach alpha lower than 0.7 in two studies. Cronbach alpha equal or higher than 0.7 in one study. Sample size: 701	?	No information
	MARS-5 [77, 92, 117, 133]	Cronbach alpha lower than 0.7 in two studies. Cronbach alpha equal or higher than 0.7 in two studies. Sample size: 1,783	+	High
	A-14 [74, 131]	Cronbach alpha equal or higher than 0.7 in two studies. Sample size: 119	?	No information
Internal consistency	ARMS-10 [75]	Cronbach alpha equal or higher than 0.7 in one study. Sample size: 120	+	Very low
	MALMAS [33, 34, 45]	Cronbach alpha lower than 0.7 in three studies. Sample size: 279	?	No information
	ARMS-D [37]	Cronbach alpha equal or higher than 0.7 in one study. Sample size: 314	+	High
	IADMAS [38]	Cronbach alpha equal or higher than 0.7 in one study. Sample size: 80	?	No information
	GMAS [65, 119, 128, 129, 134, 136]	Cronbach alpha lower than 0.7 in at least one subscale in five studies. Cronbach alpha equal or higher than 0.7 in one study. Sample size: 1,487	-	High
	MAQ [97]	Cronbach alpha equal or higher than 0.7 in one study. Sample size: 20	?	No information
	ProMAS [130]	Cronbach alpha equal or higher than 0.7 in one study. Sample size: 370	+	High
	ARMS-7 [127]	Cronbach alpha lower than 0.7 in at least one subscale in one study. Sample size: 100	-	Moderate
	AS [112]	Cronbach alpha higher than 0.7 in one study. Sample size: 201	?	No information
	12-item questionnaire [66]	Cronbach alpha higher than 0.7 in one study. Sample size: 30	?	No information
	Mascard [113]	Cronbach alpha lower than 0.7 in one study. Sample size: 219	?	No information
Reliability	MMAS-8 [41, 46, 47, 50, 55, 60–62, 76, 79, 82, 86–88, 93, 94, 104]	ICC and/or kappa lower than 0.7 in three studies; ICC or kappa equal or higher than 0.7 in ten studies; four studies did not report ICC or Kappa; sample size: 1,211	+	Low
	ARMS-12 [123, 132]	ICC not reported (one study); ICC = 0.97 (one study). sample size: 295	+	Low
	MGT [52, 54, 69]	ICC not reported (three studies); sample size: 325	?	No information
	MARS-5 [77, 117]	ICC not reported (two studies); sample size: 291	?	No information
	ARMS-10 [75]	ICC = 0.86 (one study); sample size = 120	+	Low
	MALMAS [33, 34, 45]	ICC not reported (three studies); sample size: 273	?	No information
	IADMAS [38]	ICC not reported (one study); sample size: 24	?	No information
	GMAS [119, 128, 129, 136]	ICC range 0.26—0.78 (one study); ICC not reported (three studies); sample size: 673	-	Moderate
	MAQ [97]	ICC = 0.98 (one study); sample size = 20	+	Very low
	ARMS-7 [127]	ICC range 0.76—0.80 (one study); sample size: 100	+	Very low
Criterion validity	MMAS-8 [30, 39, 41, 43, 46–48, 53, 57–59, 67, 76, 79, 80, 84, 87, 90, 99–101, 103, 108, 121, 124]	AUC lower than 0.7 in four studies. Correlation coefficient lower than 0.7 in nine studies. Sensititivity and/or specificity equal or lower than 50% in seven studies. Sensititivity and specificity higher than 50% in eight studies. Not presented the expected measures in three studies	-	Low
Criterion validity	SMAQ [29, 63]	Correlation coefficient lower than 0.7 in one study. Not presented the expected measures in one study	-	Very low
	MEDS [114]	Not presented the expected measures (one study)	?	No information
	MNPS [115]	Not presented the expected measures (one study)	?	No information
	DMAS-7 [31, 36]	AUC lower than 0.7 in two studies	-	High
	ARMS-12 [44, 106, 123]	AUC equal or higher than 0.7 in one study. Correlation coefficient lower than 0.7 in one study. Not presented the expected measures (one study)	±	No information
	MGT [51, 53, 54, 56, 69, 78, 81, 85, 89, 95, 96, 102, 107, 110, 118, 122]	AUC lower than 0.7 in three studies. Correlation coefficient lower than 0.7 in three studies. Sensititivity and/or specificity equal or lower than 50% in ten studies. Not presented the expected measures in two studies	-	Low
	LMAS-14 [71]	Sensititivity and/or specificity equal or lower than 50% in one study	?	No information
	MTA [42, 120, 126]	AUC lower than 0.7 in one study. Correlation coefficient lower than 0.7 in two studies. Sensititivity and/or specificity equal or lower than 50% in one study. Sensititivity and specificity higher than 50% in one study	-	High
	MARS-5 [92, 102, 117]	AUC lower than 0.7 in one study. Correlation coefficient equal or higher than 0.7 in one study. Not presented the expected measures in one study	±	No information
	MALMAS [34, 45]	Correlation coefficient lower than 0.7 in one study. Sensititivity and/or specificity equal or lower than 50% in two studies	-	High
	ARMS-D [37]	Correlation coefficient lower than 0.7 in one study	-	High
Criterion validity	IADMAS [38]	Correlation coefficient lower than 0.7 in one study. Sensititivity and/or specificity equal or lower than 50% in one study	-	Moderate
	GMAS [119, 128, 129, 134]	Sensititivity and/or specificity higher than 50% in two studies. Not presented the expected measures in two studies	?	No information
	5-item questionnaire [109]	Correlation coefficient lower than 0.7 in one study. Sensititivity and/or specificity equal or lower than 50% in one study	-	High
	3-item questionnaire [137]	Sensititivity and/or specificity equal or lower than 50% in one study	?	No information
	Mascard [113]	Sensititivity and/or specificity equal or lower than 50% in one study	?	No information
Hypotheses testing	MMAS-8 [39, 41, 46–48, 50, 55, 60, 67, 73, 80, 86, 87, 99, 100, 103, 104]	27 out of 38 hypotheses confirmed (seventeen studies)	±	No information
	MEDS [114]	2 out of 2 hypotheses confirmed (one study)	+	High
	DMAS-7 [31, 36]	5 out of 5 hypotheses confirmed (two studies)	+	High
	ARMS-12 [44, 64, 116, 123]	17 out of 21 hypotheses confirmed (three studies)	+	High
	MGT [54, 69, 70, 89, 102, 118, 122]	8 out of 17 hypotheses confirmed (seven studies)	±	No information
	MTA – OA [32]	2 out of 2 hypotheses confirmed (one study)	+	Very low
	MTA – Insulin [32]	2 out of 2 hypotheses confirmed (one study)	+	Very low
	LMAS-14 [71]	1 out of 1 hypotheses confirmed (one study)	+	Low
	MTA [105, 120]	5 out of 12 hypotheses confirmed (two studies)	±	No information
Hypotheses testing	MARS-5 [77, 102, 117]	4 out of 6 hypotheses confirmed (three studies)	±	No information
	A-14 [74, 131]	3 out of 3 hypotheses confirmed (two studies)	+	High
	MALMAS [33, 34, 45]	10 out of 10 hypotheses confirmed (three studies)	+	Low
	ARMS-D [37]	9 out of 9 hypotheses confirmed (one study)	+	High
	IADMAS [38]	4 out of 4 hypotheses confirmed (one study)	+	Moderate
	GMAS [65, 119, 128, 129, 134, 136]	12 out of 14 hypotheses confirmed (six studies)	+	Moderate
	ProMAS [49, 130]	2 out of 2 hypotheses confirmed (two studies)	+	High
	5-item questionnaire [109]	2 out of 2 hypotheses confirmed (one study)	+	High
	AS [112]	2 out of 2 hypotheses confirmed (one study)	+	High
Responsiveness	MMAS-5 [98]	3 out of 3 hypotheses confirmed (one study)	+	High

*ARMS* Adherence to Refills and Medication Scale, *AS* Adherence Scale, *AUC* Area under the curve, *CFA* Confirmatory factor analysis, *DMAS-7* 7-item Diabetes Medication Adherence Scale, *GMAS* General Medication Adherence Scale, *IADMAS* Iraqi Anti-Diabetic Medication Adherence Scale, *ICC* Intraclass correlation coefficient, *IRT* Item response theory, *LMAS-14* Fourteen-item Lebanese Medication Adherence Scale, *MALMAS* Malaysian Medication Adherence Scale, *MAQ* Medication Adherence Questionnaire, *MARS-5* 5-item Medication Adherence Report Scale, *Mascard* Medication Adherence Scale in Cardiovascular disorders, *MEDS* Medication Adherence Estimation and Differentiation Scale, *MGT* Morisky-Green test, *MMAS-5* 5-item adapted Morisky Medication Adherence Scale, *MMAS-8* 8-item Morisky Medication Adherence Scale, *MNPS* Medication Non-persistence Scale, *MTA* Measurement of Treatment Adherence, *MTA-Insulin* Measurement of Treatment Adherence – Insulin, *MTA-OA* MTA-Oral Antidiabetics, *NPV* Negative predictive value, *PPV* Positive predictive value, *PROM* Patient-reported outcome measures, *ProMAS* Probabilistic Medication Adherence Scale, *SMAQ* Simplified Medication Adherence Questionnaire, “ + ” = Sufficient; “ ± ” = Inconsistent; “ − ” = Insufficient; “?” = Indeterminate

### Content validity

The content validity resulted in overall ratings per PROM for relevance, comprehensiveness and comprehensibility, and overall content validity of the PROM. The indeterminate ratings for development or content validity studies were ignored in the overall rating assignment (*n* = 30). All the PROM development studies were classified as having inadequate methodological quality except the studies that developed the PROMs ProMAS [130]. and Mascard [113]. which were classified as having doubtful methodological quality. Very few studies assessed the target population's comprehensibility of the developed items through cognitive interviews or debriefing [38, 123]. According to the COSMIN, the criteria for recommending the use of a PROM is based on a sufficient content validity and at least low quality of evidence for internal consistency. The content validity encompasses the evaluation of aspects such as relevance, comprehensiveness, and comprehensibility of the PROM. The comprehensibility was the most often evaluated aspect in the records, but even studies that evaluated it had done so incompletely. Most of the studies assessed comprehensibility of the items [28, 41, 47, 57, 67, 68, 73, 76, 86, 88, 91, 93, 100, 104, 108, 113, 132, 134, 136], but only in a few of the studies participants were asked about the comprehensibility of response options or recall periods [28, 44, 91, 105, 106]. Relevance and comprehensiveness of PROMS were rarely evaluated among patients [32, 52, 67, 105] and expertise committee [32, 52, 61, 66, 105, 113, 114, 128]. In some aspects the PROMs, overall rating was based only on the rating of the reviewers. The evaluation of the risk of bias of the development and content validity studies resulted in studies being rated as doubtful or inadequate because some of the criteria evaluated in the COSMIN checklist were not clearly described in the records.

The PROM MGT showed moderate-quality evidence for sufficient content validity and the PROMs MMAS-8, MTA-OA, MTA – Insulin, MARS-5, ARMS-12, MTA, ARMS-7, MEDS, IADMAS, GMAS, ProMAS, A-14, 12-item questionnaire, and Mascard showed showed inconsistent content validity. The remaining PROMs included in the review did not have their content validity evaluated in the selected papers.

### Structural validity

The EFA was the most commonly applied statistical method in the evaluation of structural validity of the PROMs (ARMS-7, ARMS-10, ARMS-12, ARMS-D, DMAS-7, GMAS, LMAS-14, MARS-5, MGT, MMAS-8, SMAQ, and Mascard), [29, 31, 35, 37, 39, 40, 44, 46, 47, 50–52, 55, 57, 61, 62, 67, 68, 71, 75, 79, 81, 86–88, 93, 100, 106, 111, 113, 117, 119, 121, 123, 125, 127–129, 132, 133] followed by the confirmatory factor analysis (ARMS-7, ARMS-12, GMAS, MGT, MEDS, MMAS-8, MNPS, MTA, and MARS-5), [46, 52, 55, 57, 65, 72, 73, 80, 87, 88, 108, 114, 115, 119, 128, 129, 132–134] and the item response theory (MARS-5, GMAS, MMAS-8, and ProMAS) [28, 35, 92, 130, 135].

Regarding the assessment of the methodological quality of EFA, some studies were classified as having doubtful quality, since they did not report the rotation method used in the analysis [29, 39, 81, 86, 117].

In the evaluation of the EFA, some studies were classified as indeterminate because they did not report the percentage of variance explained [29, 35, 39, 40, 63, 81, 93, 117, 133] or the factor loadings [35, 81, 121]. One study did not report the results of the indices used to evaluate the confirmatory factor analysis [88] and another study [35] did not present the results of the indices of the item response theory analysis.

The PROMs MEDS, MNPS, GMAS, ProMAS, and ARMS-7 showed high-quality evidence for sufficient structural validity. The PROMs DMAS-7, MARS-5, ARMS-D, and ARMS-10 showed moderate-quality evidence for sufficient structural validity. Moderate and high-quality evidence for insufficient structural validity was observed for the Mascard and MTA, respectively.

However, the structural validity of the MMAS-8, MGT, LMAS-14, and the ARMS-12 were classified as inconsistent. The MMAS-8 presented results with one or two-factor solutions and also sufficient, insufficient, and indeterminate ratings. Similarly, the ARMS-12 presented sufficient and insufficient results in two or three-factor solutions, while the MGT presented only one-dimensional solution, but with sufficient, insufficient, and indeterminate ratings. LMAS-14 presented three or four -factor solutions. An overall rating indeterminate was attributed to SMAQ, since the included studies for this PROM were classified as indeterminate [29, 63]. The remaining PROMs included in the systematic review did not have their structural validity evaluated in the selected records.

### Internal consistency

Regarding the analysis of the internal consistency property, the original factor structure of the PROM was considered in order to evaluate if Cronbach's alpha should be calculated for the total scale and or subscales or domains. One included study [91] of the PROM MMAS-8 was classified as of doubtful methodological quality, since the authors excluded four items from the PROM because of the low Cronbach's alpha coefficient obtained, without considering other reliability or validity estimates. In two studies for the PROM A-14 [74, 131] it was not clear the number of the subscales of the PROM and in another three studies [29, 65, 75] that used the PROMs ARMS-10, SMAQ, and GMAS, the Cronbach’s alpha was not calculated for each of the subscales of the PROMs.

Four PROMs (MEDS, MNPS, ARMS-D, and ProMAS) showed high-quality evidence for sufficient internal consistency. However, very low-quality evidence for sufficient internal consistency for the ARMS-10 it was observed, while the PROMs DMAS-7 and ARMS-7 showed moderate quality evidence for insufficient internal consistency. Also GMAS showed low quality evidence for insufficient internal consistency. The internal consistency of the 15 PROMs (MMAS-8, SMAQ, ARMS-12, MGT, MTA-OA, MTA-Insulin, LMAS-14, MTA, A-14, MALMAS, IADMAS, MAQ, AS, 12-item questionnaire, and Mascard) were classified as indeterminate. The PROM MARS-5 had its internal consistency classified as inconsistent. The remaining PROMs included in the review did not have their internal consistency evaluated in the selected papers.

### Reliability

All included studies that evaluated the reliability of the PROMs (ARMS-7, ARMS-10, ARMS-12, GMAS, IADMAS, MALMAS, MAQ, MARS-5, MGT, and MMAS-8) were classified as of doubtful or inadequate methodological quality [33, 34, 38, 41, 45–47, 50, 52, 54, 55, 61, 62, 69, 75–77, 79, 82, 86–88, 93, 94, 97, 104, 117, 119, 123, 127–129, 132, 136] and did not provide enough data to address items 4 (“Did the professional(s) administer the measurement without knowledge of scores or values of other repeated measurement(s) in the same patients?”) and 5 (“Did the professional(s) assign scores or determine values without knowledge of the scores or values of other repeated measurement(s) in the same patients?”) of the risk of bias checklist [138]. The other included studies [46, 47, 52, 60, 86–88, 93, 117, 127] were classified as inadequate, since the evaluation of the item 3 of the risk of bias checklist (“Were the measurement conditions similar for the repeated measurements – except for the condition being evaluated as a source of variation?”) was considered inadequate.

Considering that the statistical analyses recommended to estimate reliability were intraclass correlation coefficient (ICC) and kappa, the results of some studies [33, 34, 38, 41, 45, 52, 54, 69, 76, 77, 79, 86, 117, 119, 123, 128, 136] were classified as indeterminate because Spearman's or Person's correlation coefficients were used to estimate the reliability of PROMs.

Regarding the best evidence of the reliability, the PROMs ARMS-10, ARMS-12, and MMAS-8 showed low-quality evidence for sufficient reliability, while the PROMs MAQ and ARMS-7 presented very low-quality evidence for sufficient reliability. The PROM GMAS showed moderate-quality evidence for insufficient reliability. The reliability of the PROMs MGT, MARS-5, MALMAS, and IADMAS were classified as indeterminate.

A meta-analysis to the reliability results was not performed, because the included studies did not show good methodological quality, according to the COSMIN guideline.

### Criterion validity

The analyses applied in the evaluation of the criterion validity were area under the curve, sensitivity, specificity, and some hypothesis tests. Some of the included studies that did not report sensitivity and specificity analyses of the PROMs ARMS-12 [44, 106, 123], GMAS [128, 129], MARS-5 [117], MMAS-8 [43, 121], and SMAQ [29], and one study regarding the PROM LMAS-14 [71] that did not provide the area under the curve analysis were classified as of inadequate methodological quality.

The PROMs DMAS-7, MTA, MALMAS, ARMS-D, and 5-item questionnaire showed high-quality evidence for insufficient criterion validity. MMAS-8 and MGT showed low-quality evidence for insufficient criterion validity. IADMAS and SMAQ presented moderate and very low-quality evidence for insufficient criterion validity, respectively. ARMS-12 and MARS-5 presented inconsistent results and MEDS, MNPS, LMAS-14, GMAS, 3-item questionnaire, and Mascard had its criterion validity classified as indeterminate. The criterion validity was not evaluated in the included papers regarding the remaining PROMs included in the review.

### Hypotheses testing

There were four included studies in which the methodological quality regarding the convergent validity of the PROMs was considered inadequate. Two of them that used the MMAS-8 [50, 103] were rated as inadequate because the comparator instrument had insufficient measurement properties. In the other two studies [32, 71] that used the PROMs LMAS-14, MTA-OA, and MTA-Insulin, the statistical tests applied were not optimal or appropriate. One study that used MALMAS was classified as having indeterminate quality, because the PROMs being correlated were not applied to the same participants [33].

Concerning the known‐groups validity, there was one included study for the PROM GMAS [128] that did not provide a description of the important characteristics of the groups being compared.

The analysis applied by the included studies were mainly correlation coefficients, regression models, and comparison and association tests.

The PROMs MEDS, DMAS-7, ARMS-12, A-14, ARMS-D, ProMAS, 5-item questionnaire, and AS showed high-quality evidence for sufficient construct validity. IADMAS and GMAS showed moderate-quality evidence for sufficient construct validity. LMAS-14 and MALMAS showed low-quality evidence for sufficient construct validity. MTA-OA and MTA-Insulin presented very low-quality evidence for sufficient construct validity. The PROMs MMAS-8, MGT, MTA, and MARS-5 showed inconsistent construct validity. The remaining PROMs included in the review did not have their construct validity evaluated in the selected papers.

### Responsiveness

Responsiveness was evaluated only for an adapted version of MMAS-8 composed by 5 items, nominated in this systematic review as MMAS-5, in a single study [98]. The study reported very good methodological quality and high-quality evidence for sufficient responsiveness.

### Meta-analysis

The meta-analysis was performed to pool the results of Cronbach’s Alpha of the included studies. Considering a Cronbach's Alpha equal to or higher than 0.7 to be satisfactory [13], the PROM MARS-5 showed high-quality evidence for sufficient internal consistency. GMAS showed high-quality evidence for insufficient internal consistency. The PROMs MMAS-8, ARMS-12, MTA, and MGT were classified as indeterminate because their structural validity was classified as inconsistent. The MALMAS was classified as indeterminate because it did not have its structural validity evaluated in the included studies. Values of I^2^ equal or higher than 50% and 75% indicates the presence of moderate and high heterogeneity, respectively [139]. Moderate or high heterogeneity was observed in the PROMs MMAS-8, ARMS-12, MGT, MARS-5, and GMAS (Table 5).

**Table 5:** Summarized results of the meta-analysis, heterogeneity, and quality of evidence of internal consistency of the PROMS included in this analysis

PROM	Pooled alpha (CI 95%)	Number of studies	Sample size	Q (*p*-value)	I^2^	Overall rating	Quality of evidence
MMAS-8	0.68 (0.64–0.72)	34	10,232	467.24 (< 0.0001)	92.94	?	No information
ARMS-12 (subscale 1)	0.88 (0.67; 0.96)	3	916	147.14 (< 0.0001)	98.64	?	No information
ARMS-12 (subscale 2)	0.70 (0.60; 0.78)	3	916	14.93 (0.0006)	86.60		
MGT	0.59 (0.50; 0.67)	9	8,382	133.76 (< 0.0001)	94.02	?	No information
MTA	0.66 (0.59; 0.72)	3	701	3.84 (0.1466)	47.92	?	No information
MARS-5	0.76 (0.67; 0.83)	4	1,783	92.40 (< 0.0001)	93.51	+	High
MALMAS	0.62 (0.54; 0.69)	3	279	1.72 (0.4233)	0.00	?	No information
GMAS (subscale 1)	0.76 (0.73; 0.79)	5	987	6.23 (0.1824)	35.79	-	High
GMAS (subscale 2)	0.73 (0.68; 0.78)	5	987	11.25 (0.0239)	64.44		
GMAS (subscale 3)	0.56 (0.38; 0.69)	5	987	50.63 (< 0.0001)	92.10		

*ARMS* Adherence to Refills and Medication Scale, *CI* Confidence interval, *GMAS* General Medication Adherence Scale, *MALMAS* Malaysian Medication Adherence Scale, *MARS-5* 5-item Medication Adherence Report Scale, *MGT* Morisky-Green test, *MMAS-8* 8-item Morisky Medication Adherence Scale, *MTA* Measurement of Treatment Adherence, *PROM* Patient-reported outcome measures, “ + ” = sufficient; “ − ” = Insufficient; “?” = Indeterminate

**Fig. 2:**
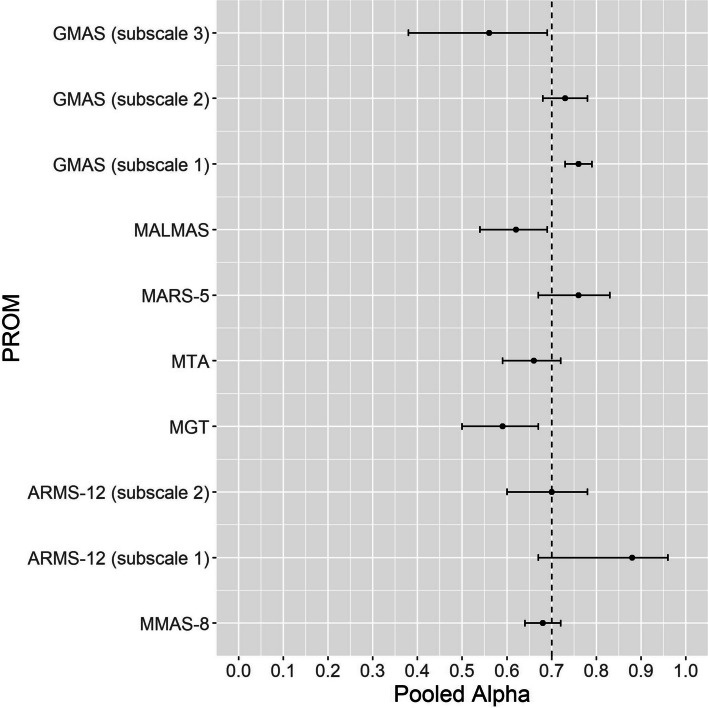
Pooled Cronbach's alpha estimates of the PROMs included in the meta-analyses. Note: ARMS = Adherence to Refills and Medication Scale; GMAS = General Medication Adherence Scale; MALMAS = Malaysian Medication Adherence Scale; MARS-5 = 5-item Medication Adherence Report Scale; MGT = Morisky-Green test; MMAS-8 = 8-item Morisky Medication Adherence Scale; MTA = Measurement of Treatment Adherence; PROM = Patient-reported outcome measures

The graphical representation of the pooled Alpha results for each of the included PROM in the meta-analyzes are shown in the Fig. 2.

### Interpretability and Feasibility

It was not possible to identify the information needed to evaluate the interpretability and feasibility in most of the included records. Considering that the evaluation of these aspects would be incomplete because of the lack of information, the reviewers decided not to evaluate these aspects.

### Recommendations for selecting a PROM

According to the results of our systematic review, none of the evaluated PROMs reached the criteria of category “a”, i.e., the results obtained across the studies can be trusted and the PROM can be recommended for use.

The PROMs MTA, GMAS, DMAS-7, MALMAS, ARMS-D, and 5-item questionnaire were categorized as not recommended for use (category “c”), because they presented high-quality evidence for at least one insufficient measurement property.

The remaining PROMs, i.e., MMAS-8, SMAQ, MEDS, MNPS, ARMS-12, MGT, MTA-OA, MTA-Insulin, LMAS-14, MARS-5, A-14, ARMS-10, IADMAS, MAQ, MMAS-5, ProMAS, ARMS‐7, 3-item questionnaire, AS, 12-item questionnaire, and Mascard were considered as having the potential to be recommended for use (category “b”) because they did not reach the criteria of the categories “a” or “c”.

## Discussion

The objective of this systematic review was to critically assess, compare, and synthesize the quality of the psychometric properties of PROMs for the assessment of medication adherence among patients with CVDs and/or T2DM.

To our knowledge, this is the first systematic review to assess the quality of the measurement properties of instruments that exclusively measure medication adherence using the COSMIN guideline. The results obtained allowed the identification of which instruments presented the best measurement properties in the population considered in this review. According to the COSMIN guidelines used in this systematic review, of the 27 PROMs extracted from the 110 studies included, none of the PROMs were recommended for use, 21 PROMs were considered to have the potential to be recommended and 6 PROMs were not recommended for use, as they did not meet the minimum criteria, i.e., demonstrated sufficient content validity and at least low quality of evidence for internal consistency. The summarized results of the meta-analysis and quality of evidence of internal consistency showed that, based on the results of three studies, only the MARS-5 has a high-quality evidence for a sufficient internal consistency.

As mentioned before, no systematic review was found in the literature evaluating the quality of the measurement properties of medication adherence PROMs, according to COSMIN guidelines specifically in patients with CVDs and/or T2DM. A recent study [140] analyzed systematic reviews in order to assess scope, validity, and reporting of PROMS of medication adherence in patients with T2DM. However, as previously noted, it included systematic reviews that do not specifically assess medication adherence and included studies that assessed factors related to medication adherence (self-efficacy, for an example), and reviews that used the PROM to evaluate interventions to promote medication adherence and did not apply a robust tool such as the COSMIN initiative guidelines for evaluating studies assessing the measurement properties of PROMs.

The main result of this review of reviews [140] was identifying that many PROMs have been translated into other languages without first presenting minimally adequate measurement properties in previous validation studies. It also pointed out that in some studies, the PROM was applied to a population without having been translated into the language of the country. The authors suggested that translated and adapted versions of PROMs that might in some way affect their items and/or subscales should be categorized separately from PROMs in their original format. In our review, studies of adapted versions of the PROMs were included, but they were not categorized separately. This may be a topic for future investigation of the PROMs evaluated in this review.

The findings of this systematic review about summarizing the data on the content validity of medication adherence PROMs showed that there were deficits and high heterogeneity of data in the included studies that investigated this property of the measure. The checklist for evaluation of methodological quality and the criteria for evaluation of the results related to the content validity of the PROMs proposed by the COSMIN initiative [141] are detailed in a large set of items, which were not covered by most of the studies included in the review. The reporting and data on the content validity of the included PROMs were extremely brief, with little information about the procedures performed, which hindered an adequate evaluation of this measurement property.

One difficulty observed in the evaluation of content validity was the absence of detailed information about the evaluation of relevance and comprehensiveness by the Expert Committee, as well as the comprehensibility by the target population in primary studies. Most of the included studies did not inform which one was investigated, and when they informed it, there was great heterogeneity in the way they were evaluated, implying inconsistent content validity. Our findings are congruent with a previous systematic review and meta-analysis that used the COSMIN guidelines to evaluate the evidence on measurement properties of the Hip disability and Osteoarthritis Outcome Score—Physical function Shortform (HOOS-P) and the Knee Injury and Osteoarthritis Outcome Score Physical function Shortform (KOOS-PS) [142], in which aspects such as the appropriateness of the response options and recall period and the relevance of the construct and context of the use were not evaluated in the primary studies, as in our review.

The internal consistency is an important measurement property that was included as an essential criterion to determine the recommendation for use of the PROM. However, the internal consistency of 15 PROMs in the included studies was rated as indeterminate. These results can be explained by the absence of at least low evidence for sufficient structural validity for these PROMS or because the structural validity of these measures was not performed in the included studies. The results of the meta-analysis and quality of evidence of internal consistency of the seven PROMS included in this analysis showed that only the MARS-5 has high-quality evidence for sufficient internal consistency, GMAS showed high-quality evidence for insufficient internal consistency and the other five PROMs showed indeterminate results. The meta-analysis resulted in many indeterminate results because of the structural validity results of the PROMs ARMS-12, MALMAS, MAT, MGT, and MMAS-8. The PROM ARMS-12 presented a pooled alpha that would result in a sufficient overall rating, but the structural validity limited this evaluation.

Regarding the evaluation of the structural validity, the PROMs MMAS-8, ARMS-12, MGT, and LMAS-14 were rated as inconsistent. This rating was attributed because different factor solutions were observed, i.e., different numbers of factors across the included records. Furthermore, four PROMs (MGT, MARS-5, MALMAS, and IADMAS) had their reliability rated as inconsistent because the results of the included records applied different coefficients (e.g. Pearson correlation coefficient) from the ones considered in the criteria stablished by the COSMIN initiative, i.e., ICC or Kappa.

As previously described, in the evaluation of the criterion validity, the review team considered that the obtained statistical results in the assessment of the relation between the PROMs and objective measures should be treated as a result of criterion validity, despite how the authors considered it in the primary studies. The reviewers considered that this change in the evaluation of the results would be beneficial as it would produce a standardization in the assessments. The findings showed that most of the evaluated PROMs presented an insufficient criterion validity and a sufficient overall rating in the hypothesis testing evaluation.

Another measurement property that had its evaluation compromised was the reliability. As previously mentioned, some items of the checklist for evaluation of the methodological quality were not described in the included studies, which resulted in studies being rated as having doubtful or inadequate methodological quality.

The evaluation of the measurement properties of the PROM’s included in this review indicated that none of the included PROMs could be considered trusted and recommended for use according to the criteria proposed by the COSMIN initiative. These results can be explained by the complexity of the medication adherence construct itself, which has made it challenging for researchers to obtain a PROM with good measurement properties [9]. The second aspect refers to the number of included studies in which each selected PROM was used. The evaluation of the measurement properties of a given PROM in several studies included in this review contributed to some measurement properties being rated as inconsistent because of the observed heterogeneity in the results of that PROM. The MMAS-8, for example, was the PROM for which the measurement properties were evaluated in 37 studies included in this review. Therefore with this large number of studies, heterogeneous results were likely for MMAS-8, which contributed to this PROM not being classified in the "a" category of recommendations. The other aspect that had a huge impact on the results was the evaluation of content validity, since this was one of the major gaps in the measurement properties of the medication adherence instruments, due to the lack of details of the data and analysis in primary studies, as previously mentioned. In addition to these issues identified when assessing PROM development and content validity, the fact that the methodological quality score of the measure properties considered the worst score of the COSMIN checklist may have contributed to downgrading the overall rating of the properties of the measure evaluated, as highlighted in previous studies [15, 143]. According to the COSMIN guideline, it is recommended to consider the worst score assigned to one of the assessment items of all COSMIN boxes, since methodological aspects considered poor in primary studies cannot be compensated by aspects considered to be good. In the guideline was highlighted that only significant flaws in study design or statistical analysis should be classified with the worst score [141]. Although, for some standars of the boxes, the worst possible response option was defined by COSMIN as "doubtful" or "adequate" rather than "inadequate" by the guideline, in order to reduce the impact of these assessments on the risk of bias, our results showed the influence of this criteria on the risk of bias assessment. Another point was the absence of a criteria for the evaluation of sensitivity and specificity results, when testing the criterion validity of PROMs. Furthermore, most of the studies included in the review were developed before the release of the guideline proposed by the COSMIN initiative, which may justify the fact that many of the assessment criteria proposed by the initiative were not performed or presented in the expected way in the primary studies evaluated. Thus, since the COSMIN guideline has not yet been widely used, future studies are recommended to refine its suitability, acceptability and quality.

The findings of our systematic review have implications for clinical practice, since it contributes to improve the evidence-based selection of PROMs in research and practice. Considering the perception of the patient about their adherence to medication treatment contributes to promoting a person-centered model of care, whose results are known to be promising in the management of chronic diseases. Therefore, the knowledge about which PROMs are evidence-based, recommended, or potentially recommended for use in clinical practice is crucial for positively impacting human health. The use of PROMs with high quality of evidence contributes to improving implementation science, because they have the best properties to measure the behavior, to evaluating the effect of interventions to optimize medication adherence, and to positive changes in chronic disease management and clinical practice.

### Strengths and limitations

One of the strengths of this systematic review was the careful application of the methodology proposed by the COSMIN initiative for the conduction of the systematic reviews in the evaluation of measurement properties of PROMs [21]. Another strength is the number of databases included in the literature searches which contributed to a more complete result. The review team should also be highlighted because it was composed of professionals from different areas, including nurses, dietitians, statistician, and researchers with expertise in research methodology and the use of PROMs. The different expertises of the team allowed for contributions related to the evaluation of the methodological rigor and statistical analyses of the studies, as well as those related to the evaluation of the pertinence and clinical relevance of different PROMS in measuring medication adherence.

A limitation of our study was the lack of detailed data in many studies to evaluate some of the measurement properties, especially content validity. This characteristic observed in the studies hampered the evaluation of many PROMs which resulted in many of them being poorly evaluated.

Another limitation was the high heterogeneity observed in the meta-analyses performed. Even if models with random effects were applied, the presence of high heterogeneity may bring limitations to the estimates obtained.

## Conclusions

The conclusion of this systematic review none of the evaluated PROMs could be considered trusted and recommended for use for patients with cardiovascular diseases and/or type 2 diabetes mellitus. However, another 21 PROMs have the potential to be recommended for use but need further studies to ensure their quality, according to the COSMIN guidelines for systematic reviews of PROMs. Furthermore, the findings showed that it is key to improve the reporting of results in PROM validation studies, especially with regard to content validity.

### Supplementary Information

**Additional file 1. **PRISMA Checklist.

**Additional file 2: **Search strategies.

**Additional file 3. **Studies’ characteristics.

**Additional file 4. **Quality of studies on the PROM development and content validity.

**Additional file 5. **Quality of studies on measurement properties.

## Data Availability

The datasets used and/or analyzed during the current study are available from the corresponding author on reasonable request.

## Abbreviations

ARMS: Adherence to Refills and Medication Scale
AS: Adherence Scale
AUC: Area under the curve
CFA: Confirmatory factor analysis
CINAHL: Cumulative index to nursing and allied health literature
COSMIN: Consensus-based standards for the selection of health measurement instruments
CVDs: Cardiovascular diseases
DMAS-7: 7-item Diabetes Medication Adherence Scale
EFA: Exploratory factor analysis
EMBASE: Excerpta medica database
GMAS: General Medication Adherence Scale
GRADE: Grading of recommendations assessment, development, and evaluation
HOOS-P: Hip disability and osteoarthritis outcome score - physical function shortform
IADMAS: Iraqi Anti-Diabetic Medication Adherence Scale
ICC: Intraclass correlation coefficient
IRT: Item response theory
KOOS-PS: Knee injury and osteoarthritis outcome score physical function shortform
LILACS: Literatura latino-americana e do Caribe em ciências da saúde
LMAS-14: Fourteen-item Lebanese Medication Adherence Scale
MALMAS: Malaysian Medication Adherence Scale
Mascard: Medication Adherence Scale in Cardiovascular disorders
MAQ: Medication Adherence Questionnaire
MARS-5: 5-item Medication Adherence Report Scale
MEDS: Medication Adherence Estimation and Differentiation Scale
MGT: Morisky-Green test
MMAS-5: 5-item adapted Morisky Medication Adherence Scale
MMAS-8: 8-item Morisky Medication Adherence Scale
MNPS: Medication Non-persistence Scale
MTA: Measurement of Treatment Adherence
MTA-Insulin: Measurement of Treatment Adherence - Insulin
MTA-OA: MTA-Oral Antidiabetics
NCDs: Noncommunicable diseases
NPV: Negative predictive value
ProMAS: Probabilistic Medication Adherence Scale
PPV: Positive predictive value
PRISMA: Preferred reporting items for systematic review and meta-analyses
PROMs: Patient-reported outcome measures
PROSPERO: International prospective register of systematic reviews
SMAQ: Simplified Medication Adherence Questionnaire
T2DM: Type 2 diabetes mellitus
WHO: World Health Organization
