# Supplementary material for: Quality of measurement properties of medication adherence instruments in cardiovascular diseases and type 2 diabetes mellitus: a systematic review and meta-analysis
Source: Syst Rev. 2023 Nov 22;12:222. doi: 10.1186/s13643-023-02340-z (PMC10664314; doi:10.1186/s13643-023-02340-z)
Supplement: Supplementary file 2 — Additional file 2: Search strategies. [file 13643_2023_2340_MOESM2_ESM.docx]

**Table S1 – Pubmed syntax**

| **Search** | **Query** |
| --- | --- |
| **#1** | (instrumentation[sh] OR methods[sh] OR "Validation Studies"[pt] OR "Comparative Study"[pt] OR "psychometrics"[MeSH] OR psychometr*[tiab] OR clinimetr*[tw] OR clinometr*[tw] OR "outcome assessment (health care)"[MeSH] OR "outcome assessment"[tiab] OR "outcome measure*"[tw] OR "observer variation"[MeSH] OR "observer variation"[tiab] OR "Health Status Indicators"[Mesh] OR "reproducibility of results"[MeSH] OR reproducib*[tiab] OR "discriminant analysis"[MeSH] OR reliab*[tiab] OR unreliab*[tiab] OR valid*[tiab] OR "coefficient of variation"[tiab] OR coefficient[tiab] OR homogeneity[tiab] OR homogeneous[tiab] OR "internal consistency"[tiab] OR (cronbach*[tiab] AND (alpha[tiab] OR alphas[tiab])) OR (item[tiab] AND (correlation*[tiab] OR selection*[tiab] OR reduction*[tiab])) OR agreement[tw] OR precision[tw] OR imprecision[tw] OR "precise values"[tw] OR test-retest[tiab] OR (test[tiab] AND retest[tiab]) OR (reliab*[tiab] AND (test[tiab] OR retest[tiab])) OR stability[tiab] OR interrater[tiab] OR inter-rater[tiab] OR intrarater[tiab] OR intra-rater[tiab] OR intertester[tiab] OR inter-tester[tiab] OR intratester[tiab] OR intra-tester[tiab] OR interobserver[tiab] OR inter-observer[tiab] OR intraobserver[tiab] OR intra-observer[tiab] OR intertechnician[tiab] OR inter-technician[tiab] OR intratechnician[tiab] OR intra-technician[tiab] OR interexaminer[tiab] OR inter-examiner[tiab] OR intraexaminer[tiab] OR intra-examiner[tiab] OR interassay[tiab] OR inter-assay[tiab] OR intraassay[tiab] OR intra-assay[tiab] OR interindividual[tiab] OR inter-individual[tiab] OR intraindividual[tiab] OR intra-individual[tiab] OR interparticipant[tiab] OR inter-participant[tiab] OR intraparticipant[tiab] OR intra-participant[tiab] OR kappa[tiab] OR kappa's[tiab] OR kappas[tiab] OR repeatab*[tw] OR ((replicab*[tw] OR repeated[tw]) AND (measure[tw] OR measures[tw] OR findings[tw] OR result[tw] OR results[tw] OR test[tw] OR tests[tw])) OR generaliza*[tiab] OR generalisa*[tiab] OR concordance[tiab] OR (intraclass[tiab] AND correlation*[tiab]) OR discriminative[tiab] OR "known group"[tiab] OR "factor analysis"[tiab] OR "factor analyses"[tiab] OR "factor structure"[tiab] OR "factor structures"[tiab] OR dimension*[tiab] OR subscale*[tiab] OR (multitrait[tiab] AND scaling[tiab] AND (analysis[tiab] OR analyses[tiab])) OR "item discriminant"[tiab] OR "interscale correlation*"[tiab] OR error[tiab] OR errors[tiab] OR "individual variability"[tiab] OR "interval variability"[tiab] OR "rate variability"[tiab] OR (variability[tiab] AND (analysis[tiab] OR values[tiab])) OR (uncertainty[tiab] AND (measurement[tiab] OR measuring[tiab])) OR "standard error of measurement"[tiab] OR sensitiv*[tiab] OR responsive*[tiab] OR (limit[tiab] AND detection[tiab]) OR "minimal detectable concentration"[tiab] OR interpretab*[tiab] OR ((minimal[tiab] OR minimally[tiab] OR clinical[tiab] OR clinically[tiab]) AND (important[tiab] OR significant[tiab] OR detectable[tiab]) AND (change[tiab] OR difference[tiab])) OR (small*[tiab] AND (real[tiab] OR detectable[tiab]) AND (change[tiab] OR difference[tiab])) OR "meaningful change"[tiab] OR "ceiling effect"[tiab] OR "floor effect"[tiab] OR "Item response model"[tiab] OR IRT[tiab] OR Rasch[tiab] OR "Differential item functioning"[tiab] OR DIF[tiab] OR "computer adaptive testing"[tiab] OR "item bank"[tiab] OR "cross-cultural equivalence"[tiab]) |
| **#2** | (((((((((((((((((((((((((((((((((((((((((((((((((((((((((((((((((((((((((((((((((Surveys and Questionnaires[MeSH Terms]) OR ("Surveys and Questionnaires"[Title/Abstract])) OR (Survey Methods[MeSH Terms])) OR ("Survey Methods"[Title/Abstract])) OR (Questionnaires and Surveys[MeSH Terms])) OR ("Questionnaires and Surveys"[Title/Abstract])) OR (Methods, Survey[MeSH Terms])) OR ("Methods, Survey"[Title/Abstract])) OR (Survey Method[MeSH Terms])) OR ("Survey Method"[Title/Abstract])) OR (Methodology, Survey[MeSH Terms])) OR ("Methodology, Survey"[Title/Abstract])) OR (Survey Methodology[MeSH Terms])) OR ("Survey Methodology"[Title/Abstract])) OR (Community Surveys[MeSH Terms])) OR ("Community Surveys"[Title/Abstract])) OR (Community Survey[MeSH Terms])) OR ("Community Survey"[Title/Abstract])) OR (Survey, Community[MeSH Terms])) OR ("Survey, Community"[Title/Abstract])) OR (Surveys, Community[MeSH Terms])) OR ("Surveys, Community"[Title/Abstract])) OR (Repeated Rounds of Survey[MeSH Terms])) OR ("Repeated Rounds of Survey"[Title/Abstract])) OR (Surveys[MeSH Terms])) OR (Surveys[Title/Abstract])) OR (Survey[MeSH Terms])) OR (Survey[Title/Abstract])) OR (Questionnaire Design[MeSH Terms])) OR ("Questionnaire Design"[Title/Abstract])) OR (Design, Questionnaire[MeSH Terms])) OR ("Design, Questionnaire"[Title/Abstract])) OR (Designs, Questionnaire[MeSH Terms])) OR ("Designs, Questionnaire"[Title/Abstract])) OR (Questionnaire Designs[MeSH Terms])) OR ("Questionnaire Designs"[Title/Abstract])) OR (Baseline Survey[MeSH Terms])) OR ("Baseline Survey"[Title/Abstract])) OR (Baseline Surveys[MeSH Terms])) OR ("Baseline Surveys"[Title/Abstract])) OR (Survey, Baseline[MeSH Terms])) OR ("Survey, Baseline"[Title/Abstract])) OR (Surveys, Baseline[MeSH Terms])) OR ("Surveys, Baseline"[Title/Abstract])) OR (Respondents[MeSH Terms])) OR (Respondents[Title/Abstract])) OR (Respondent[MeSH Terms])) OR (Respondent[Title/Abstract])) OR (Randomized Response Technique[MeSH Terms])) OR ("Randomized Response Technique"[Title/Abstract])) OR (Randomized Response Techniques[MeSH Terms])) OR ("Randomized Response Techniques"[Title/Abstract])) OR (Response Technique, Randomized[MeSH Terms])) OR ("Response Technique, Randomized"[Title/Abstract])) OR (Response Techniques, Randomized[MeSH Terms])) OR ("Response Techniques, Randomized"[Title/Abstract])) OR (Techniques, Randomized Response[MeSH Terms])) OR ("Techniques, Randomized Response"[Title/Abstract])) OR (Questionnaires[MeSH Terms])) OR (Questionnaires[Title/Abstract])) OR (Questionnaire[MeSH Terms])) OR (Questionnaire[Title/Abstract])) OR (Nonrespondents[MeSH Terms])) OR (Nonrespondents[Title/Abstract])) OR (Nonrespondent[MeSH Terms])) OR (Nonrespondent[Title/Abstract])) OR ((((((((((((((Patient Reported Outcome Measures[MeSH Terms]) OR ("Patient Reported Outcome Measures"[Title/Abstract])) OR (Patient Reported Outcomes[MeSH Terms])) OR ("Patient Reported Outcomes"[Title/Abstract])) OR (Outcome, Patient Reported[MeSH Terms])) OR ("Outcome, Patient Reported"[Title/Abstract])) OR (Outcomes, Patient Reported[MeSH Terms])) OR ("Outcomes, Patient Reported"[Title/Abstract])) OR (Reported Outcome, Patient[MeSH Terms])) OR ("Reported Outcome, Patient"[Title/Abstract])) OR (Reported Outcomes, Patient[MeSH Terms])) OR ("Reported Outcomes, Patient"[Title/Abstract])) OR (Patient Reported Outcome[MeSH Terms])) OR ("Patient Reported Outcome"[Title/Abstract]))) OR ((((((((Self Report[MeSH Terms]) OR ("Self Report"[Title/Abstract])) OR (Report, Self[MeSH Terms])) OR ("Report, Self"[Title/Abstract])) OR (Reports, Self[MeSH Terms])) OR ("Reports, Self"[Title/Abstract])) OR (Self Reports[MeSH Terms])) OR ("Self Reports"[Title/Abstract]))) OR (index)) OR (indices)) OR ("self-report")) OR ("self-report measures")) OR ("assessment tools")) OR ("patient-reported outcome")) OR ("measurement scale")) OR ("health measurement scale")) OR ("measure health outcomes")) OR (instrument)) OR ("measurement instrument")) OR (scale)) OR (measure)) OR (tool) |
| **#3** | (((((((((((((((((((((((((((((((((((((Medication Adherence[MeSH Terms]) OR "Medication Adherence"[Title/Abstract]) OR Adherence, Medication[MeSH Terms]) OR "Adherence, Medication"[Title/Abstract]) OR Medication Nonadherence[MeSH Terms]) OR "Medication Nonadherence"[Title/Abstract]) OR Nonadherence, Medication[MeSH Terms]) OR "Nonadherence, Medication"[Title/Abstract]) OR Medication Noncompliance[MeSH Terms]) OR "Medication Noncompliance"[Title/Abstract]) OR Noncompliance, Medication[MeSH Terms]) OR "Noncompliance, Medication"[Title/Abstract]) OR Medication Non-Adherence[MeSH Terms]) OR "Medication Non-Adherence"[Title/Abstract]) OR Medication Non Adherence[MeSH Terms]) OR "Medication Non Adherence"[Title/Abstract]) OR Non-Adherence, Medication[MeSH Terms]) OR "Non-Adherence, Medication"[Title/Abstract]) OR Medication Persistence[MeSH Terms]) OR "Medication Persistence"[Title/Abstract]) OR Persistence, Medication[MeSH Terms]) OR "Persistence, Medication"[Title/Abstract]) OR Medication Compliance[MeSH Terms]) OR "Medication Compliance"[Title/Abstract]) OR Compliance, Medication[MeSH Terms]) OR "Compliance, Medication"[Title/Abstract]) OR Medication Non-Compliance[MeSH Terms]) OR "Medication Non-Compliance"[Title/Abstract]) OR Medication Non Compliance[MeSH Terms]) OR "Medication Non Compliance"[Title/Abstract]) OR Non-Compliance, Medication[MeSH Terms]) OR "Non-Compliance, Medication"[Title/Abstract]) OR ((((((((((((((((((((((((((((((((((((((((((((((Patient Compliance[MeSH Terms]) OR "Patient Compliance"[Title/Abstract]) OR Compliance, Patient[MeSH Terms]) OR "Compliance, Patient"[Title/Abstract]) OR Patient Adherence[MeSH Terms]) OR "Patient Adherence"[Title/Abstract]) OR Adherence, Patient[MeSH Terms]) OR "Adherence, Patient"[Title/Abstract]) OR Patient Cooperation[MeSH Terms]) OR "Patient Cooperation"[Title/Abstract]) OR Cooperation, Patient[MeSH Terms]) OR "Cooperation, Patient"[Title/Abstract]) OR Patient Non-Compliance[MeSH Terms]) OR "Patient Non-Compliance"[Title/Abstract]) OR Non-Compliance, Patient[MeSH Terms]) OR "Non-Compliance, Patient"[Title/Abstract]) OR Patient Non Compliance[MeSH Terms]) OR "Patient Non Compliance"[Title/Abstract]) OR Patient Nonadherence[MeSH Terms]) OR "Patient Nonadherence"[Title/Abstract]) OR Nonadherence, Patient[MeSH Terms]) OR "Nonadherence, Patient"[Title/Abstract]) OR Patient Noncompliance[MeSH Terms]) OR "Patient Noncompliance"[Title/Abstract]) OR Noncompliance, Patient[MeSH Terms]) OR "Noncompliance, Patient"[Title/Abstract]) OR Patient Non-Adherence[MeSH Terms]) OR "Patient Non-Adherence"[Title/Abstract]) OR Non-Adherence, Patient[MeSH Terms]) OR "Non-Adherence, Patient"[Title/Abstract]) OR Patient Non Adherence[MeSH Terms]) OR "Patient Non Adherence"[Title/Abstract]) OR Treatment Compliance[MeSH Terms]) OR "Treatment Compliance"[Title/Abstract]) OR Compliance, Treatment[MeSH Terms]) OR "Compliance, Treatment"[Title/Abstract]) OR Treatment Compliances[MeSH Terms]) OR "Treatment Compliances"[Title/Abstract]) OR Therapeutic Compliance[MeSH Terms]) OR "Therapeutic Compliance"[Title/Abstract]) OR Compliance, Therapeutic[MeSH Terms]) OR "Compliance, Therapeutic"[Title/Abstract]) OR Compliances, Therapeutic[MeSH Terms]) OR "Compliances, Therapeutic"[Title/Abstract]) OR Therapeutic Compliances[MeSH Terms]) OR "Therapeutic Compliances"[Title/Abstract])) OR ("drug adherence")) OR ("drug compliance")) OR ("taking medication")) OR ("medication initiation")) OR ("medication implementation") |
| **#4** | (((((((((((((((((((((Cardiovascular Diseases[MeSH Terms]) OR "Cardiovascular Diseases"[Title/Abstract]) OR Cardiovascular Disease[MeSH Terms]) OR "Cardiovascular Disease"[Title/Abstract]) OR Disease, Cardiovascular[MeSH Terms]) OR "Diseases, Cardiovascular"[Title/Abstract]) OR Diseases, Cardiovascular[MeSH Terms]) OR "Diseases, Cardiovascular"[Title/Abstract])) OR ((((((((((((((((Heart Diseases[MeSH Terms]) OR "Heart Diseases"[Title/Abstract]) OR Disease, Heart[MeSH Terms]) OR "Disease, Heart"[Title/Abstract]) OR Diseases, Heart[MeSH Terms]) OR "Diseases, Heart"[Title/Abstract]) OR Heart Disease[MeSH Terms]) OR "Heart Disease"[Title/Abstract]) OR Cardiac Diseases[MeSH Terms]) OR "Cardiac Diseases"[Title/Abstract]) OR Cardiac Disease[MeSH Terms]) OR "Cardiac Disease"[Title/Abstract]) OR Disease, Cardiac[MeSH Terms]) OR "Disease, Cardiac"[Title/Abstract]) OR Diseases, Cardiac[MeSH Terms]) OR "Diseases, Cardiac"[Title/Abstract])) OR ((((((((Vascular Diseases[MeSH Terms]) OR "Vascular Diseases"[Title/Abstract]) OR Disease, Vascular[MeSH Terms]) OR "Disease, Vascular"[Title/Abstract]) OR Diseases, Vascular[MeSH Terms]) OR "Diseases, Vascular"[Title/Abstract]) OR Vascular Disease[MeSH Terms]) OR "Vascular Disease"[Title/Abstract])) OR ((((((((((((((((((((Coronary Disease[MeSH Terms]) OR "Coronary Disease"[Title/Abstract]) OR Coronary Diseases[MeSH Terms]) OR "Coronary Diseases"[Title/Abstract]) OR Disease, Coronary[MeSH Terms]) OR "Disease, Coronary"[Title/Abstract]) OR Diseases, Coronary[MeSH Terms]) OR "Diseases, Coronary"[Title/Abstract]) OR Coronary Heart Disease[MeSH Terms]) OR "Coronary Heart Disease"[Title/Abstract]) OR Coronary Heart Diseases[MeSH Terms]) OR "Coronary Heart Diseases"[Title/Abstract]) OR Disease, Coronary Heart[MeSH Terms]) OR "Disease, Coronary Heart"[Title/Abstract]) OR Diseases, Coronary Heart[MeSH Terms]) OR "Diseases, Coronary Heart"[Title/Abstract]) OR Heart Disease, Coronary[MeSH Terms]) OR "Heart Disease, Coronary"[Title/Abstract]) OR Heart Diseases, Coronary[MeSH Terms]) OR "Heart Diseases, Coronary"[Title/Abstract])) OR ((((((((((((((((((((((((((((((((((((((((((((((((((((Cerebrovascular Disorders[MeSH Terms]) OR "Cerebrovascular Disorders"[Title/Abstract]) OR Cerebrovascular Disorder[MeSH Terms]) OR "Cerebrovascular Disorder"[Title/Abstract]) OR Vascular Diseases, Intracranial[MeSH Terms]) OR "Vascular Diseases, Intracranial"[Title/Abstract]) OR Intracranial Vascular Disease[MeSH Terms]) OR "Intracranial Vascular Disease"[Title/Abstract]) OR Intracranial Vascular Diseases[MeSH Terms]) OR "Intracranial Vascular Diseases"[Title/Abstract]) OR Vascular Disease, Intracranial[MeSH Terms]) OR "Vascular Disease, Intracranial"[Title/Abstract]) OR Intracranial Vascular Disorders[MeSH Terms]) OR "Intracranial Vascular Disorders"[Title/Abstract]) OR Intracranial Vascular Disorder[MeSH Terms]) OR "Intracranial Vascular Disorder"[Title/Abstract]) OR Vascular Disorder, Intracranial[MeSH Terms]) OR "Vascular Disorder, Intracranial"[Title/Abstract]) OR Vascular Disorders, Intracranial[MeSH Terms]) OR "Vascular Disorders, Intracranial"[Title/Abstract]) OR Cerebrovascular Diseases[MeSH Terms]) OR "Cerebrovascular Diseases"[Title/Abstract]) OR Cerebrovascular Disease[MeSH Terms]) OR "Cerebrovascular Disease"[Title/Abstract]) OR Disease, Cerebrovascular[MeSH Terms]) OR "Disease, Cerebrovascular"[Title/Abstract]) OR Diseases, Cerebrovascular[MeSH Terms]) OR "Diseases, Cerebrovascular"[Title/Abstract]) OR Brain Vascular Disorders[MeSH Terms]) OR "Brain Vascular Disorders"[Title/Abstract]) OR Brain Vascular Disorder[MeSH Terms]) OR "Brain Vascular Disorder"[Title/Abstract]) OR Vascular Disorder, Brain[MeSH Terms]) OR "Vascular Disorder, Brain"[Title/Abstract]) OR Vascular Disorders, Brain[MeSH Terms]) OR "Vascular Disorders, Brain"[Title/Abstract]) OR Cerebrovascular Occlusion[MeSH Terms]) OR "Cerebrovascular Occlusion"[Title/Abstract]) OR Cerebrovascular Occlusions[MeSH Terms]) OR "Cerebrovascular Occlusions"[Title/Abstract]) OR Occlusion, Cerebrovascular[MeSH Terms]) OR "Occlusion, Cerebrovascular"[Title/Abstract]) OR Occlusions, Cerebrovascular[MeSH Terms]) OR "Occlusions, Cerebrovascular"[Title/Abstract]) OR Cerebrovascular Insufficiency[MeSH Terms]) OR "Cerebrovascular Insufficiency"[Title/Abstract]) OR Cerebrovascular Insufficiencies[MeSH Terms]) OR "Cerebrovascular Insufficiencies"[Title/Abstract]) OR Insufficiencies, Cerebrovascular[MeSH Terms]) OR "Insufficiencies, Cerebrovascular"[Title/Abstract]) OR Insufficiency, Cerebrovascular[MeSH Terms]) OR "Insufficiency, Cerebrovascular"[Title/Abstract])) OR ((((((((((((((((((((((((Peripheral Arterial Disease[MeSH Terms]) OR "Peripheral Arterial Disease"[Title/Abstract]) OR Arterial Disease, Peripheral[MeSH Terms]) OR "Arterial Disease, Peripheral"[Title/Abstract]) OR Arterial Diseases, Peripheral[MeSH Terms]) OR "Arterial Diseases, Peripheral"[Title/Abstract]) OR Disease, Peripheral Arterial[MeSH Terms]) OR "Disease, Peripheral Arterial"[Title/Abstract]) OR Diseases, Peripheral Arterial[MeSH Terms]) OR "Diseases, Peripheral Arterial"[Title/Abstract]) OR Peripheral Arterial Diseases[MeSH Terms]) OR "Peripheral Arterial Diseases"[Title/Abstract]) OR Peripheral Artery Disease[MeSH Terms]) OR "Peripheral Artery Disease"[Title/Abstract]) OR Artery Disease, Peripheral[MeSH Terms]) OR "Artery Disease, Peripheral"[Title/Abstract]) OR Artery Diseases, Peripheral[MeSH Terms]) OR "Artery Diseases, Peripheral"[Title/Abstract]) OR Disease, Peripheral Artery[MeSH Terms]) OR "Disease, Peripheral Artery"[Title/Abstract]) OR Diseases, Peripheral Artery[MeSH Terms]) OR "Diseases, Peripheral Artery"[Title/Abstract]) OR Peripheral Artery Diseases[MeSH Terms]) OR "Peripheral Artery Diseases"[Title/Abstract])) OR ((((((((((((((((((((((Rheumatic Heart Disease[MeSH Terms]) OR "Rheumatic Heart Disease"[Title/Abstract]) OR Disease, Rheumatic Heart[MeSH Terms]) OR "Disease, Rheumatic Heart"[Title/Abstract]) OR Diseases, Rheumatic Heart[MeSH Terms]) OR "Diseases, Rheumatic Heart"[Title/Abstract]) OR Heart Disease, Rheumatic[MeSH Terms]) OR "Heart Disease, Rheumatic"[Title/Abstract]) OR Heart Diseases, Rheumatic[MeSH Terms]) OR "Heart Diseases, Rheumatic"[Title/Abstract]) OR Rheumatic Heart Diseases[MeSH Terms]) OR "Rheumatic Heart Diseases"[Title/Abstract]) OR Bouillaud Disease[MeSH Terms]) OR "Bouillaud Disease"[Title/Abstract]) OR Disease, Bouillaud[MeSH Terms]) OR "Disease, Bouillaud"[Title/Abstract]) OR Bouillaud's Disease[MeSH Terms]) OR "Bouillaud's Disease"[Title/Abstract]) OR Bouillauds Disease[MeSH Terms]) OR "Bouillauds Disease"[Title/Abstract]) OR Disease, Bouillaud's[MeSH Terms]) OR "Disease, Bouillaud's"[Title/Abstract])) OR ((((((((((((((((((((((((((((((((((((((((((((((((((((Venous Thrombosis[MeSH Terms]) OR "Venous Thrombosis"[Title/Abstract]) OR Phlebothrombosis[MeSH Terms]) OR Phlebothrombosis[Title/Abstract]) OR Phlebothromboses[MeSH Terms]) OR Phlebothromboses[Title/Abstract]) OR Thrombosis, Venous[MeSH Terms]) OR "Thrombosis, Venous"[Title/Abstract]) OR Thromboses, Venous[MeSH Terms]) OR "Thromboses, Venous"[Title/Abstract]) OR Venous Thromboses[MeSH Terms]) OR "Venous Thromboses"[Title/Abstract]) OR Deep Vein Thrombosis[MeSH Terms]) OR "Deep Vein Thrombosis"[Title/Abstract]) OR Deep Vein Thromboses[MeSH Terms]) OR "Deep Vein Thromboses"[Title/Abstract]) OR Thromboses, Deep Vein[MeSH Terms]) OR "Thromboses, Deep Vein"[Title/Abstract]) OR Vein Thromboses, Deep[MeSH Terms]) OR "Vein Thromboses, Deep"[Title/Abstract]) OR Vein Thrombosis, Deep[MeSH Terms]) OR "Vein Thrombosis, Deep"[Title/Abstract]) OR Deep-Venous Thrombosis[MeSH Terms]) OR "Deep-Venous Thrombosis"[Title/Abstract]) OR Deep-Venous Thromboses[MeSH Terms]) OR "Deep-Venous Thromboses"[Title/Abstract]) OR Thromboses, Deep-Venous[MeSH Terms]) OR "Thromboses, Deep-Venous"[Title/Abstract]) OR Thrombosis, Deep-Venous[MeSH Terms]) OR "Thrombosis, Deep-Venous"[Title/Abstract]) OR Deep-Vein Thrombosis[MeSH Terms]) OR "Deep-Vein Thrombosis"[Title/Abstract]) OR Deep-Vein Thromboses[MeSH Terms]) OR "Deep-Vein Thromboses"[Title/Abstract]) OR Thromboses, Deep-Vein[MeSH Terms]) OR "Thromboses, Deep-Vein"[Title/Abstract]) OR Thrombosis, Deep-Vein[MeSH Terms]) OR "Thrombosis, Deep-Vein"[Title/Abstract]) OR Thrombosis, Deep Vein[MeSH Terms]) OR "Thrombosis, Deep Vein"[Title/Abstract]) OR Deep Venous Thrombosis[MeSH Terms]) OR "Deep Venous Thrombosis"[Title/Abstract]) OR Deep Venous Thromboses[MeSH Terms]) OR "Deep Venous Thromboses"[Title/Abstract]) OR Thromboses, Deep Venous[MeSH Terms]) OR "Thromboses, Deep Venous"[Title/Abstract]) OR Thrombosis, Deep Venous[MeSH Terms]) OR "Thrombosis, Deep Venous"[Title/Abstract]) OR Venous Thromboses, Deep[MeSH Terms]) OR "Venous Thromboses, Deep"[Title/Abstract]) OR Venous Thrombosis, Deep[MeSH Terms]) OR "Venous Thrombosis, Deep"[Title/Abstract])) OR ((((((((((((((((Pulmonary Embolism[MeSH Terms]) OR "Pulmonary Embolism"[Title/Abstract]) OR Pulmonary Embolisms[MeSH Terms]) OR "Pulmonary Embolisms"[Title/Abstract]) OR Embolism, Pulmonary[MeSH Terms]) OR "Embolism, Pulmonary"[Title/Abstract]) OR Embolisms, Pulmonary[MeSH Terms]) OR "Embolisms, Pulmonary"[Title/Abstract]) OR Pulmonary Thromboembolisms[MeSH Terms]) OR "Pulmonary Thromboembolisms"[Title/Abstract]) OR Pulmonary Thromboembolism[MeSH Terms]) OR "Pulmonary Thromboembolism"[Title/Abstract]) OR Thromboembolism, Pulmonary[MeSH Terms]) OR "Thromboembolism, Pulmonary"[Title/Abstract]) OR Thromboembolisms, Pulmonary[MeSH Terms]) OR "Thromboembolisms, Pulmonary"[Title/Abstract])) OR ((((((((((((((((((((((((((((((((((((((((((((((((((((((((((Stroke[MeSH Terms]) OR Stroke[Title/Abstract]) OR Strokes[MeSH Terms]) OR Strokes[Title/Abstract]) OR Cerebrovascular Accident[MeSH Terms]) OR "Cerebrovascular Accident"[Title/Abstract]) OR Cerebrovascular Accidents[MeSH Terms]) OR "Cerebrovascular Accidents"[Title/Abstract]) OR CVA (Cerebrovascular Accident)[MeSH Terms]) OR "CVA (Cerebrovascular Accident)"[Title/Abstract]) OR CVAs (Cerebrovascular Accident)[MeSH Terms]) OR "CVAs (Cerebrovascular Accident)"[Title/Abstract]) OR Cerebrovascular Apoplexy[MeSH Terms]) OR "Cerebrovascular Apoplexy"[Title/Abstract]) OR Apoplexy, Cerebrovascular[MeSH Terms]) OR "Apoplexy, Cerebrovascular"[Title/Abstract]) OR Vascular Accident, Brain[MeSH Terms]) OR "Vascular Accident, Brain"[Title/Abstract]) OR Brain Vascular Accident[MeSH Terms]) OR "Brain Vascular Accident"[Title/Abstract]) OR Brain Vascular Accidents[MeSH Terms]) OR "Brain Vascular Accidents"[Title/Abstract]) OR Vascular Accidents, Brain[MeSH Terms]) OR "Vascular Accidents, Brain"[Title/Abstract]) OR Cerebrovascular Stroke[MeSH Terms]) OR "Cerebrovascular Stroke"[Title/Abstract]) OR Cerebrovascular Strokes[MeSH Terms]) OR "Cerebrovascular Strokes"[Title/Abstract]) OR Stroke, Cerebrovascular[MeSH Terms]) OR "Stroke, Cerebrovascular"[Title/Abstract]) OR Strokes, Cerebrovascular[MeSH Terms]) OR "Strokes, Cerebrovascular"[Title/Abstract]) OR Apoplexy[MeSH Terms]) OR Apoplexy[Title/Abstract]) OR Cerebral Stroke[MeSH Terms]) OR "Cerebral Stroke"[Title/Abstract]) OR Cerebral Strokes[MeSH Terms]) OR "Cerebral Strokes"[Title/Abstract]) OR Stroke, Cerebral[MeSH Terms]) OR "Stroke, Cerebral"[Title/Abstract]) OR Strokes, Cerebral[MeSH Terms]) OR "Strokes, Cerebral"[Title/Abstract]) OR Stroke, Acute[MeSH Terms]) OR "Stroke, Acute"[Title/Abstract]) OR Acute Stroke[MeSH Terms]) OR "Acute Stroke"[Title/Abstract]) OR Acute Strokes[MeSH Terms]) OR "Acute Strokes"[Title/Abstract]) OR Strokes, Acute[MeSH Terms]) OR "Strokes, Acute"[Title/Abstract]) OR Cerebrovascular Accident, Acute[MeSH Terms]) OR "Cerebrovascular Accident, Acute"[Title/Abstract]) OR Acute Cerebrovascular Accident[MeSH Terms]) OR "Acute Cerebrovascular Accident"[Title/Abstract]) OR Acute Cerebrovascular Accidents[MeSH Terms]) OR "Acute Cerebrovascular Accidents"[Title/Abstract]) OR Cerebrovascular Accidents, Acute[MeSH Terms]) OR "Cerebrovascular Accidents, Acute"[Title/Abstract])) OR ((((((((((Hypertension[MeSH Terms]) OR Hypertension[Title/Abstract]) OR Blood Pressure, High[MeSH Terms]) OR "Blood Pressure, High"[Title/Abstract]) OR Blood Pressures, High[MeSH Terms]) OR "Blood Pressures, High"[Title/Abstract]) OR High Blood Pressure[MeSH Terms]) OR "High Blood Pressure"[Title/Abstract]) OR High Blood Pressures[MeSH Terms]) OR "High Blood Pressures"[Title/Abstract])) OR ((((((((((((((((((((((((((((Myocardial Infarction[MeSH Terms]) OR "Myocardial Infarction"[Title/Abstract]) OR Infarction, Myocardial[MeSH Terms]) OR "Infarction, Myocardial"[Title/Abstract]) OR Infarctions, Myocardial[MeSH Terms]) OR "Infarctions, Myocardial"[Title/Abstract]) OR Myocardial Infarctions[MeSH Terms]) OR "Myocardial Infarctions"[Title/Abstract]) OR Cardiovascular Stroke[MeSH Terms]) OR "Cardiovascular Stroke"[Title/Abstract]) OR Cardiovascular Strokes[MeSH Terms]) OR "Cardiovascular Strokes"[Title/Abstract]) OR Stroke, Cardiovascular[MeSH Terms]) OR "Stroke, Cardiovascular"[Title/Abstract]) OR Strokes, Cardiovascular[MeSH Terms]) OR "Strokes, Cardiovascular"[Title/Abstract]) OR Heart Attack[MeSH Terms]) OR "Heart Attack"[Title/Abstract]) OR Heart Attacks[MeSH Terms]) OR "Heart Attacks"[Title/Abstract]) OR Myocardial Infarct[MeSH Terms]) OR "Myocardial Infarct"[Title/Abstract]) OR Infarct, Myocardial[MeSH Terms]) OR "Infarct, Myocardial"[Title/Abstract]) OR Infarcts, Myocardial[MeSH Terms]) OR "Infarcts, Myocardial"[Title/Abstract]) OR Myocardial Infarcts[MeSH Terms]) OR "Myocardial Infarcts"[Title/Abstract])) OR ((((((((((((((((((((((((((((((Heart Failure[MeSH Terms]) OR "Heart Failure"[Title/Abstract]) OR Cardiac Failure[MeSH Terms]) OR "Cardiac Failure"[Title/Abstract]) OR Heart Decompensation[MeSH Terms]) OR "Heart Decompensation"[Title/Abstract]) OR Decompensation, Heart[MeSH Terms]) OR "Decompensation, Heart"[Title/Abstract]) OR Heart Failure, Right-Sided[MeSH Terms]) OR "Heart Failure, Right-Sided"[Title/Abstract]) OR Heart Failure, Right Sided[MeSH Terms]) OR "Heart Failure, Right Sided"[Title/Abstract]) OR Right-Sided Heart Failure[MeSH Terms]) OR "Right-Sided Heart Failure"[Title/Abstract]) OR Right Sided Heart Failure[MeSH Terms]) OR "Right Sided Heart Failure"[Title/Abstract]) OR Myocardial Failure[MeSH Terms]) OR "Myocardial Failure"[Title/Abstract]) OR Congestive Heart Failure[MeSH Terms]) OR "Congestive Heart Failure"[Title/Abstract]) OR Heart Failure, Congestive[MeSH Terms]) OR "Heart Failure, Congestive"[Title/Abstract]) OR Heart Failure, Left-Sided[MeSH Terms]) OR "Heart Failure, Left-Sided"[Title/Abstract]) OR Heart Failure, Left Sided[MeSH Terms]) OR "Heart Failure, Left Sided"[Title/Abstract]) OR Left-Sided Heart Failure[MeSH Terms]) OR "Left-Sided Heart Failure"[Title/Abstract]) OR Left Sided Heart Failure[MeSH Terms]) OR "Left Sided Heart Failure"[Title/Abstract])) OR ((((((((((((((((((((((((((((((((((((((((((((((((((((((((((((((((Diabetes Mellitus, Type 2[MeSH Terms]) OR "Diabetes Mellitus, Type 2"[Title/Abstract]) OR Diabetes Mellitus, Noninsulin-Dependent[MeSH Terms]) OR "Diabetes Mellitus, Noninsulin-Dependent"[Title/Abstract]) OR Diabetes Mellitus, Ketosis-Resistant[MeSH Terms]) OR "Diabetes Mellitus, Ketosis-Resistant"[Title/Abstract]) OR Diabetes Mellitus, Ketosis Resistant[MeSH Terms]) OR "Diabetes Mellitus, Ketosis Resistant"[Title/Abstract]) OR Ketosis-Resistant Diabetes Mellitus[MeSH Terms]) OR "Ketosis-Resistant Diabetes Mellitus"[Title/Abstract]) OR Diabetes Mellitus, Non Insulin Dependent[MeSH Terms]) OR "Diabetes Mellitus, Non Insulin Dependent"[Title/Abstract]) OR Diabetes Mellitus, Non-Insulin-Dependent[MeSH Terms]) OR "Diabetes Mellitus, Non-Insulin-Dependent"[Title/Abstract]) OR Non-Insulin-Dependent Diabetes Mellitus[MeSH Terms]) OR "Non-Insulin-Dependent Diabetes Mellitus"[Title/Abstract]) OR Diabetes Mellitus, Stable[MeSH Terms]) OR "Diabetes Mellitus, Stable"[Title/Abstract]) OR Stable Diabetes Mellitus[MeSH Terms]) OR "Stable Diabetes Mellitus"[Title/Abstract]) OR Diabetes Mellitus, Type II[MeSH Terms]) OR "Diabetes Mellitus, Type II"[Title/Abstract]) OR NIDDM[MeSH Terms]) OR NIDDM[Title/Abstract]) OR Diabetes Mellitus, Noninsulin Dependent[MeSH Terms]) OR "Diabetes Mellitus, Noninsulin Dependent"[Title/Abstract]) OR Diabetes Mellitus, Maturity-Onset[MeSH Terms]) OR "Diabetes Mellitus, Maturity-Onset"[Title/Abstract]) OR Diabetes Mellitus, Maturity Onset[MeSH Terms]) OR "Diabetes Mellitus, Maturity Onset"[Title/Abstract]) OR Maturity-Onset Diabetes Mellitus[MeSH Terms]) OR "Maturity-Onset Diabetes Mellitus"[Title/Abstract]) OR Maturity Onset Diabetes Mellitus[MeSH Terms]) OR "Maturity Onset Diabetes Mellitus"[Title/Abstract]) OR MODY[MeSH Terms]) OR MODY[Title/Abstract]) OR Diabetes Mellitus, Slow-Onset[MeSH Terms]) OR "Diabetes Mellitus, Slow-Onset"[Title/Abstract]) OR Diabetes Mellitus, Slow Onset[MeSH Terms]) OR "Diabetes Mellitus, Slow Onset"[Title/Abstract]) OR Slow-Onset Diabetes Mellitus[MeSH Terms]) OR "Slow-Onset Diabetes Mellitus"[Title/Abstract]) OR Type 2 Diabetes Mellitus[MeSH Terms]) OR "Type 2 Diabetes Mellitus"[Title/Abstract]) OR Noninsulin-Dependent Diabetes Mellitus[MeSH Terms]) OR "Noninsulin-Dependent Diabetes Mellitus"[Title/Abstract]) OR Noninsulin Dependent Diabetes Mellitus[MeSH Terms]) OR "Noninsulin Dependent Diabetes Mellitus"[Title/Abstract]) OR Maturity-Onset Diabetes[MeSH Terms]) OR "Maturity-Onset Diabetes"[Title/Abstract]) OR Diabetes, Maturity-Onset[MeSH Terms]) OR "Diabetes, Maturity-Onset"[Title/Abstract]) OR Maturity Onset Diabetes[MeSH Terms]) OR "Maturity Onset Diabetes"[Title/Abstract]) OR Type 2 Diabetes[MeSH Terms]) OR "Type 2 Diabetes"[Title/Abstract]) OR Diabetes, Type 2[MeSH Terms]) OR "Diabetes, Type 2"[Title/Abstract]) OR Diabetes Mellitus, Adult-Onset[MeSH Terms]) OR "Diabetes Mellitus, Adult-Onset"[Title/Abstract]) OR Adult-Onset Diabetes Mellitus[MeSH Terms]) OR "Adult-Onset Diabetes Mellitus"[Title/Abstract]) OR Diabetes Mellitus, Adult Onset[MeSH Terms]) OR "Diabetes Mellitus, Adult Onset"[Title/Abstract]) |
| **#5** | **#1 AND #2 AND #3 AND #4** |

**Table S2 – EMBASE syntax**

| **Search** | **Query** |
| --- | --- |
| **#1** | 'intermethod comparison'/exp OR 'intermethod comparison' OR 'data collection method'/exp OR 'data collection method' OR 'validation study'/exp OR 'validation study' OR 'feasibility study'/exp OR 'feasibility study' OR 'pilot study'/exp OR 'pilot study' OR 'psychometry'/exp OR 'psychometry' OR 'reproducibility'/exp OR 'reproducibility' OR reproducib*:ab,ti OR 'audit':ab,ti OR psychometr*:ab,ti OR clinimetr*:ab,ti OR clinometr*:ab,ti OR 'observer variation'/exp OR 'observer variation' OR 'observer variation':ab,ti OR 'discriminant analysis'/exp OR 'discriminant analysis' OR 'validity'/exp OR 'validity' OR reliab*:ab,ti OR valid*:ab,ti OR 'coefficient':ab,ti OR 'internal consistency':ab,ti OR (cronbach*:ab,ti AND ('alpha':ab,ti OR 'alphas':ab,ti)) OR 'item correlation':ab,ti OR 'item correlations':ab,ti OR 'item selection':ab,ti OR 'item selections':ab,ti OR 'item reduction':ab,ti OR 'item reductions':ab,ti OR 'agreement':ab,ti OR 'precision':ab,ti OR 'imprecision':ab,ti OR 'precise values':ab,ti OR 'test-retest':ab,ti OR ('test':ab,ti AND 'retest':ab,ti) OR (reliab*:ab,ti AND ('test':ab,ti OR 'retest':ab,ti)) OR 'stability':ab,ti OR 'interrater':ab,ti OR 'inter-rater':ab,ti OR 'intrarater':ab,ti OR 'intra-rater':ab,ti OR 'intertester':ab,ti OR 'inter-tester':ab,ti OR 'intratester':ab,ti OR 'interobeserver':ab,ti OR 'inter-observer':ab,ti OR 'intraobserver':ab,ti OR 'intertechnician':ab,ti OR 'inter-technician':ab,ti OR 'intratechnician':ab,ti OR 'interexaminer':ab,ti OR 'inter-examiner':ab,ti OR 'intraexaminer':ab,ti OR 'interassay':ab,ti OR 'inter-assay':ab,ti OR 'intraassay':ab,ti OR 'intra-assay':ab,ti OR 'interindividual':ab,ti OR 'inter-individual':ab,ti OR 'intraindividual':ab,ti OR 'intra-individual':ab,ti OR 'interparticipant':ab,ti OR 'inter-participant':ab,ti OR 'intraparticipant':ab,ti OR 'kappa':ab,ti OR 'kappas':ab,ti OR 'coefficient of variation':ab,ti OR repeatab*:ab,ti OR ((replicab*:ab,ti OR 'repeated':ab,ti) AND ('measure':ab,ti OR 'measures':ab,ti OR 'findings':ab,ti OR 'result':ab,ti OR 'results':ab,ti OR 'test':ab,ti OR 'tests':ab,ti)) OR generaliza*:ab,ti OR generalisa*:ab,ti OR 'concordance':ab,ti OR ('intraclass':ab,ti AND correlation*:ab,ti) OR 'discriminative':ab,ti OR 'known group':ab,ti OR 'factor analysis':ab,ti OR 'factor analyses':ab,ti OR 'factor structure':ab,ti OR 'factor structures':ab,ti OR 'dimensionality':ab,ti OR subscale*:ab,ti OR 'multitrait scaling analysis':ab,ti OR 'multitrait scaling analyses':ab,ti OR 'item discriminant':ab,ti OR 'interscale correlation':ab,ti OR 'interscale correlations':ab,ti OR (('error':ab,ti OR 'errors':ab,ti) AND (measure*:ab,ti OR correlat*:ab,ti OR evaluat*:ab,ti OR 'accuracy':ab,ti OR 'accurate':ab,ti OR 'precision':ab,ti OR 'mean':ab,ti)) OR 'individual variability':ab,ti OR 'interval variability':ab,ti OR 'rate variability':ab,ti OR 'variability analysis':ab,ti OR ('uncertainty':ab,ti AND ('measurement':ab,ti OR 'measuring':ab,ti)) OR 'standard error of measurement':ab,ti OR sensitiv*:ab,ti OR responsive*:ab,ti OR ('limit':ab,ti AND 'detection':ab,ti) OR 'minimal detectable concentration':ab,ti OR interpretab*:ab,ti OR (small*:ab,ti AND ('real':ab,ti OR 'detectable':ab,ti) AND ('change':ab,ti OR 'difference':ab,ti)) OR 'meaningful change':ab,ti OR 'minimal important change':ab,ti OR 'minimal important difference':ab,ti OR 'minimally important change':ab,ti OR 'minimally important difference':ab,ti OR 'minimal detectable change':ab,ti OR 'minimal detectable difference':ab,ti OR 'minimally detectable change':ab,ti OR 'minimally detectable difference':ab,ti OR 'minimal real change':ab,ti OR 'minimal real difference':ab,ti OR 'minimally real change':ab,ti OR 'minimally real difference':ab,ti OR 'ceiling effect':ab,ti OR 'floor effect':ab,ti OR 'item response model':ab,ti OR 'irt':ab,ti OR 'rasch':ab,ti OR 'differential item functioning':ab,ti OR 'dif':ab,ti OR 'computer adaptive testing':ab,ti OR 'item bank':ab,ti OR 'cross-cultural equivalence':ab,ti |
| **#2** | 'questionnaire'/exp OR 'questionnaire'/syn OR 'patient-reported outcome'/exp OR 'patient-reported outcome'/syn OR 'self report'/exp OR 'self report'/syn OR 'index':ab,ti OR 'indices':ab,ti OR 'self-report':ab,ti OR 'self-report measures':ab,ti OR 'assessment tools':ab,ti OR 'measurement scale':ab,ti OR 'health measurement scale':ab,ti OR 'measure health outcomes':ab,ti OR 'instrument':ab,ti OR 'measurement instrument':ab,ti OR 'scale':ab,ti OR 'measure':ab,ti OR 'tool'/exp OR 'tool'/syn |
| **#3** | 'medication compliance'/exp OR 'medication compliance'/syn OR 'patient compliance'/exp OR 'patient compliance'/syn OR 'taking medication':ab,ti OR 'medication initiation':ab,ti OR 'medication implementation':ab,ti |
| **#4** | 'cardiovascular disease'/exp OR 'cardiovascular disease'/syn OR 'heart disease'/exp OR 'heart disease'/syn OR 'vascular disease'/exp OR 'vascular disease'/syn OR 'coronary artery disease'/exp OR 'coronary artery disease'/syn OR 'cerebrovascular disease'/exp OR 'cerebrovascular disease'/syn OR 'peripheral occlusive artery disease'/exp OR 'peripheral occlusive artery disease'/syn OR 'rheumatic heart disease'/exp OR 'rheumatic heart disease'/syn OR 'vein thrombosis'/exp OR 'vein thrombosis'/syn OR 'lung embolism'/exp OR 'lung embolism'/syn OR 'cerebrovascular accident'/exp OR 'cerebrovascular accident'/syn OR 'hypertension'/exp OR 'hypertension'/syn OR 'heart infarction'/exp OR 'heart infarction'/syn OR 'heart failure'/exp OR 'heart failure'/syn OR 'non insulin dependent diabetes mellitus'/exp OR 'non insulin dependent diabetes mellitus'/syn |
| **#5** | **#1 AND #2 AND #3 AND #4** |

**Table S3 – LILACS syntax**

| **Search** | **Query** |
| --- | --- |
| **#1** | (instrumentation OR methods OR "Validation Studies" OR "Comparative Study" OR "psychometrics" OR psychometr* OR clinimetr* OR clinometr* OR "outcome assessment (health care)" OR "outcome assessment" OR "outcome measure*" OR "observer variation" OR "observer variation" OR "Health Status Indicators" OR "reproducibility of results" OR reproducib* OR "discriminant analysis" OR reliab* OR unreliab* OR valid* OR "coefficient of variation" OR coefficient OR homogeneity OR homogeneous OR "internal consistency" OR (cronbach* AND (alpha OR alphas)) OR (item AND (correlation* OR selection* OR reduction*)) OR agreement OR precision OR imprecision OR "precise values" OR test-retest OR (test AND retest) OR (reliab* AND (test OR retest)) OR stability OR interrater OR inter-rater OR intrarater OR intra-rater OR intertester OR inter-tester OR intratester OR intra-tester OR interobserver OR inter-observer OR intraobserver OR intra-observer OR intertechnician OR inter-technician OR intratechnician OR intra-technician OR interexaminer OR inter-examiner OR intraexaminer OR intra-examiner OR interassay OR inter-assay OR intraassay OR intra-assay OR interindividual OR inter-individual OR intraindividual OR intra-individual OR interparticipant OR inter-participant OR intraparticipant OR intra-participant OR kappa OR kappa's OR kappas OR repeatab* OR ((replicab* OR repeated) AND (measure OR measures OR findings OR result OR results OR test OR tests)) OR generaliza* OR generalisa* OR concordance OR (intraclass AND correlation*) OR discriminative OR "known group" OR "factor analysis" OR "factor analyses" OR "factor structure" OR "factor structures" OR dimension* OR subscale* OR (multitrait AND scaling AND (analysis OR analyses)) OR "item discriminant" OR "interscale correlation*" OR error OR errors OR "individual variability" OR "interval variability" OR "rate variability" OR (variability AND (analysis OR values)) OR (uncertainty AND (measurement OR measuring)) OR "standard error of measurement" OR sensitiv* OR responsive* OR (limit AND detection) OR "minimal detectable concentration" OR interpretab* OR ((minimal OR minimally OR clinical OR clinically) AND (important OR significant OR detectable) AND (change OR difference)) OR (small* AND (real OR detectable) AND (change OR difference)) OR "meaningful change" OR "ceiling effect" OR "floor effect" OR "Item response model" OR "IRT" OR Rasch OR "Differential item functioning" OR "DIF" OR "computer adaptive testing" OR "item bank" OR "cross-cultural equivalence") |
| **#2** | ("Surveys and Questionnaires" OR "Encuestas y Cuestionarios" OR "Inquéritos e Questionários") OR ("Patient Reported Outcome Measures" OR "Medición de Resultados Informados por el Paciente" OR "Medidas de Resultados Relatados pelo Paciente") OR ("Self Report" OR "Autoinforme" OR "Autorrelato") OR (Index OR Índice) OR Indices OR "Self-report" OR "Self-report measures" OR "Assessment tools" OR "Patient-reported outcome" OR "Measurement scale" OR "Health measurement scale" OR "Measure health outcomes" OR instrument OR "measurement instrument" OR scale OR measure OR tool |
| **#3** | ("Medication Adherence" OR "Cumplimiento de la Medicación" OR "Adesão à Medicação") OR ("Patient Compliance" OR "Cooperación del Paciente" OR "Cooperação do Paciente") OR "drug adherence" OR "drug compliance" OR "Taking medication" OR "Medication initiation" OR "Medication implementation" |
| **#4** | ("Cardiovascular Diseases" OR "Enfermedades Cardiovasculares" OR "Doenças Cardiovasculares") OR ("Heart Diseases" OR Cardiopatías OR Cardiopatias) OR ("Vascular Diseases" OR "Enfermedades Vasculares" OR "Doenças Vasculares") OR ("Coronary Disease" OR "Enfermedad Coronaria" OR "Doença das Coronárias") OR ("Cerebrovascular Disorders" OR "Trastornos Cerebrovasculares" OR "Transtornos Cerebrovasculares") OR ("Peripheral Arterial Disease" OR "Enfermedad Arterial Periférica" OR "Doença Arterial Periférica") OR ("Rheumatic Heart Disease" OR "Cardiopatía Reumática" OR "Cardiopatia Reumática") OR ("Venous Thrombosis" OR "Trombosis de la Vena" OR "Trombose Venosa") OR ("Pulmonary Embolism" OR "Embolia Pulmonar") OR (Stroke OR "Accidente Cerebrovascular" OR "Acidente Vascular Cerebral") OR (Hypertension OR Hipertensión OR Hipertensão) OR ("Myocardial Infarction" OR "Infarto del Miocardio" OR "Infarto do Miocárdio") OR ("Heart Failure" OR "Insuficiencia Cardíaca" OR "Insuficiência Cardíaca") OR ("Diabetes Mellitus, Type 2" OR "Diabetes Mellitus Tipo 2") |
| **#5** | **#1 AND #2 AND #3 AND #4** |

**Table S4 – PsycINFO syntax**

| **Search** | **Query** |
| --- | --- |
| **#1** | Any Field: (instrumentation OR methods OR "Validation Studies” OR "Comparative Study" OR "psychometrics" OR psychometr* OR clinimetr* OR clinometr* OR "outcome assessment (health care)" OR "outcome assessment" OR "outcome measure*" OR "observer variation" OR "observer variation" OR "Health Status Indicators" OR "reproducibility of results" OR reproducib* OR "discriminant analysis" OR reliab* OR unreliab* OR valid* OR "coefficient of variation" OR coefficient OR homogeneity OR homogeneous OR "internal consistency" OR (cronbach* AND (alpha OR alphas)) OR (item AND (correlation* OR selection* OR reduction*)) OR agreement OR precision OR imprecision OR "precise values" OR test-retest OR (test AND retest) OR (reliab* AND (test OR retest)) OR stability OR interrater OR inter-rater OR intrarater OR intra-rater OR intertester OR inter-tester OR intratester OR intra-tester OR interobserver OR inter-observer OR intraobserver OR intra-observer OR intertechnician OR inter-technician OR intratechnician OR intra-technician OR interexaminer OR inter-examiner OR intraexaminer OR intra-examiner OR interassay OR inter-assay OR intraassay OR intra-assay OR interindividual OR inter-individual OR intraindividual OR intra-individual OR interparticipant OR inter-participant OR intraparticipant OR intra-participant OR kappa OR kappa's OR kappas OR repeatab* OR ((replicab* OR repeated) AND (measure OR measures OR findings OR result OR results OR test OR tests)) OR generaliza* OR generalisa* OR concordance OR (intraclass AND correlation*) OR discriminative OR "known group" OR "factor analysis" OR "factor analyses" OR "factor structure" OR "factor structures" OR dimension* OR subscale* OR (multitrait AND scaling AND (analysis OR analyses)) OR "item discriminant" OR "interscale correlation*" OR error OR errors OR "individual variability" OR "interval variability" OR "rate variability" OR (variability AND (analysis OR values)) OR (uncertainty AND (measurement OR measuring)) OR "standard error of measurement" OR sensitiv* OR responsive* OR (limit AND detection) OR "minimal detectable concentration" OR interpretab* OR ((minimal OR minimally OR clinical OR clinically) AND (important OR significant OR detectable) AND (change OR difference)) OR (small* AND (real OR detectable) AND (change OR difference)) OR "meaningful change" OR "ceiling effect" OR "floor effect" OR "Item response model" OR “IRT” OR Rasch OR "Differential item functioning" OR “DIF” OR "computer adaptive testing" OR "item bank" OR "cross-cultural equivalence") |
| **#2** | ((Any Field: ("self-report measures")) OR (Any Field: ("assessment tools")) OR (Any Field: ("patient-reported outcome")) OR (Any Field: ("measurement scale")) OR (Any Field: ("health measurement scale")) OR (Any Field: ("measure health outcomes")) OR (Any Field: (instrument)) OR (Any Field: ("measurement instrument")) OR (Any Field: (scale)) OR (Any Field: (tool))) OR ((IndexTermsFilt: ("Measurement")) OR (Any Field: (measure))) OR ((IndexTermsFilt: ("Index (Testing)")) OR (Any Field: (index)) OR (Any Field: (indices))) OR ((IndexTermsFilt: ("Self-Report")) OR (Any Field: ("Self Report") OR Any Field: ("Report, Self") OR Any Field: ("Reports, Self") OR Any Field: ("Self Reports"))) OR ((IndexTermsFilt: ("Patient Reported Outcome Measures")) OR (Any Field: ("Patient Reported Outcome Measures") OR Any Field: ("Patient Reported Outcomes") OR Any Field: ("Outcome, Patient Reported") OR Any Field: ("Outcomes, Patient Reported") OR Any Field: ("Reported Outcome, Patient") OR Any Field: ("Reported Outcomes, Patient") OR Any Field: ("Patient Reported Outcome"))) OR ((IndexTermsFilt: ("Questionnaires")) OR (IndexTermsFilt: ("Surveys")) OR (Any Field: ("Surveys and Questionnaires") OR Any Field: ("Questionnaires and Surveys") OR Any Field: ("Survey Methods") OR Any Field: ("Methods, Survey") OR Any Field: ("Survey Method") OR Any Field: ("Methodology, Survey") OR Any Field: ("Survey Methodology") OR Any Field: ("Community Surveys") OR Any Field: ("Community Survey") OR Any Field: ("Survey, Community") OR Any Field: ("Surveys, Community") OR Any Field: ("Repeated Rounds of Survey") OR Any Field: (Surveys) OR Any Field: (Survey) OR Any Field: ("Questionnaire Design") OR Any Field: ("Design, Questionnaire") OR Any Field: ("Designs, Questionnaire") OR Any Field: ("Questionnaire Designs") OR Any Field: ("Baseline Survey") OR Any Field: ("Baseline Surveys") OR Any Field: ("Survey, Baseline") OR Any Field: ("Surveys, Baseline") OR Any Field: (Respondents) OR Any Field: (Respondent) OR Any Field: ("Randomized Response Technique") OR Any Field: ("Randomized Response Techniques") OR Any Field: ("Response Technique, Randomized") OR Any Field: ("Response Techniques, Randomized") OR Any Field: ("Techniques, Randomized Response") OR Any Field: (Questionnaires) OR Any Field: (Questionnaire) OR Any Field: (Nonrespondents) OR Any Field: (Nonrespondent))) |
| **#3** | Index Terms: {Treatment Compliance} OR Any Field: “Medication Adherence” OR “Adherence, Medication” OR “Medication Nonadherence” OR “Nonadherence, Medication” OR “Medication Noncompliance” OR “Noncompliance, Medication” OR “Medication Non-Adherence” OR “Medication Non Adherence” OR “Non-Adherence, Medication” OR “Medication Persistence” OR “Persistence, Medication” OR “Medication Compliance” OR “Compliance, Medication” OR “Medication Non-Compliance” OR “Medication Non Compliance” OR “Non-Compliance, Medication” OR Any Field: “Patient Compliance” OR “Compliance, Patient” OR “Patient Adherence” OR “Adherence, Patient” OR “Patient Cooperation” OR “Cooperation, Patient” OR “Patient Non-Compliance” OR “Non-Compliance, Patient” OR “Patient Non Compliance” OR “Patient Nonadherence” OR “Nonadherence, Patient” OR “Patient Noncompliance” OR “Noncompliance, Patient” OR “Patient Non-Adherence” OR “Non-Adherence, Patient” OR “Patient Non Adherence” OR “Treatment Compliance” OR “Compliance, Treatment” OR “Treatment Compliances” OR “Therapeutic Compliance” OR “Compliance, Therapeutic” OR “Compliances, Therapeutic” OR “Therapeutic Compliances” OR Any Field: "drug adherence" OR Any Field: "drug compliance" OR Any Field: "taking medication" OR Any Field: "medication initiation" OR Any Field: "medication implementation" |
| **#4** | ((IndexTermsFilt: ("CardioVascular Disorders")) OR (Any Field: ("Cardiovascular Diseases") OR Any Field: ("Cardiovascular Disease") OR Any Field: ("Disease, Cardiovascular") OR Any Field: ("Diseases, Cardiovascular")) OR (Any Field: ("Vascular Diseases") OR Any Field: ("Disease, Vascular") OR Any Field: ("Diseases, Vascular") OR Any Field: ("Vascular Disease")) OR (Any Field: ("Coronary Disease") OR Any Field: ("Coronary Diseases") OR Any Field: ("Disease, Coronary") OR Any Field: ("Diseases, Coronary") OR Any Field: ("Coronary Heart Disease") OR Any Field: ("Coronary Heart Diseases") OR Any Field: ("Disease, Coronary Heart") OR Any Field: ("Diseases, Coronary Heart") OR Any Field: ("Heart Disease, Coronary") OR Any Field: ("Heart Diseases, Coronary"))) OR ((IndexTermsFilt: ("Heart Disorders")) OR (Any Field: ("Heart Diseases") OR Any Field: ("Disease, Heart") OR Any Field: ("Diseases, Heart") OR Any Field: ("Heart Disease") OR Any Field: ("Cardiac Diseases") OR Any Field: ("Cardiac Disease") OR Any Field: ("Disease, Cardiac") OR Any Field: ("Diseases, Cardiac"))) OR ((IndexTermsFilt: ("Cerebrovascular Disorders")) OR (Any Field: ("Cerebrovascular Disorders") OR Any Field: ("Cerebrovascular Disorder") OR Any Field: ("Vascular Diseases, Intracranial") OR Any Field: ("Intracranial Vascular Disease") OR Any Field: ("Intracranial Vascular Diseases") OR Any Field: ("Vascular Disease, Intracranial") OR Any Field: ("Intracranial Vascular Disorders") OR Any Field: ("Intracranial Vascular Disorder") OR Any Field: ("Vascular Disorder, Intracranial") OR Any Field: ("Vascular Disorders, Intracranial") OR Any Field: ("Cerebrovascular Diseases") OR Any Field: ("Cerebrovascular Disease") OR Any Field: ("Disease, Cerebrovascular") OR Any Field: ("Diseases, Cerebrovascular") OR Any Field: ("Brain Vascular Disorders") OR Any Field: ("Brain Vascular Disorder") OR Any Field: ("Vascular Disorder, Brain") OR Any Field: ("Vascular Disorders, Brain") OR Any Field: ("Cerebrovascular Occlusion") OR Any Field: ("Cerebrovascular Occlusions") OR Any Field: ("Occlusion, Cerebrovascular") OR Any Field: ("Occlusions, Cerebrovascular") OR Any Field: ("Cerebrovascular Insufficiency") OR Any Field: ("Cerebrovascular Insufficiencies") OR Any Field: ("Insufficiencies, Cerebrovascular") OR Any Field: ("Insufficiency, Cerebrovascular")) OR (Any Field: ("Peripheral Arterial Disease") OR Any Field: ("Arterial Disease, Peripheral") OR Any Field: ("Arterial Diseases, Peripheral") OR Any Field: ("Disease, Peripheral Arterial") OR Any Field: ("Diseases, Peripheral Arterial") OR Any Field: ("Peripheral Arterial Diseases") OR Any Field: ("Peripheral Artery Disease") OR Any Field: ("Artery Disease, Peripheral") OR Any Field: ("Artery Diseases, Peripheral") OR Any Field: ("Disease, Peripheral Artery") OR Any Field: ("Diseases, Peripheral Artery") OR Any Field: ("Peripheral Artery Diseases")) OR (Any Field: ("Rheumatic Heart Disease") OR Any Field: ("Disease, Rheumatic Heart") OR Any Field: ("Diseases, Rheumatic Heart") OR Any Field: ("Heart Disease, Rheumatic") OR Any Field: ("Heart Diseases, Rheumatic") OR Any Field: ("Rheumatic Heart Diseases") OR Any Field: ("Bouillaud Disease") OR Any Field: ("Disease, Bouillaud") OR Any Field: ("Bouillaud's Disease") OR Any Field: ("Bouillauds Disease") OR Any Field: ("Disease, Bouillaud's"))) OR ((IndexTermsFilt: ("Thromboses") OR IndexTermsFilt: ("Coronary Thromboses")) OR (Any Field: ("Venous Thrombosis") OR Any Field: (Phlebothrombosis) OR Any Field: (Phlebothromboses) OR Any Field: ("Thrombosis, Venous") OR Any Field: ("Thromboses, Venous") OR Any Field: ("Venous Thromboses") OR Any Field: ("Deep Vein Thrombosis") OR Any Field: ("Deep Vein Thromboses") OR Any Field: ("Thromboses, Deep Vein") OR Any Field: ("Vein Thromboses, Deep") OR Any Field: ("Vein Thrombosis, Deep") OR Any Field: ("Deep-Venous Thrombosis") OR Any Field: ("Deep-Venous Thromboses") OR Any Field: ("Thromboses, Deep-Venous") OR Any Field: ("Thrombosis, Deep-Venous") OR Any Field: ("Deep-Vein Thrombosis") OR Any Field: ("Deep-Vein Thromboses") OR Any Field: ("Thromboses, Deep-Vein") OR Any Field: ("Thrombosis, Deep-Vein") OR Any Field: ("Thrombosis, Deep Vein") OR Any Field: ("Deep Venous Thrombosis") OR Any Field: ("Deep Venous Thromboses") OR Any Field: ("Thromboses, Deep Venous") OR Any Field: ("Thrombosis, Deep Venous") OR Any Field: ("Venous Thromboses, Deep") OR Any Field: ("Venous Thrombosis, Deep"))) OR ((IndexTermsFilt: ("Embolisms")) OR (Any Field: ("Pulmonary Embolism") OR Any Field: ("Pulmonary Embolisms") OR Any Field: ("Embolism, Pulmonary") OR Any Field: ("Embolisms, Pulmonary") OR Any Field: ("Pulmonary Thromboembolisms") OR Any Field: ("Pulmonary Thromboembolism") OR Any Field: ("Thromboembolism, Pulmonary") OR Any Field: ("Thromboembolisms, Pulmonary"))) OR ((IndexTermsFilt: ("Cerebrovascular Accidents")) OR (Any Field: (Stroke) OR Any Field: (Strokes) OR Any Field: ("Cerebrovascular Accident") OR Any Field: ("Cerebrovascular Accidents") OR Any Field: ("CVA (Cerebrovascular Accident)") OR Any Field: ("CVAs (Cerebrovascular Accident)") OR Any Field: ("Cerebrovascular Apoplexy") OR Any Field: ("Apoplexy, Cerebrovascular") OR Any Field: ("Vascular Accident, Brain") OR Any Field: ("Brain Vascular Accident") OR Any Field: ("Brain Vascular Accidents") OR Any Field: ("Vascular Accidents, Brain") OR Any Field: ("Cerebrovascular Stroke") OR Any Field: ("Cerebrovascular Strokes") OR Any Field: ("Stroke, Cerebrovascular") OR Any Field: ("Strokes, Cerebrovascular") OR Any Field: (Apoplexy) OR Any Field: ("Cerebral Stroke") OR Any Field: ("Cerebral Strokes") OR Any Field: ("Stroke, Cerebral") OR Any Field: ("Strokes, Cerebral") OR Any Field: ("Stroke, Acute") OR Any Field: ("Acute Stroke") OR Any Field: ("Acute Strokes") OR Any Field: ("Strokes, Acute") OR Any Field: ("Cerebrovascular Accident, Acute") OR Any Field: ("Acute Cerebrovascular Accident") OR Any Field: ("Acute Cerebrovascular Accidents") OR Any Field: ("Cerebrovascular Accidents, Acute"))) OR ((IndexTermsFilt: ("Hypertension")) OR (Any Field: (Hypertension) OR Any Field: ("Blood Pressure, High") OR Any Field: ("Blood Pressures, High") OR Any Field: ("High Blood Pressure") OR Any Field: ("High Blood Pressures"))) OR ((IndexTermsFilt: ("Myocardial Infarctions")) OR (Any Field: ("Myocardial Infarction") OR Any Field: ("Infarction, Myocardial") OR Any Field: ("Infarctions, Myocardial") OR Any Field: ("Myocardial Infarctions") OR Any Field: ("Cardiovascular Stroke") OR Any Field: ("Cardiovascular Strokes") OR Any Field: ("Stroke, Cardiovascular") OR Any Field: ("Strokes, Cardiovascular") OR Any Field: ("Heart Attack") OR Any Field: ("Heart Attacks") OR Any Field: ("Myocardial Infarct") OR Any Field: ("Infarct, Myocardial") OR Any Field: ("Infarcts, Myocardial") OR Any Field: ("Myocardial Infarcts"))) OR ((Any Field: ("Heart Failure") OR Any Field: ("Cardiac Failure") OR Any Field: ("Heart Decompensation") OR Any Field: ("Decompensation, Heart") OR Any Field: ("Heart Failure, Right-Sided") OR Any Field: ("Heart Failure, Right Sided") OR Any Field: ("Right-Sided Heart Failure") OR Any Field: ("Right Sided Heart Failure") OR Any Field: ("Myocardial Failure") OR Any Field: ("Congestive Heart Failure") OR Any Field: ("Heart Failure, Congestive") OR Any Field: ("Heart Failure, Left-Sided") OR Any Field: ("Heart Failure, Left Sided") OR Any Field: ("Left-Sided Heart Failure") OR Any Field: ("Left Sided Heart Failure"))) OR ((IndexTermsFilt: ("Type 2 Diabetes")) OR (Any Field: ("Diabetes Mellitus, Type 2") OR Any Field: ("Diabetes Mellitus, Noninsulin-Dependent") OR Any Field: ("Diabetes Mellitus, Noninsulin-Dependent") OR Any Field: ("Diabetes Mellitus, Ketosis-Resistant") OR Any Field: ("Diabetes Mellitus, Ketosis Resistant") OR Any Field: ("Ketosis-Resistant Diabetes Mellitus") OR Any Field: ("Diabetes Mellitus, Non Insulin Dependent") OR Any Field: ("Diabetes Mellitus, Non-Insulin-Dependent") OR Any Field: ("Non-Insulin-Dependent Diabetes Mellitus") OR Any Field: ("Diabetes Mellitus, Stable") OR Any Field: ("Stable Diabetes Mellitus") OR Any Field: ("Diabetes Mellitus, Type II") OR Any Field: (NIDDM) OR Any Field: ("Diabetes Mellitus, Noninsulin Dependent") OR Any Field: ("Diabetes Mellitus, Maturity-Onset") OR Any Field: ("Diabetes Mellitus, Maturity Onset") OR Any Field: ("Maturity-Onset Diabetes Mellitus") OR Any Field: ("Maturity Onset Diabetes Mellitus") OR Any Field: (MODY) OR Any Field: ("Diabetes Mellitus, Slow-Onset") OR Any Field: ("Diabetes Mellitus, Slow Onset") OR Any Field: ("Slow-Onset Diabetes Mellitus") OR Any Field: ("Type 2 Diabetes Mellitus") OR Any Field: ("Noninsulin-Dependent Diabetes Mellitus") OR Any Field: ("Noninsulin Dependent Diabetes Mellitus") OR Any Field: ("Maturity-Onset Diabetes") OR Any Field: ("Diabetes, Maturity-Onset") OR Any Field: ("Maturity Onset Diabetes") OR Any Field: ("Type 2 Diabetes") OR Any Field: ("Diabetes, Type 2") OR Any Field: ("Diabetes Mellitus, Adult-Onset") OR Any Field: ("Adult-Onset Diabetes Mellitus") OR Any Field: ("Diabetes Mellitus, Adult Onset"))) |
| **#5** | **#1 AND #2 AND #3 AND #4** |

**Table S5 – Scopus syntax**

| **Search** | **Query** |
| --- | --- |
| **#1** | TITLE-ABS-KEY ( ( instrumentation OR methods OR "Validation Studies" OR "Comparative Study" OR "psychometrics" OR psychometr? OR clinimetr? OR clinometr? OR "outcome assessment (health care)" OR "outcome assessment" OR "outcome measure?" OR "observer variation" OR "observer variation" OR "Health Status Indicators" OR "reproducibility of results" OR reproducib? OR "discriminant analysis" OR reliab? OR unreliab? OR valid? OR "coefficient of variation" OR coefficient OR homogeneity OR homogeneous OR "internal consistency" OR ( cronbach? AND ( alpha OR alphas ) ) OR ( item AND ( correlation? OR selection? OR reduction? ) ) OR agreement OR precision OR imprecision OR "precise values" OR test-retest OR ( test AND retest ) OR ( reliab? AND ( test OR retest ) ) OR stability OR interrater OR inter-rater OR intrarater OR intra-rater OR intertester OR inter-tester OR intratester OR intra-tester OR interobserver OR inter-observer OR intraobserver OR intra-observer OR intertechnician OR inter-technician OR intratechnician OR intra-technician OR interexaminer OR inter-examiner OR intraexaminer OR intra-examiner OR interassay OR inter-assay OR intraassay OR intra-assay OR interindividual OR inter-individual OR intraindividual OR intra-individual OR interparticipant OR inter-participant OR intraparticipant OR intra-participant OR kappa OR kappa's OR kappas OR repeatab? OR ( ( replicab? OR repeated ) AND ( measure OR measures OR findings OR result OR results OR test OR tests ) ) OR generaliza? OR generalisa? OR concordance OR ( intraclass AND correlation? ) OR discriminative OR "known group" OR "factor analysis" OR "factor analyses" OR "factor structure" OR "factor structures" OR dimension? OR subscale? OR ( multitrait AND scaling AND ( analysis OR analyses ) ) OR "item discriminant" OR "interscale correlation?" OR error OR errors OR "individual variability" OR "interval variability" OR "rate variability" OR ( variability AND ( analysis OR values ) ) OR ( uncertainty AND ( measurement OR measuring ) ) OR "standard error of measurement" OR sensitiv? OR responsive? OR ( limit AND detection ) OR "minimal detectable concentration" OR interpretab? OR ( ( minimal OR minimally OR clinical OR clinically ) AND ( important OR significant OR detectable ) AND ( change OR difference ) ) OR ( small? AND ( real OR detectable ) AND ( change OR difference ) ) OR "meaningful change" OR "ceiling effect" OR "floor effect" OR "Item response model" OR "IRT" OR rasch OR "Differential item functioning" OR "DIF" OR "computer adaptive testing" OR "item bank" OR "cross-cultural equivalence" ) ) |
| **#2** | ( TITLE-ABS-KEY ( "Surveys and Questionnaires" OR "Questionnaires and Surveys" OR "Survey Methods" OR "Methods, Survey" OR "Survey Method" OR "Methodology, Survey" OR "Survey Methodology" OR "Community Surveys" OR "Community Survey" OR "Survey, Community" OR "Surveys, Community" OR "Repeated Rounds of Survey" OR surveys OR survey OR "Questionnaire Design" OR "Design, Questionnaire" OR "Designs, Questionnaire" OR "Questionnaire Designs" OR "Baseline Survey" OR "Baseline Surveys" OR "Survey, Baseline" OR "Surveys, Baseline" OR respondents OR respondent OR "Randomized Response Technique" OR "Randomized Response Techniques" OR "Response Technique, Randomized" OR "Response Techniques, Randomized" OR "Techniques, Randomized Response" OR questionnaires OR questionnaire OR nonrespondents OR nonrespondent ) ) OR ( TITLE-ABS-KEY ( "Patient Reported Outcome Measures" OR "Patient Reported Outcomes" OR "Outcome, Patient Reported" OR "Outcomes, Patient Reported" OR "Reported Outcome, Patient" OR "Reported Outcomes, Patient" OR "Patient Reported Outcome" ) ) OR ( TITLE-ABS-KEY ( "Self Report" OR "Report, Self" OR "Reports, Self" OR "Self Reports" ) ) OR ( TITLE-ABS-KEY ( index ) ) OR ( TITLE-ABS-KEY ( indices ) ) OR ( TITLE-ABS-KEY ( "self-report" ) ) OR ( TITLE-ABS-KEY ( "self-report measures" ) ) OR ( TITLE-ABS-KEY ( "assessment tools" ) ) OR ( TITLE-ABS-KEY ( "patient-reported outcome" ) ) OR ( TITLE-ABS-KEY ( "measurement scale" ) ) OR ( TITLE-ABS-KEY ( "health measurement scale" ) ) OR ( TITLE-ABS-KEY ( "measure health outcomes" ) ) OR ( TITLE-ABS-KEY ( instrument ) ) OR ( TITLE-ABS-KEY ( "measurement instrument" ) ) OR ( TITLE-ABS-KEY ( scale ) ) OR ( TITLE-ABS-KEY ( measure ) ) OR ( TITLE-ABS-KEY ( tool ) ) |
| **#3** | ( TITLE-ABS-KEY ( "Medication Adherence" OR "Adherence, Medication" OR "Medication Nonadherence" OR "Nonadherence, Medication" OR "Medication Noncompliance" OR "Noncompliance, Medication" OR "Medication Non-Adherence" OR "Medication Non Adherence" OR "Non-Adherence, Medication" OR "Medication Persistence" OR "Persistence, Medication" OR "Medication Compliance" OR "Compliance, Medication" OR "Medication Non-Compliance" OR "Medication Non Compliance" OR "Non-Compliance, Medication" ) ) OR ( TITLE-ABS-KEY ( "Patient Compliance" OR "Compliance, Patient" OR "Patient Adherence" OR "Adherence, Patient" OR "Patient Cooperation" OR "Cooperation, Patient" OR "Patient Non-Compliance" OR "Non-Compliance, Patient" OR "Patient Non Compliance" OR "Patient Nonadherence" OR "Nonadherence, Patient" OR "Patient Noncompliance" OR "Noncompliance, Patient" OR "Patient Non-Adherence" OR "Non-Adherence, Patient" OR "Patient Non Adherence" OR "Treatment Compliance" OR "Compliance, Treatment" OR "Treatment Compliances" OR "Therapeutic Compliance" OR "Compliance, Therapeutic" OR "Compliances, Therapeutic" OR "Therapeutic Compliances" ) ) OR ( TITLE-ABS-KEY ( "drug adherence" ) ) OR ( TITLE-ABS-KEY ( "drug compliance" ) ) OR ( TITLE-ABS-KEY ( "taking medication" ) ) OR ( TITLE-ABS-KEY ( "medication initiation" ) ) OR ( TITLE-ABS-KEY ( "medication implementation" ) ) |
| **#4** | ( TITLE-ABS-KEY ( "Cardiovascular Diseases" OR "Cardiovascular Disease" OR "Disease, Cardiovascular" OR "Diseases, Cardiovascular" ) ) OR ( TITLE-ABS-KEY ( "Heart Diseases" OR "Disease, Heart" OR "Diseases, Heart" OR "Heart Disease" OR "Cardiac Diseases" OR "Cardiac Disease" OR "Disease, Cardiac" OR "Diseases, Cardiac" ) ) OR ( TITLE-ABS-KEY ( "Vascular Diseases" OR "Disease, Vascular" OR "Diseases, Vascular" OR "Vascular Disease" ) ) OR ( TITLE-ABS-KEY ( "Coronary Disease" OR "Coronary Diseases" OR "Disease, Coronary" OR "Diseases, Coronary" OR "Coronary Heart Disease" OR "Coronary Heart Diseases" OR "Disease, Coronary Heart" OR "Diseases, Coronary Heart" OR "Heart Disease, Coronary" OR "Heart Diseases, Coronary" ) ) OR ( TITLE-ABS-KEY ( "Cerebrovascular Disorders" OR "Cerebrovascular Disorder" OR "Vascular Diseases, Intracranial" OR "Intracranial Vascular Disease" OR "Intracranial Vascular Diseases" OR "Vascular Disease, Intracranial" OR "Intracranial Vascular Disorders" OR "Intracranial Vascular Disorder" OR "Vascular Disorder, Intracranial" OR "Vascular Disorders, Intracranial" OR "Cerebrovascular Diseases" OR "Cerebrovascular Disease" OR "Disease, Cerebrovascular" OR "Diseases, Cerebrovascular" OR "Brain Vascular Disorders" OR "Brain Vascular Disorder" OR "Vascular Disorder, Brain" OR "Vascular Disorders, Brain" OR "Cerebrovascular Occlusion" OR "Cerebrovascular Occlusions" OR "Occlusion, Cerebrovascular" OR "Occlusions, Cerebrovascular" OR "Cerebrovascular Insufficiency" OR "Cerebrovascular Insufficiencies" OR "Insufficiencies, Cerebrovascular" OR "Insufficiency, Cerebrovascular" ) ) OR ( TITLE-ABS-KEY ( "Peripheral Arterial Disease" OR "Arterial Disease, Peripheral" OR "Arterial Diseases, Peripheral" OR "Disease, Peripheral Arterial" OR "Diseases, Peripheral Arterial" OR "Peripheral Arterial Diseases" OR "Peripheral Artery Disease" OR "Artery Disease, Peripheral" OR "Artery Diseases, Peripheral" OR "Disease, Peripheral Artery" OR "Diseases, Peripheral Artery" OR "Peripheral Artery Diseases" ) ) OR ( TITLE-ABS-KEY ( "Rheumatic Heart Disease" OR "Disease, Rheumatic Heart" OR "Diseases, Rheumatic Heart" OR "Heart Disease, Rheumatic" OR "Heart Diseases, Rheumatic" OR "Rheumatic Heart Diseases" OR "Bouillaud Disease" OR "Disease, Bouillaud" OR "Bouillaud's Disease" OR "Bouillauds Disease" OR "Disease, Bouillaud's" ) ) OR ( TITLE-ABS-KEY ( "Venous Thrombosis" OR phlebothrombosis OR phlebothromboses OR "Thrombosis, Venous" OR "Thromboses, Venous" OR "Venous Thromboses" OR "Deep Vein Thrombosis" OR "Deep Vein Thromboses" OR "Thromboses, Deep Vein" OR "Vein Thromboses, Deep" OR "Vein Thrombosis, Deep" OR "Deep-Venous Thrombosis" OR "Deep-Venous Thromboses" OR "Thromboses, Deep-Venous" OR "Thrombosis, Deep-Venous" OR "Deep-Vein Thrombosis" OR "Deep-Vein Thromboses" OR "Thromboses, Deep-Vein" OR "Thrombosis, Deep-Vein" OR "Thrombosis, Deep Vein" OR "Deep Venous Thrombosis" OR "Deep Venous Thromboses" OR "Thromboses, Deep Venous" OR "Thrombosis, Deep Venous" OR "Venous Thromboses, Deep" OR "Venous Thrombosis, Deep" ) ) OR ( TITLE-ABS-KEY ( "Pulmonary Embolism" OR "Pulmonary Embolisms" OR "Embolism, Pulmonary" OR "Embolisms, Pulmonary" OR "Pulmonary Thromboembolisms" OR "Pulmonary Thromboembolism" OR "Thromboembolism, Pulmonary" OR "Thromboembolisms, Pulmonary" ) ) OR ( TITLE-ABS-KEY ( stroke OR strokes OR "Cerebrovascular Accident" OR "Cerebrovascular Accidents" OR "CVA (Cerebrovascular Accident)" OR "CVAs (Cerebrovascular Accident)" OR "Cerebrovascular Apoplexy" OR "Apoplexy, Cerebrovascular" OR "Vascular Accident, Brain" OR "Brain Vascular Accident" OR "Brain Vascular Accidents" OR "Vascular Accidents, Brain" OR "Cerebrovascular Stroke" OR "Cerebrovascular Strokes" OR "Stroke, Cerebrovascular" OR "Strokes, Cerebrovascular" OR apoplexy OR "Cerebral Stroke" OR "Cerebral Strokes" OR "Stroke, Cerebral" OR "Strokes, Cerebral" OR "Stroke, Acute" OR "Acute Stroke" OR "Acute Strokes" OR "Strokes, Acute" OR "Cerebrovascular Accident, Acute" OR "Acute Cerebrovascular Accident" OR "Acute Cerebrovascular Accidents" OR "Cerebrovascular Accidents, Acute" ) ) OR ( TITLE-ABS-KEY ( hypertension OR "Blood Pressure, High" OR "Blood Pressures, High" OR "High Blood Pressure" OR "High Blood Pressures" ) ) OR ( TITLE-ABS-KEY ( "Myocardial Infarction" OR "Infarction, Myocardial" OR "Infarctions, Myocardial" OR "Myocardial Infarctions" OR "Cardiovascular Stroke" OR "Cardiovascular Strokes" OR "Stroke, Cardiovascular" OR "Strokes, Cardiovascular" OR "Heart Attack" OR "Heart Attacks" OR "Myocardial Infarct" OR "Infarct, Myocardial" OR "Infarcts, Myocardial" OR "Myocardial Infarcts" ) ) OR ( TITLE-ABS-KEY ( "Heart Failure" OR "Cardiac Failure" OR "Heart Decompensation" OR "Decompensation, Heart" OR "Heart Failure, Right-Sided" OR "Heart Failure, Right Sided" OR "Right-Sided Heart Failure" OR "Right Sided Heart Failure" OR "Myocardial Failure" OR "Congestive Heart Failure" OR "Heart Failure, Congestive" OR "Heart Failure, Left-Sided" OR "Heart Failure, Left Sided" OR "Left-Sided Heart Failure" OR "Left Sided Heart Failure" ) ) OR ( TITLE-ABS-KEY ( "Diabetes Mellitus, Type 2" OR "Diabetes Mellitus, Noninsulin-Dependent" OR "Diabetes Mellitus, Noninsulin-Dependent" OR "Diabetes Mellitus, Ketosis-Resistant" OR "Diabetes Mellitus, Ketosis Resistant" OR "Ketosis-Resistant Diabetes Mellitus" OR "Diabetes Mellitus, Non Insulin Dependent" OR "Diabetes Mellitus, Non-Insulin-Dependent" OR "Non-Insulin-Dependent Diabetes Mellitus" OR "Diabetes Mellitus, Stable" OR "Stable Diabetes Mellitus" OR "Diabetes Mellitus, Type II" OR niddm OR "Diabetes Mellitus, Noninsulin Dependent" OR "Diabetes Mellitus, Maturity-Onset" OR "Diabetes Mellitus, Maturity Onset" OR "Maturity-Onset Diabetes Mellitus" OR "Maturity Onset Diabetes Mellitus" OR mody OR "Diabetes Mellitus, Slow-Onset" OR "Diabetes Mellitus, Slow Onset" OR "Slow-Onset Diabetes Mellitus" OR "Type 2 Diabetes Mellitus" OR "Noninsulin-Dependent Diabetes Mellitus" OR "Noninsulin Dependent Diabetes Mellitus" OR "Maturity-Onset Diabetes" OR "Diabetes, Maturity-Onset" OR "Maturity Onset Diabetes" OR "Type 2 Diabetes" OR "Diabetes, Type 2" OR "Diabetes Mellitus, Adult-Onset" OR "Adult-Onset Diabetes Mellitus" OR "Diabetes Mellitus, Adult Onset" ) ) |
| **#5** | **#1 AND #2 AND #3 AND #4** |

**Table S6 – Web of Science syntax**

| **Search** | **Query** |
| --- | --- |
| **#1** | (instrumentation OR methods OR "Validation Studies” OR "Comparative Study" OR "psychometrics" OR psychometr* OR clinimetr* OR clinometr* OR "outcome assessment (health care)" OR "outcome assessment" OR "outcome measure*" OR "observer variation" OR "observer variation" OR "Health Status Indicators" OR "reproducibility of results" OR reproducib* OR "discriminant analysis" OR reliab* OR unreliab* OR valid* OR "coefficient of variation" OR coefficient OR homogeneity OR homogeneous OR "internal consistency" OR (cronbach* AND (alpha OR alphas)) OR (item AND (correlation* OR selection* OR reduction*)) OR agreement OR precision OR imprecision OR "precise values" OR test-retest OR (test AND retest) OR (reliab* AND (test OR retest)) OR stability OR interrater OR inter-rater OR intrarater OR intra-rater OR intertester OR inter-tester OR intratester OR intra-tester OR interobserver OR inter-observer OR intraobserver OR intra-observer OR intertechnician OR inter-technician OR intratechnician OR intra-technician OR interexaminer OR inter-examiner OR intraexaminer OR intra-examiner OR interassay OR inter-assay OR intraassay OR intra-assay OR interindividual OR inter-individual OR intraindividual OR intra-individual OR interparticipant OR inter-participant OR intraparticipant OR intra-participant OR kappa OR kappa's OR kappas OR repeatab* OR ((replicab* OR repeated) AND (measure OR measures OR findings OR result OR results OR test OR tests)) OR generaliza* OR generalisa* OR concordance OR (intraclass AND correlation*) OR discriminative OR "known group" OR "factor analysis" OR "factor analyses" OR "factor structure" OR "factor structures" OR dimension* OR subscale* OR (multitrait AND scaling AND (analysis OR analyses)) OR "item discriminant" OR "interscale correlation*" OR error OR errors OR "individual variability" OR "interval variability" OR "rate variability" OR (variability AND (analysis OR values)) OR (uncertainty AND (measurement OR measuring)) OR "standard error of measurement" OR sensitiv* OR responsive* OR (limit AND detection) OR "minimal detectable concentration" OR interpretab* OR ((minimal OR minimally OR clinical OR clinically) AND (important OR significant OR detectable) AND (change OR difference)) OR (small* AND (real OR detectable) AND (change OR difference)) OR "meaningful change" OR "ceiling effect" OR "floor effect" OR "Item response model" OR “IRT” OR Rasch OR "Differential item functioning" OR “DIF” OR "computer adaptive testing" OR "item bank" OR "cross-cultural equivalence") |
| **#2** | (“Surveys and Questionnaires” OR “Questionnaires and Surveys” OR “Survey Methods” OR “Methods, Survey” OR “Survey Method” OR “Methodology, Survey” OR “Survey Methodology” OR “Community Surveys” OR “Community Survey” OR “Survey, Community” OR “Surveys, Community” OR “Repeated Rounds of Survey” OR Surveys OR Survey OR “Questionnaire Design” OR “Design, Questionnaire” OR “Designs, Questionnaire” OR “Questionnaire Designs” OR “Baseline Survey” OR “Baseline Surveys” OR “Survey, Baseline” OR “Surveys, Baseline” OR Respondents OR Respondent OR “Randomized Response Technique” OR “Randomized Response Techniques” OR “Response Technique, Randomized” OR “Response Techniques, Randomized” OR “Techniques, Randomized Response” OR Questionnaires OR Questionnaire OR Nonrespondents OR Nonrespondent) OR (“Patient Reported Outcome Measures” OR “Patient Reported Outcomes” OR “Outcome, Patient Reported” OR “Outcomes, Patient Reported” OR “Reported Outcome, Patient” OR “Reported Outcomes, Patient” OR “Patient Reported Outcome”) OR (“Self Report” OR “Report, Self” OR “Reports, Self” OR “Self Reports”) OR (index) OR (indices) OR ("self-report") OR ("self-report measures") OR (“assessment tools”) OR (“patient-reported outcome”) OR (“measurement scale”) OR (“health measurement scale”) OR (“measure health outcomes”) OR (instrument) OR ("measurement instrument") OR (scale) OR (measure) OR (tool) |
| **#3** | (“Medication Adherence” OR “Adherence, Medication” OR “Medication Nonadherence” OR “Nonadherence, Medication” OR “Medication Noncompliance” OR “Noncompliance, Medication” OR “Medication Non-Adherence” OR “Medication Non Adherence” OR “Non-Adherence, Medication” OR “Medication Persistence” OR “Persistence, Medication” OR “Medication Compliance” OR “Compliance, Medication” OR “Medication Non-Compliance” OR “Medication Non Compliance” OR “Non-Compliance, Medication”) OR (“Patient Compliance” OR “Compliance, Patient” OR “Patient Adherence” OR “Adherence, Patient” OR “Patient Cooperation” OR “Cooperation, Patient” OR “Patient Non-Compliance” OR “Non-Compliance, Patient” OR “Patient Non Compliance” OR “Patient Nonadherence” OR “Nonadherence, Patient” OR “Patient Noncompliance” OR “Noncompliance, Patient” OR “Patient Non-Adherence” OR “Non-Adherence, Patient” OR “Patient Non Adherence” OR “Treatment Compliance” OR “Compliance, Treatment” OR “Treatment Compliances” OR “Therapeutic Compliance” OR “Compliance, Therapeutic” OR “Compliances, Therapeutic” OR “Therapeutic Compliances”) OR ("drug adherence") OR ("drug compliance") OR ("taking medication") OR ("medication initiation") OR ("medication implementation") |
| **#4** | (“Cardiovascular Diseases” OR “Cardiovascular Disease” OR “Disease, Cardiovascular” OR “Diseases, Cardiovascular”) OR (“Heart Diseases” OR “Disease, Heart” OR “Diseases, Heart” OR “Heart Disease” OR “Cardiac Diseases” OR “Cardiac Disease” OR “Disease, Cardiac” OR “Diseases, Cardiac”) OR (“Vascular Diseases” OR “Disease, Vascular” OR “Diseases, Vascular” OR “Vascular Disease”) OR (“Coronary Disease” OR “Coronary Diseases” OR “Disease, Coronary” OR “Diseases, Coronary” OR “Coronary Heart Disease” OR “Coronary Heart Diseases” OR “Disease, Coronary Heart” OR “Diseases, Coronary Heart” OR “Heart Disease, Coronary” OR “Heart Diseases, Coronary”) OR (“Cerebrovascular Disorders” OR “Cerebrovascular Disorder” OR “Vascular Diseases, Intracranial” OR “Intracranial Vascular Disease” OR “Intracranial Vascular Diseases” OR “Vascular Disease, Intracranial” OR “Intracranial Vascular Disorders” OR “Intracranial Vascular Disorder” OR “Vascular Disorder, Intracranial” OR “Vascular Disorders, Intracranial” OR “Cerebrovascular Diseases” OR “Cerebrovascular Disease” OR “Disease, Cerebrovascular” OR “Diseases, Cerebrovascular” OR “Brain Vascular Disorders” OR “Brain Vascular Disorder” OR “Vascular Disorder, Brain” OR “Vascular Disorders, Brain” OR “Cerebrovascular Occlusion” OR “Cerebrovascular Occlusions” OR “Occlusion, Cerebrovascular” OR “Occlusions, Cerebrovascular” OR “Cerebrovascular Insufficiency” OR “Cerebrovascular Insufficiencies” OR “Insufficiencies, Cerebrovascular” OR “Insufficiency, Cerebrovascular”) OR (“Peripheral Arterial Disease” OR “Arterial Disease, Peripheral” OR “Arterial Diseases, Peripheral” OR “Disease, Peripheral Arterial” OR “Diseases, Peripheral Arterial” OR “Peripheral Arterial Diseases” OR “Peripheral Artery Disease” OR “Artery Disease, Peripheral” OR “Artery Diseases, Peripheral” OR “Disease, Peripheral Artery” OR “Diseases, Peripheral Artery” OR “Peripheral Artery Diseases”) OR (“Rheumatic Heart Disease” OR “Disease, Rheumatic Heart” OR “Diseases, Rheumatic Heart” OR “Heart Disease, Rheumatic” OR “Heart Diseases, Rheumatic” OR “Rheumatic Heart Diseases” OR “Bouillaud Disease” OR “Disease, Bouillaud” OR “Bouillaud's Disease” OR “Bouillauds Disease” OR “Disease, Bouillaud's”) OR (“Venous Thrombosis” OR Phlebothrombosis OR Phlebothromboses OR “Thrombosis, Venous” OR “Thromboses, Venous” OR “Venous Thromboses” OR “Deep Vein Thrombosis” OR “Deep Vein Thromboses” OR “Thromboses, Deep Vein” OR “Vein Thromboses, Deep” OR “Vein Thrombosis, Deep” OR “Deep-Venous Thrombosis” OR “Deep-Venous Thromboses” OR “Thromboses, Deep-Venous” OR “Thrombosis, Deep-Venous” OR “Deep-Vein Thrombosis” OR “Deep-Vein Thromboses” OR “Thromboses, Deep-Vein” OR “Thrombosis, Deep-Vein” OR “Thrombosis, Deep Vein” OR “Deep Venous Thrombosis” OR “Deep Venous Thromboses” OR “Thromboses, Deep Venous” OR “Thrombosis, Deep Venous” OR “Venous Thromboses, Deep” OR “Venous Thrombosis, Deep”) OR (“Pulmonary Embolism” OR “Pulmonary Embolisms” OR “Embolism, Pulmonary” OR “Embolisms, Pulmonary” OR “Pulmonary Thromboembolisms” OR “Pulmonary Thromboembolism” OR “Thromboembolism, Pulmonary” OR “Thromboembolisms, Pulmonary”) OR (Stroke OR Strokes OR “Cerebrovascular Accident” OR “Cerebrovascular Accidents” OR “CVA (Cerebrovascular Accident) ” OR “CVAs (Cerebrovascular Accident) ” OR “Cerebrovascular Apoplexy” OR “Apoplexy, Cerebrovascular” OR “Vascular Accident, Brain” OR “Brain Vascular Accident” OR “Brain Vascular Accidents” OR “Vascular Accidents, Brain” OR “Cerebrovascular Stroke” OR “Cerebrovascular Strokes” OR “Stroke, Cerebrovascular” OR “Strokes, Cerebrovascular” OR Apoplexy OR “Cerebral Stroke” OR “Cerebral Strokes” OR “Stroke, Cerebral” OR “Strokes, Cerebral” OR “Stroke, Acute” OR “Acute Stroke” OR “Acute Strokes” OR “Strokes, Acute” OR “Cerebrovascular Accident, Acute” OR “Acute Cerebrovascular Accident” OR “Acute Cerebrovascular Accidents” OR “Cerebrovascular Accidents, Acute”) OR (Hypertension OR “Blood Pressure, High” OR “Blood Pressures, High” OR “High Blood Pressure” OR “High Blood Pressures”) OR (“Myocardial Infarction” OR “Infarction, Myocardial” OR “Infarctions, Myocardial” OR “Myocardial Infarctions” OR “Cardiovascular Stroke” OR “Cardiovascular Strokes” OR “Stroke, Cardiovascular” OR “Strokes, Cardiovascular” OR “Heart Attack” OR “Heart Attacks” OR “Myocardial Infarct” OR “Infarct, Myocardial” OR “Infarcts, Myocardial” OR “Myocardial Infarcts”) OR (“Heart Failure” OR “Cardiac Failure” OR “Heart Decompensation” OR “Decompensation, Heart” OR “Heart Failure, Right-Sided” OR “Heart Failure, Right Sided” OR “Right-Sided Heart Failure” OR “Right Sided Heart Failure” OR “Myocardial Failure” OR “Congestive Heart Failure” OR “Heart Failure, Congestive” OR “Heart Failure, Left-Sided” OR “Heart Failure, Left Sided” OR “Left-Sided Heart Failure” OR “Left Sided Heart Failure”) OR ("Diabetes Mellitus, Type 2" OR "Diabetes Mellitus, Noninsulin-Dependent" OR “Diabetes Mellitus, Noninsulin-Dependent” OR “Diabetes Mellitus, Ketosis-Resistant” OR “Diabetes Mellitus, Ketosis Resistant” OR “Ketosis-Resistant Diabetes Mellitus” OR “Diabetes Mellitus, Non Insulin Dependent” OR “Diabetes Mellitus, Non-Insulin-Dependent” OR “Non-Insulin-Dependent Diabetes Mellitus” OR “Diabetes Mellitus, Stable” OR “Stable Diabetes Mellitus” OR “Diabetes Mellitus, Type II” OR NIDDM OR “Diabetes Mellitus, Noninsulin Dependent” OR “Diabetes Mellitus, Maturity-Onset” OR “Diabetes Mellitus, Maturity Onset” OR “Maturity-Onset Diabetes Mellitus” OR “Maturity Onset Diabetes Mellitus” OR MODY OR “Diabetes Mellitus, Slow-Onset” OR “Diabetes Mellitus, Slow Onset” OR “Slow-Onset Diabetes Mellitus” OR “Type 2 Diabetes Mellitus” OR “Noninsulin-Dependent Diabetes Mellitus” OR “Noninsulin Dependent Diabetes Mellitus” OR “Maturity-Onset Diabetes” OR “Diabetes, Maturity-Onset” OR “Maturity Onset Diabetes” OR “Type 2 Diabetes” OR “Diabetes, Type 2” OR “Diabetes Mellitus, Adult-Onset” OR “Adult-Onset Diabetes Mellitus” OR “Diabetes Mellitus, Adult Onset”) |
| **#5** | **#1 AND #2 AND #3 AND #4** |

**Table S7 – CINAHL syntax**

| **Search** | **Query** |
| --- | --- |
| **#1** | (MH “Psychometrics”) or ( TI psychometr* or AB psychometr* ) or ( TI clinimetr* or AB clinimetr* ) or ( TI clinometr* OR AB clinometr* ) or (MH “Outcome Assessment”) or ( TI outcome assessment or AB outcome assessment ) or ( TI outcome measure* or AB outcome measure* ) or (MH “Health Status Indicators”) or (MH “Reproducibility of Results”) or (MH “Discriminant Analysis”) or ( ( TI reproducib* or AB reproducib* ) or ( TI reliab* or AB reliab* ) or ( TI unreliab* or AB unreliab* ) ) or ( ( TI valid* or AB valid* ) or ( TI coefficient or AB coefficient ) or ( TI homogeneity or AB homogeneity ) ) or ( TI homogeneous or AB homogeneous ) or ( TI “coefficient of variation” or AB “coefficient of variation” ) or ( TI “internal consistency” or AB “internal consistency” ) or (MH “Internal Consistency+”) or (MH “Reliability+”) or (MH “Measurement Error+”) or (MH “Content Validity+”) or “hypothesis testing” or “structural validity” or “cross-cultural validity” or (MH “Criterion-Related Validity+”) or “responsiveness” or “interpretability” or ( TI reliab* or AB reliab* ) and ( (TI test or AB test) OR (TI retest or AB retest) ) or ( TI stability or AB stability ) or ( TI interrater or AB interrater ) or ( TI inter-rater or AB inter-rater ) or ( TI intrarater or AB intrarater ) or ( TI intra-rater or AB intrarater) or ( TI intertester or AB intertester) or (TI inter-tester or AB inter-tester) or ( TI intratester or AB intratester) or ( TI intra-tester or AB intra-tester) or ( TI interobserver or AB interobserver) or (TI inter-observer or AB inter-observer ) or ( TI intraobserver or AB intraobserver) or ( TI intra-observer or AB intra-observer) or ( TI intertechnician or AB intertechnician) or (TI inter-technician or AB inter-technician) or ( TI intratechnician or AB intratechnician ) or ( TI intra-technician or AB intra-technician ) or ( TI interexaminer or AB interexaminer ) or (TI inter-examiner or AB inter-examiner) or (TI intraexaminer or AB intraexaminer ) OR (TI intra-examiner or AB intra-examiner ) or (TI intra-examiner or AB intraexaminer ) or (TI interassay or AB interassay ) or ( TI inter-assay or AB inter-assay ) or ( TI intraassay or AB intraassay) or ( TI intra-assay or AB intra-assay ) or (TI interindividual or AB interindividual) or (TI inter-individual or AB inter-individual) OR (TI intraindividual or AB intraindividual) or (TI intra-individual or AB intra-individual) or (TI interparticipant or AB interparticipant) or (TI inter-participant or AB inter-participant ) or (TI intraparticipant or AB intraparticipant) or (TI intra-participant or AB intra-participant ) or (TI kappa or AB kappa) or (TI kappa’s or AB kappa’s ) or (TI kappas or AB kappas) or (TI repeatab* or AB repeatab*) or ( TI responsive* or AB responsive* ) or ( TI interpretab* or AB interpretab*) |
| **#2** | (MH "Questionnaires") OR (“Surveys and Questionnaires” OR “Questionnaires and Surveys” OR “Survey Methods” OR “Methods, Survey” OR “Survey Method” OR “Methodology, Survey” OR “Survey Methodology” OR “Community Surveys” OR “Community Survey” OR “Survey, Community” OR “Surveys, Community” OR “Repeated Rounds of Survey” OR Surveys OR Survey OR “Questionnaire Design” OR “Design, Questionnaire” OR “Designs, Questionnaire” OR “Questionnaire Designs” OR “Baseline Survey” OR “Baseline Surveys” OR “Survey, Baseline” OR “Surveys, Baseline” OR Respondents OR Respondent OR “Randomized Response Technique” OR “Randomized Response Techniques” OR “Response Technique, Randomized” OR “Response Techniques, Randomized” OR “Techniques, Randomized Response” OR Questionnaires OR Questionnaire OR Nonrespondents OR Nonrespondent) OR (MH "Patient-Reported Outcomes") OR (“Patient Reported Outcome Measures” OR “Patient Reported Outcomes” OR “Outcome, Patient Reported” OR “Outcomes, Patient Reported” OR “Reported Outcome, Patient” OR “Reported Outcomes, Patient” OR “Patient Reported Outcome”) OR (MH "Self Report") OR (“Self Report” OR “Report, Self” OR “Reports, Self” OR “Self Reports”) OR (index) OR (indices) OR ("self-report") OR ("self-report measures") OR (MH "Clinical Assessment Tools") OR (“assessment tools”) OR (“patient-reported outcome”) OR (“measurement scale”) OR (“health measurement scale”) OR (“measure health outcomes”) OR (MH "Instrument Construction") OR (MH "Research Instruments") OR (instrument) OR ("measurement instrument") OR (MH "Scales") OR (scale) OR (measure) OR (tool) |
| **#3** | (MH “Medication Compliance”) OR (“Medication Adherence” OR “Adherence, Medication” OR “Medication Nonadherence” OR “Nonadherence, Medication” OR “Medication Noncompliance” OR “Noncompliance, Medication” OR “Medication Non-Adherence” OR “Medication Non Adherence” OR “Non-Adherence, Medication” OR “Medication Persistence” OR “Persistence, Medication” OR “Medication Compliance” OR “Compliance, Medication” OR “Medication Non-Compliance” OR “Medication Non Compliance” OR “Non-Compliance, Medication”) OR (MH “Patient Compliance”) OR (“Patient Compliance” OR “Compliance, Patient” OR “Patient Adherence” OR “Adherence, Patient” OR “Patient Cooperation” OR “Cooperation, Patient” OR “Patient Non-Compliance” OR “Non-Compliance, Patient” OR “Patient Non Compliance” OR “Patient Nonadherence” OR “Nonadherence, Patient” OR “Patient Noncompliance” OR “Noncompliance, Patient” OR “Patient Non-Adherence” OR “Non-Adherence, Patient” OR “Patient Non Adherence” OR “Treatment Compliance” OR “Compliance, Treatment” OR “Treatment Compliances” OR “Therapeutic Compliance” OR “Compliance, Therapeutic” OR “Compliances, Therapeutic” OR “Therapeutic Compliances”) OR ("drug adherence") OR ("drug compliance") OR ("taking medication") OR ("medication initiation") OR ("medication implementation") |
| **#4** | (MH “Cardiovascular Diseases”) OR (“Cardiovascular Diseases” OR “Cardiovascular Disease” OR “Disease, Cardiovascular” OR “Diseases, Cardiovascular”) OR (MH “Heart Diseases”) OR (“Heart Diseases” OR “Disease, Heart” OR “Diseases, Heart” OR “Heart Disease” OR “Cardiac Diseases” OR “Cardiac Disease” OR “Disease, Cardiac” OR “Diseases, Cardiac”) OR (MH “Vascular Diseases”) OR (“Vascular Diseases” OR “Disease, Vascular” OR “Diseases, Vascular” OR “Vascular Disease”) OR (MH “Coronary Disease”) OR (“Coronary Disease” OR “Coronary Diseases” OR “Disease, Coronary” OR “Diseases, Coronary” OR “Coronary Heart Disease” OR “Coronary Heart Diseases” OR “Disease, Coronary Heart” OR “Diseases, Coronary Heart” OR “Heart Disease, Coronary” OR “Heart Diseases, Coronary”) OR (MH “Cerebrovascular Disorders”) OR (“Cerebrovascular Disorders” OR “Cerebrovascular Disorder” OR “Vascular Diseases, Intracranial” OR “Intracranial Vascular Disease” OR “Intracranial Vascular Diseases” OR “Vascular Disease, Intracranial” OR “Intracranial Vascular Disorders” OR “Intracranial Vascular Disorder” OR “Vascular Disorder, Intracranial” OR “Vascular Disorders, Intracranial” OR “Cerebrovascular Diseases” OR “Cerebrovascular Disease” OR “Disease, Cerebrovascular” OR “Diseases, Cerebrovascular” OR “Brain Vascular Disorders” OR “Brain Vascular Disorder” OR “Vascular Disorder, Brain” OR “Vascular Disorders, Brain” OR “Cerebrovascular Occlusion” OR “Cerebrovascular Occlusions” OR “Occlusion, Cerebrovascular” OR “Occlusions, Cerebrovascular” OR “Cerebrovascular Insufficiency” OR “Cerebrovascular Insufficiencies” OR “Insufficiencies, Cerebrovascular” OR “Insufficiency, Cerebrovascular”) OR (MH “Peripheral Vascular Diseases”) OR (“Peripheral Arterial Disease” OR “Arterial Disease, Peripheral” OR “Arterial Diseases, Peripheral” OR “Disease, Peripheral Arterial” OR “Diseases, Peripheral Arterial” OR “Peripheral Arterial Diseases” OR “Peripheral Artery Disease” OR “Artery Disease, Peripheral” OR “Artery Diseases, Peripheral” OR “Disease, Peripheral Artery” OR “Diseases, Peripheral Artery” OR “Peripheral Artery Diseases”) OR (MH “Rheumatic Heart Disease”) OR (“Rheumatic Heart Disease” OR “Disease, Rheumatic Heart” OR “Diseases, Rheumatic Heart” OR “Heart Disease, Rheumatic” OR “Heart Diseases, Rheumatic” OR “Rheumatic Heart Diseases” OR “Bouillaud Disease” OR “Disease, Bouillaud” OR “Bouillaud's Disease” OR “Bouillauds Disease” OR “Disease, Bouillaud's”) OR (MH “Venous Thrombosis”) OR (“Venous Thrombosis” OR Phlebothrombosis OR Phlebothromboses OR “Thrombosis, Venous” OR “Thromboses, Venous” OR “Venous Thromboses” OR “Deep Vein Thrombosis” OR “Deep Vein Thromboses” OR “Thromboses, Deep Vein” OR “Vein Thromboses, Deep” OR “Vein Thrombosis, Deep” OR “Deep-Venous Thrombosis” OR “Deep-Venous Thromboses” OR “Thromboses, Deep-Venous” OR “Thrombosis, Deep-Venous” OR “Deep-Vein Thrombosis” OR “Deep-Vein Thromboses” OR “Thromboses, Deep-Vein” OR “Thrombosis, Deep-Vein” OR “Thrombosis, Deep Vein” OR “Deep Venous Thrombosis” OR “Deep Venous Thromboses” OR “Thromboses, Deep Venous” OR “Thrombosis, Deep Venous” OR “Venous Thromboses, Deep” OR “Venous Thrombosis, Deep”) OR (MH “Pulmonary Embolism”) OR (“Pulmonary Embolism” OR “Pulmonary Embolisms” OR “Embolism, Pulmonary” OR “Embolisms, Pulmonary” OR “Pulmonary Thromboembolisms” OR “Pulmonary Thromboembolism” OR “Thromboembolism, Pulmonary” OR “Thromboembolisms, Pulmonary”) OR (MH Stroke) OR (Stroke OR Strokes OR “Cerebrovascular Accident” OR “Cerebrovascular Accidents” OR “CVA (Cerebrovascular Accident)” OR “CVAs (Cerebrovascular Accident)” OR “Cerebrovascular Apoplexy” OR “Apoplexy, Cerebrovascular” OR “Vascular Accident, Brain” OR “Brain Vascular Accident” OR “Brain Vascular Accidents” OR “Vascular Accidents, Brain” OR “Cerebrovascular Stroke” OR “Cerebrovascular Strokes” OR “Stroke, Cerebrovascular” OR “Strokes, Cerebrovascular” OR Apoplexy OR “Cerebral Stroke” OR “Cerebral Strokes” OR “Stroke, Cerebral” OR “Strokes, Cerebral” OR “Stroke, Acute” OR “Acute Stroke” OR “Acute Strokes” OR “Strokes, Acute” OR “Cerebrovascular Accident, Acute” OR “Acute Cerebrovascular Accident” OR “Acute Cerebrovascular Accidents” OR “Cerebrovascular Accidents, Acute”) OR (MH Hypertension) OR (Hypertension OR “Blood Pressure, High” OR “Blood Pressures, High” OR “High Blood Pressure” OR “High Blood Pressures”) OR (MH “Myocardial Infarction”) OR (“Myocardial Infarction” OR “Infarction, Myocardial” OR “Infarctions, Myocardial” OR “Myocardial Infarctions” OR “Cardiovascular Stroke” OR “Cardiovascular Strokes” OR “Stroke, Cardiovascular” OR “Strokes, Cardiovascular” OR “Heart Attack” OR “Heart Attacks” OR “Myocardial Infarct” OR “Infarct, Myocardial” OR “Infarcts, Myocardial” OR “Myocardial Infarcts”) OR (MH “Heart Failure”) OR (“Heart Failure” OR “Cardiac Failure” OR “Heart Decompensation” OR “Decompensation, Heart” OR “Heart Failure, Right-Sided” OR “Heart Failure, Right Sided” OR “Right-Sided Heart Failure” OR “Right Sided Heart Failure” OR “Myocardial Failure” OR “Congestive Heart Failure” OR “Heart Failure, Congestive” OR “Heart Failure, Left-Sided” OR “Heart Failure, Left Sided” OR “Left-Sided Heart Failure” OR “Left Sided Heart Failure”) OR (MH "Diabetes Mellitus, Type 2") OR ("Diabetes Mellitus, Type 2" OR "Diabetes Mellitus, Noninsulin-Dependent" OR “Diabetes Mellitus, Noninsulin-Dependent” OR “Diabetes Mellitus, Ketosis-Resistant” OR “Diabetes Mellitus, Ketosis Resistant” OR “Ketosis-Resistant Diabetes Mellitus” OR “Diabetes Mellitus, Non Insulin Dependent” OR “Diabetes Mellitus, Non-Insulin-Dependent” OR “Non-Insulin-Dependent Diabetes Mellitus” OR “Diabetes Mellitus, Stable” OR “Stable Diabetes Mellitus” OR “Diabetes Mellitus, Type II” OR NIDDM OR “Diabetes Mellitus, Noninsulin Dependent” OR “Diabetes Mellitus, Maturity-Onset” OR “Diabetes Mellitus, Maturity Onset” OR “Maturity-Onset Diabetes Mellitus” OR “Maturity Onset Diabetes Mellitus” OR MODY OR “Diabetes Mellitus, Slow-Onset” OR “Diabetes Mellitus, Slow Onset” OR “Slow-Onset Diabetes Mellitus” OR “Type 2 Diabetes Mellitus” OR “Noninsulin-Dependent Diabetes Mellitus” OR “Noninsulin Dependent Diabetes Mellitus” OR “Maturity-Onset Diabetes” OR “Diabetes, Maturity-Onset” OR “Maturity Onset Diabetes” OR “Type 2 Diabetes” OR “Diabetes, Type 2” OR “Diabetes Mellitus, Adult-Onset” OR “Adult-Onset Diabetes Mellitus” OR “Diabetes Mellitus, Adult Onset”) |
| **#5** | **#1 AND #2 AND #3 AND #4** |

**Table S8 – ProQuest syntax**

| **Search** | **Query** |
| --- | --- |
| **#1** | (instrumentation OR methods OR "Validation Studies” OR "Comparative Study" OR "psychometrics" OR psychometr* OR clinimetr* OR clinometr* OR "outcome assessment (health care)" OR "outcome assessment" OR "outcome measure*" OR "observer variation" OR "observer variation" OR "Health Status Indicators" OR "reproducibility of results" OR reproducib* OR "discriminant analysis" OR reliab* OR unreliab* OR valid* OR "coefficient of variation" OR coefficient OR homogeneity OR homogeneous OR "internal consistency" OR (cronbach* AND (alpha OR alphas)) OR (item AND (correlation* OR selection* OR reduction*)) OR agreement OR precision OR imprecision OR "precise values" OR test-retest OR (test AND retest) OR (reliab* AND (test OR retest)) OR stability OR interrater OR inter-rater OR intrarater OR intra-rater OR intertester OR inter-tester OR intratester OR intra-tester OR interobserver OR inter-observer OR intraobserver OR intra-observer OR intertechnician OR inter-technician OR intratechnician OR intra-technician OR interexaminer OR inter-examiner OR intraexaminer OR intra-examiner OR interassay OR inter-assay OR intraassay OR intra-assay OR interindividual OR inter-individual OR intraindividual OR intra-individual OR interparticipant OR inter-participant OR intraparticipant OR intra-participant OR kappa OR kappa's OR kappas OR repeatab* OR ((replicab* OR repeated) AND (measure OR measures OR findings OR result OR results OR test OR tests)) OR generaliza* OR generalisa* OR concordance OR (intraclass AND correlation*) OR discriminative OR "known group" OR "factor analysis" OR "factor analyses" OR "factor structure" OR "factor structures" OR dimension* OR subscale* OR (multitrait AND scaling AND (analysis OR analyses)) OR "item discriminant" OR "interscale correlation*" OR error OR errors OR "individual variability" OR "interval variability" OR "rate variability" OR (variability AND (analysis OR values)) OR (uncertainty AND (measurement OR measuring)) OR "standard error of measurement" OR sensitiv* OR responsive* OR (limit AND detection) OR "minimal detectable concentration" OR interpretab* OR ((minimal OR minimally OR clinical OR clinically) AND (important OR significant OR detectable) AND (change OR difference)) OR (small* AND (real OR detectable) AND (change OR difference)) OR "meaningful change" OR "ceiling effect" OR "floor effect" OR "Item response model" OR “IRT” OR Rasch OR "Differential item functioning" OR “DIF” OR "computer adaptive testing" OR "item bank" OR "cross-cultural equivalence") |
| **#2** | (MESH.EXACT("Surveys and Questionnaires") OR ("Surveys and Questionnaires" OR "Questionnaires and Surveys" OR "Survey Methods" OR "Methods, Survey" OR "Survey Method" OR "Methodology, Survey" OR "Survey Methodology" OR "Community Surveys" OR "Community Survey" OR "Survey, Community" OR "Surveys, Community" OR "Repeated Rounds of Survey" OR Surveys OR Survey OR "Questionnaire Design" OR "Design, Questionnaire" OR "Designs, Questionnaire" OR "Questionnaire Designs" OR "Baseline Survey" OR "Baseline Surveys" OR "Survey, Baseline" OR "Surveys, Baseline" OR Respondents OR Respondent OR "Randomized Response Technique" OR "Randomized Response Techniques" OR "Response Technique, Randomized" OR "Response Techniques, Randomized" OR "Techniques, Randomized Response" OR Questionnaires OR Questionnaire OR Nonrespondents OR Nonrespondent)) OR (MESH.EXACT("Patient Reported Outcome Measures") OR ("Patient Reported Outcome Measures" OR "Patient Reported Outcomes" OR "Outcome, Patient Reported" OR "Outcomes, Patient Reported" OR "Reported Outcome, Patient" OR "Reported Outcomes, Patient" OR "Patient Reported Outcome")) OR (MESH.EXACT("Self Report") OR ("Self Report" OR "Report, Self" OR "Reports, Self" OR "Self Reports")) OR (index OR indices OR "self-report" OR "self-report measures" OR “assessment tools” OR “patient-reported outcome” OR “measurement scale” OR “health measurement scale” OR “measure health outcomes” OR instrument OR "measurement instrument" OR scale OR measure OR tool) |
| **#3** | (MESH.EXACT("Medication Adherence") OR ("Medication Adherence" OR "Adherence, Medication" OR "Medication Nonadherence" OR "Nonadherence, Medication" OR "Medication Noncompliance" OR "Noncompliance, Medication" OR "Medication Non-Adherence" OR "Medication Non Adherence" OR "Non-Adherence, Medication" OR "Medication Persistence" OR "Persistence, Medication" OR "Medication Compliance" OR "Compliance, Medication" OR "Medication Non-Compliance" OR "Medication Non Compliance" OR "Non-Compliance, Medication")) OR (MESH.EXACT("Patient Compliance") OR ("Patient Compliance" OR "Compliance, Patient" OR "Patient Adherence" OR "Adherence, Patient" OR "Patient Cooperation" OR "Cooperation, Patient" OR "Patient Non-Compliance" OR "Non-Compliance, Patient" OR "Patient Non Compliance" OR "Patient Nonadherence" OR "Nonadherence, Patient" OR "Patient Noncompliance" OR "Noncompliance, Patient" OR "Patient Non-Adherence" OR "Non-Adherence, Patient" OR "Patient Non Adherence" OR "Treatment Compliance" OR "Compliance, Treatment" OR "Treatment Compliances" OR "Therapeutic Compliance" OR "Compliance, Therapeutic" OR "Compliances, Therapeutic" OR "Therapeutic Compliances")) OR ("drug adherence" OR "drug compliance" OR "taking medication" OR "medication initiation" OR "medication implementation") |
| **#4** | (MESH.EXACT("Cardiovascular Diseases") OR ("Cardiovascular Diseases" OR "Cardiovascular Disease" OR "Disease, Cardiovascular" OR "Diseases, Cardiovascular")) OR (MESH.EXACT("Heart Diseases") OR ("Heart Diseases" OR "Disease, Heart" OR "Diseases, Heart" OR "Heart Disease" OR "Cardiac Diseases" OR "Cardiac Disease" OR "Disease, Cardiac" OR "Diseases, Cardiac")) OR (MESH.EXACT("Vascular Diseases") OR ("Vascular Diseases" OR "Disease, Vascular" OR "Diseases, Vascular" OR "Vascular Disease")) OR (MESH.EXACT("Coronary Disease") OR ("Coronary Disease" OR "Coronary Diseases" OR "Disease, Coronary" OR "Diseases, Coronary" OR "Coronary Heart Disease" OR "Coronary Heart Diseases" OR "Disease, Coronary Heart" OR "Diseases, Coronary Heart" OR "Heart Disease, Coronary" OR "Heart Diseases, Coronary")) OR (MESH.EXACT("Cerebrovascular Disorders") OR ("Cerebrovascular Disorders" OR "Cerebrovascular Disorder" OR "Vascular Diseases, Intracranial" OR "Intracranial Vascular Disease" OR "Intracranial Vascular Diseases" OR "Vascular Disease, Intracranial" OR "Intracranial Vascular Disorders" OR "Intracranial Vascular Disorder" OR "Vascular Disorder, Intracranial" OR "Vascular Disorders, Intracranial" OR "Cerebrovascular Diseases" OR "Cerebrovascular Disease" OR "Disease, Cerebrovascular" OR "Diseases, Cerebrovascular" OR "Brain Vascular Disorders" OR "Brain Vascular Disorder" OR "Vascular Disorder, Brain" OR "Vascular Disorders, Brain" OR "Cerebrovascular Occlusion" OR "Cerebrovascular Occlusions" OR "Occlusion, Cerebrovascular" OR "Occlusions, Cerebrovascular" OR "Cerebrovascular Insufficiency" OR "Cerebrovascular Insufficiencies" OR "Insufficiencies, Cerebrovascular" OR "Insufficiency, Cerebrovascular")) OR (MESH.EXACT("Peripheral Arterial Disease") OR ("Peripheral Arterial Disease" OR "Arterial Disease, Peripheral" OR "Arterial Diseases, Peripheral" OR "Disease, Peripheral Arterial" OR "Diseases, Peripheral Arterial" OR "Peripheral Arterial Diseases" OR "Peripheral Artery Disease" OR "Artery Disease, Peripheral" OR "Artery Diseases, Peripheral" OR "Disease, Peripheral Artery" OR "Diseases, Peripheral Artery" OR "Peripheral Artery Diseases")) OR (MESH.EXACT("Rheumatic Heart Disease") OR ("Rheumatic Heart Disease" OR "Disease, Rheumatic Heart" OR "Diseases, Rheumatic Heart" OR "Heart Disease, Rheumatic" OR "Heart Diseases, Rheumatic" OR "Rheumatic Heart Diseases" OR "Bouillaud Disease" OR "Disease, Bouillaud" OR "Bouillaud's Disease" OR "Bouillauds Disease" OR "Disease, Bouillaud's")) OR (MESH.EXACT("Venous Thrombosis") OR ("Venous Thrombosis" OR Phlebothrombosis OR Phlebothromboses OR "Thrombosis, Venous" OR "Thromboses, Venous" OR "Venous Thromboses" OR "Deep Vein Thrombosis" OR "Deep Vein Thromboses" OR "Thromboses, Deep Vein" OR "Vein Thromboses, Deep" OR "Vein Thrombosis, Deep" OR "Deep-Venous Thrombosis" OR "Deep-Venous Thromboses" OR "Thromboses, Deep-Venous" OR "Thrombosis, Deep-Venous" OR "Deep-Vein Thrombosis" OR "Deep-Vein Thromboses" OR "Thromboses, Deep-Vein" OR "Thrombosis, Deep-Vein" OR "Thrombosis, Deep Vein" OR "Deep Venous Thrombosis" OR "Deep Venous Thromboses" OR "Thromboses, Deep Venous" OR "Thrombosis, Deep Venous" OR "Venous Thromboses, Deep" OR "Venous Thrombosis, Deep")) OR (MESH.EXACT("Pulmonary Embolism") OR ("Pulmonary Embolism" OR "Pulmonary Embolisms" OR "Embolism, Pulmonary" OR "Embolisms, Pulmonary" OR "Pulmonary Thromboembolisms" OR "Pulmonary Thromboembolism" OR "Thromboembolism, Pulmonary" OR "Thromboembolisms, Pulmonary")) OR (MESH.EXACT("Stroke") OR (Stroke OR Strokes OR "Cerebrovascular Accident" OR "Cerebrovascular Accidents" OR "CVA (Cerebrovascular Accident)" OR "CVAs (Cerebrovascular Accident)" OR "Cerebrovascular Apoplexy" OR "Apoplexy, Cerebrovascular" OR "Vascular Accident, Brain" OR "Brain Vascular Accident" OR "Brain Vascular Accidents" OR "Vascular Accidents, Brain" OR "Cerebrovascular Stroke" OR "Cerebrovascular Strokes" OR "Stroke, Cerebrovascular" OR "Strokes, Cerebrovascular" OR Apoplexy OR "Cerebral Stroke" OR "Cerebral Strokes" OR "Stroke, Cerebral" OR "Strokes, Cerebral" OR "Stroke, Acute" OR "Acute Stroke" OR "Acute Strokes" OR "Strokes, Acute" OR "Cerebrovascular Accident, Acute" OR "Acute Cerebrovascular Accident" OR "Acute Cerebrovascular Accidents" OR "Cerebrovascular Accidents, Acute")) OR (MESH.EXACT("Hypertension") OR (Hypertension OR "Blood Pressure, High" OR "Blood Pressures, High" OR "High Blood Pressure" OR "High Blood Pressures")) OR (MESH.EXACT("Myocardial Infarction") OR ("Myocardial Infarction" OR "Infarction, Myocardial" OR "Infarctions, Myocardial" OR "Myocardial Infarctions" OR "Cardiovascular Stroke" OR "Cardiovascular Strokes" OR "Stroke, Cardiovascular" OR "Strokes, Cardiovascular" OR "Heart Attack" OR "Heart Attacks" OR "Myocardial Infarct" OR "Infarct, Myocardial" OR "Infarcts, Myocardial" OR "Myocardial Infarcts")) OR (MESH.EXACT("Heart Failure") OR ("Heart Failure" OR "Cardiac Failure" OR "Heart Decompensation" OR "Decompensation, Heart" OR "Heart Failure, Right-Sided" OR "Heart Failure, Right Sided" OR "Right-Sided Heart Failure" OR "Right Sided Heart Failure" OR "Myocardial Failure" OR "Congestive Heart Failure" OR "Heart Failure, Congestive" OR "Heart Failure, Left-Sided" OR "Heart Failure, Left Sided" OR "Left-Sided Heart Failure" OR "Left Sided Heart Failure")) OR (MESH.EXACT("Diabetes Mellitus, Type 2") OR ("Diabetes Mellitus, Type 2" OR "Diabetes Mellitus, Noninsulin-Dependent" OR "Diabetes Mellitus, Noninsulin-Dependent" OR "Diabetes Mellitus, Ketosis-Resistant" OR "Diabetes Mellitus, Ketosis Resistant" OR "Ketosis-Resistant Diabetes Mellitus" OR "Diabetes Mellitus, Non Insulin Dependent" OR "Diabetes Mellitus, Non-Insulin-Dependent" OR "Non-Insulin-Dependent Diabetes Mellitus" OR "Diabetes Mellitus, Stable" OR "Stable Diabetes Mellitus" OR "Diabetes Mellitus, Type II" OR NIDDM OR "Diabetes Mellitus, Noninsulin Dependent" OR "Diabetes Mellitus, Maturity-Onset" OR "Diabetes Mellitus, Maturity Onset" OR "Maturity-Onset Diabetes Mellitus" OR "Maturity Onset Diabetes Mellitus" OR MODY OR "Diabetes Mellitus, Slow-Onset" OR "Diabetes Mellitus, Slow Onset" OR "Slow-Onset Diabetes Mellitus" OR "Type 2 Diabetes Mellitus" OR "Noninsulin-Dependent Diabetes Mellitus" OR "Noninsulin Dependent Diabetes Mellitus" OR "Maturity-Onset Diabetes" OR "Diabetes, Maturity-Onset" OR "Maturity Onset Diabetes" OR "Type 2 Diabetes" OR "Diabetes, Type 2" OR "Diabetes Mellitus, Adult-Onset" OR "Adult-Onset Diabetes Mellitus" OR "Diabetes Mellitus, Adult Onset")) |
| **#5** | **#1 AND #2 AND #3 AND #4** |
