# Supplementary material for: Quality of measurement properties of medication adherence instruments in cardiovascular diseases and type 2 diabetes mellitus: a systematic review and meta-analysis
Source: Syst Rev. 2023 Nov 22;12:222. doi: 10.1186/s13643-023-02340-z (PMC10664314; doi:10.1186/s13643-023-02340-z)
Supplement: Supplementary file 3 — Additional file 3. Studies’ characteristics. [file 13643_2023_2340_MOESM3_ESM.docx]

Additional file 3. Studies’ characteristics.

| **PROM** | **Reference** | **Population** | | | **Disease characteristics** | | | | **Instrument administration** | | | **RR** | **CI** | **Funding** |
| --- | --- | --- | --- | --- | --- | --- | --- | --- | --- | --- | --- | --- | --- | --- |
|  |  | **n** | **Age** | **Gender** | | **Disease** | **DD** | **NMU** | **Setting** | **Country** | **Language** |  |  |  |
|  |  |  | **Mean (SD)** | **% female** | |  | **Mean (SD)** | **Mean (SD)** |  |  |  |  |  |  |
| MMAS-8 | 28 | 62 | 47.0 (4.7) | 53.2% | | T2DM | 12.1 (8.6) | NI | Hospital | Malaysia | Malay | NI | No | No |
|  | 67 | 70 | 65.7 (9.9) | 31.4% | | CVD | NI | 5.2 (2.3) | Ambulatory | Switzerland | German | NI | NI | NI |
|  | 30 | 103 | 52.7 (8.6) | 68.9% | | T2DM | NI | NI | Ambulatory | Libya | Arabic | 82.4% | No | No |
|  | 68 | 196 | 61.8 (11.4) | 60.7% | | CVD | 8.6 (6.4) | 1.6 (0.8) | Ambulatory | Turkey | Turkish | NI | No | NI |
|  | 73 | 472 | 68.2 (10.6) | 51.5% | | CVD | 10.9 (8.5) | NI | Community pharmacies and Family Hospital | Portugal | Portuguese | NI | No | NI |
|  | 76 | 937 | 57.1 (12.7) | 71.5% | | CVD | NI | 1.6 (0.7) | Health Units | Brazil | Portuguese | NI | NI | Yes |
|  | 35 | 1198 | 60.7 (10.7) | 38.6% | | T2DM | 15.8 (9.3) | NI | US National Health and Wellness Survey | United States | English | NI | No | Yes |
|  | 79 | 200 | 59.7 (27.2) | 58.0% | | CVD | 7.2 (5.7) | NI | Hostipal, office and pharmacy | Iran | Persian | NI | No | No |
|  | 80 | 1367 | 52.5 (12.2) | 59.2% | | CVD | NI | NI | Hospital | United States | English | 98.0% | No | Yes |
|  | 82 | 210 | 78.1 (5.8) | 68.1% | | CVD | NI | NI | Managed care organization | United States | English | NI | No | Yes |
|  | 39 | 144 | 50.7 (11.9) | 61.1% | | T2DM | 10.0 (7.6) | NI | Health Center | United States | English | NI | No | No |
|  | 84 | 47 | 52.6 (11.0) | 47.0% | | CVD | NI | NI | Hospital | United States | English | 94.0% | No | Yes |
|  | 121 | 100 | 65.5 (10.8) | 57.0% | | CVD, DM | NI | 5.3 (3.4) | Community pharmacy | Spain | Spanish | NI | NI | NI |
|  | 40 | 901 | NI | 41.6% | | T2DM | NI | NI | Advocacy association | Canada | French | NI | No | Yes |
|  | 41 | 175 | 60.6 (9.2) | 53.7% | | T2DM | 10.5 (7.5) | 4.3 (1.7) | Hospital | Malaysia | Malay | NI | No | Yes |
|  | 86 | 110 | 60.7 (2.6) | 54.6% | | CVD | 9.2 (7.4) | 3.6 (4.3) | Family doctor practice | Poland | Polish | 73.1% | Yes | NI |
|  | 43 | 430 | 58.7 (11.6) | 54.9% | | T2DM | NI | NI | Home visit | United States | English | NI | No | Yes |
|  | 87 | 373 | 57.2 (11.2) | 45.0% | | CVD | 4.4 (4.3) | 2.2 (1.0) | Hospital | South Korea | Korean | 93.3% | No | Yes |
|  | 88 | 199 | 55.7 (14.6) | 42.7% | | CVD | NI | NI | Hospital | France | French | NI | Yes | Yes |
|  | 90 | 87 | 76.0 (NI) | 69.0% | | CVD | NI | 2.2 (NI) | Managed care organization | United States | English | 65.5% | NI | Yes |
|  | 46 | 317 | 59.3 (11.2) | 38.5% | | T2DM | 4.2 (4.1) | 2.0 (0.9) | Hospital | South Korea | Korean | 91.7% | No | Yes |
|  | 91 | 200 | 70.6 (10.3) | 50.0% | | CVD | NI | NI | Outpatient clinic | United States | Mandarin | NI | NI | Yes |
|  | 93 | 329 | NI | 69.0% | | CVD | NI | NI | Tertiary care hypertension clinic | Uganda | Runyankore/Rukiga | 99.4% | No | Yes |
|  | 94 | 99 | 63.2 (8.8) | 17.0% | | CVD | NI | NI | Hospital | Norway | NI | 83.0% | No | Yes |
|  | 47 | 303 | 61.1 (11.4) | 71.0% | | T2DM | NI | 2.7 (1.2) | Hospital | Thailand | Thai | NI | NI | Yes |
|  | 99 | 101 | 55.1 (17.5) | 59.4% | | CVD | NI | NI | Hospital | Saudi Arabia | Arabic | 85.7% | No | No |
|  | 100 | 92 | 73.2 (6.5) | 79.3% | | CVD | NI | NI | Primary health care posts | South Korea | Korean | NI | NI | Yes |
|  | 124 | 180 | 59.1 (11.0) | 57.8% | | CVD, DM | NI | NI | Primary Health Centre | India | Kannada | NI | No | No |
|  | 48 | 154 | 57.5 (10.0) | 73.0% | | T2DM | 6.5 (6.0) | 1.3 (NI) | Clinic | Togo | French and Ewe | NI | No | Yes |
|  | 101 | 330 | NI | NI | | CVD | NI | NI | Primary care center | Chile | Spanish | NI | No | Yes |
|  | 50 | 182 | 64.9 (9.6) | 72.5% | | T2DM | 8.3 (6.4) | NI | Hospital | China | Chinese | NI | NI | NI |
|  | 103 | 174 | 58.7 (15.4) | 50.0% | | CVD | NI | NI | Hospital | Singapore | English and Chinese | 86.1% | No | NI |
|  | 104 | 176 | 64.6 (12.0) | 24.4% | | CVD | NI | NI | Hospital | China | Mandarin | 97.8% | NI | Yes |
|  | 53 | 153 | NI | 37.9% | | T2DM | NI | NI | Web survey | Canada | French | 70.6% | NI | Yes |
|  | 55 | 589 | 56.4 (11.9) | 67.9% | | T2DM | NI | NI | Health care centers | Iran | Persian | 98.1% | No | No |
|  | 57 | 199 | 55.0 (13.1) | 60.3% | | T2DM | NI | NI | Hospital | Turkey | Turkish | 99.5% | Yes | No |
|  | 108 | 151 | 56.0 (14.4) | 47.7% | | CVD | NI | NI | Hospital | Singapore | English and Chinese | 82.1% | No | NI |
|  | 58 | 307 | 7.0 (1.8) | 66.1% | | T2DM | NI | NI | Hospital | Iraq | Kurdish | NI | NI | NI |
|  | 59 | 92 | NI | 51.0% | | T2DM | NI | NI | Primary Health Facility | Indonesia | Indonesian | NI | No | Yes |
|  | 60 | 138 | 54.5 (13.5) | 59.4% | | DM | 10.9 (7.38) | NI | Hospital | Iran | Persian | 92.0% | No | No |
|  | 61 | 320 | 58.0 (13.7) | 63.8% | | T2DM | NI | NI | Family Medicine Clinics | Iran | Persian | NI | No | No |
|  | 62 | 232 | 65.4 (NI) | 41.8% | | T2DM | NI | NI | Hospital | Spain | Spanish | NI | No | No |
| SMAQ | 29 | 31 | 57.9 (11.8) | 54.8% | | DM | NI | NI | Hospital | United States | Amharic | NI | NI | Yes |
|  |  | 35 | NI | 37.1% | | DM | NI | NI | Clinics | United States | Arabic | NI | NI | NI |
| MEDS | 114 | 685 | NI | 56.5% | | CVD, DM | NI | NI | Community pharmacies | United States | English | 21.1% | No | Yes |
| MNPS | 115 | 675 | NI | 56.9% | | CVD, DM | NI | NI | Community pharmacies | United States | English | 21.1% | NI | NI |
| DMAS-7 | 31 | 500 | 59.2 (10.7) | 59.8% | | T2DM | 8.6 (7.6) | NI | Hospital and clinics | Lebanon | Arabic | NI | No | Yes |
|  | 36 | 300 | 58.0 (11.7) | 57.0% | | T2DM | NI | 5.2 (2.7) | Hospital and clinics | Lebanon | Arabic | NI | No | Yes |
| ARMS-12 | 116 | 53 | 63.0 (10.5) | 52.8% | | CVD, T2DM | NI | 9.0 (3.0) | Hospital | Brazil | Portuguese | NI | No | Yes |
|  | 44 | 304 | 57.4 (12.3) | 61.2% | | T2DM | NI | NI | Hospital | South Korea | Korean | 89.1% | NI | Yes |
|  | 123 | 435 | 63.7 (10.3) | 55.6% | | CVD, DM | NI | NI | Primary care clinic | United States | English | NI | NI | Yes |
|  | 106 | 279 | 66.5 (11.0) | 66.5% | | CVD | NI | NI | Hospital | Poland | Polish | 96.2% | No | NI |
|  | 64 | 505 | 73.0 (NI) | 54.5% | | T2DM | 15.0 (NI) | 2.0 (NI) | Hospital | China | Chinese | NI | NI | NI |
|  | 132 | 202 | NI | 30.1% | | CVD, DM | NI | NI | Hospital | Saudi Arabia | Arabic | 76.5% | No | Yes |
| MGT | 69 | 206 | 66.6 (13.2) | 64.6% | | CVD | NI | NI | Ambulatory | Brazil | Portuguese | NI | No | Yes |
|  | 70 | 200 | 63.0 (10.3) | 73.5% | | CVD | NI | 3.9 (0.9) | Clinic | Brazil | Portuguese | NI | NI | NI |
|  | 118 | 139 | 63.0 (NI) | 71.9% | | CVD, DM | NI | NI | Primary care physician office sites | United States | English | NI | No | NI |
|  | 78 | 90 | 59.2 (11.0) | 72.0% | | CVD | NI | NI | Health center | Spain | Spanish | NI | NI | NI |
|  | 81 | 290 | NI | 70.0% | | CVD | 6.0 (NI) | NI | Hospital | United States | English | NI | NI | Yes |
|  | 83 | 437 | 62.5 (8.9) | 70.0% | | CVD | NI | NI | Not clear | United States | English | 46.4% | No | No |
|  | 85 | 157 | NI | NI | | CVD | NI | NI | Health Center | Spain | Spanish | NI | NI | NI |
|  | 122 | 415/353 | 60.3 (11.3) | 77.3% | | CVD, DM | NI | 6.0 (4.2) | Primary care network | United States | English | NI | NI | Yes |
|  | 89 | 353 | 64.0 (11.0) | 49.0% | | CVD | NI | NI | Primare care | Germany | German | NI | NI | NI |
|  | 95 | 174 | 64.1 (10.7) | 66.7% | | CVD | NI | NI | Health Center | Spain | Spanish | NI | NI | NI |
|  | 96 | 109 | NI | 71.6% | | CVD | NI | NI | Primary care unit | Brazil | Portuguese | 91.8% | NI | NI |
|  | 102 | 128 | 71.1 (NI) | 52.0% | | CVD | NI | NI | Primary care | Germany | German | NI | NI | NI |
|  | 51 | 294 | 58.0 (9.0) | 47.6% | | T2DM | NI | NI | Clinics | Singapore | English and Chinese | 81.4% | No | NI |
|  | 52 | 182 | 60.4 (10.8) | 63.7% | | T2DM | 7.8 (6.1) | NI | Hospital | Turkey | Turkish | NI | NI | NI |
|  | 53 | 153 | NI | 37.9% | | T2DM | NI | NI | Web survey | Canada | French | 70.6% | NI | Yes |
|  | 125 | 6261 | 76.0 (5.3) | 59.0% | | CVD, DM | NI | 9.4 (4.9) | Clinics | United States | English | NI | NI | Yes |
|  | 54 | 250 | 60.4 (8.9) | 63.2% | | T2DM | NI | NI | Primary health centers | Indonesia | Indonesian | NI | No | No |
|  | 56 | 107 | 64.9 (9.1) | 53.3% | | T2DM | NI | NI | Primare care centers | Spain | Spanish | NI | NI | NI |
|  | 107 | 86 | 64.4 (11.9) | 69.8% | | CVD | NI | NI | Primary care | Spain | Spanish | NI | NI | NI |
|  | 110 | 109 | 64.0 (NI) | 66.0% | | CVD | 5.5 | NI | Family medicine unit | Canada | French | 92.0% | NI | Yes |
| MTA-OA | 32 | 90 | 60.0 (8.1) | 67.8% | | T2DM | 17.3 (8.0) | NI | Hospital | Brazil | Portuguese | NI | NI | Yes |
| MTA - Insulin | 32 | 90 | 60.0 (8.1) | 67.8% | | T2DM | 17.3 (8) | NI | Hospital | Brazil | Portuguese | NI | NI | Yes |
| LMAS-14 | 71 | 405 | 65.1 (12.9) | 51.2% | | CVD | 9.5 (7.1) | 5.2 (3.0) | Hospital | Lebanon | Arabic | NI | No | Yes |
|  | 111 | 172 | 62.7 (13.4) | 38.4% | | CVD | NI | 6.2 (3.1) | Community pharmacies | Lebanon | Arabic | NI | No | Yes |
| MTA | 72 | 425 | 68.2 (10.6) | 53.2% | | CVD | NI | NI | Community pharmacies | Portugal | Portuguese | NI | NI | NI |
|  | 120 | 99 | 58.6 (14.0) | 44.4% | | CVD, DM | 5.5 (5.3) | 5.0 (2.2) | Hospital | Brazil | Portuguese | NI | No | Yes |
|  | 42 | 437 | NI | 70.3% | | T2DM | NI | NI | Health care units | Brazil | Portuguese | NI | No | Yes |
|  | 105 | 178 | 55.6 (NI) | 65.3% | | CVD | NI | 4;0 (NI) | Hospital | Brazil | Portuguese | NI | NI | NI |
|  | 126 | 167 | 65.7 (11.0) | 62.9% | | CVD, T2DM | NI | NI | Not clear | Portugal | Portuguese | NI | NI | NI |
| MARS-5 | 117 | Hypertension A = 50  Hypertension B = 178  Diabetes = 100  Asthma = 100  Total = 428 | Hypertension A = 62.3 (13.4)  Hypertension B = 53.6 (14.6)  Diabetes = 58.2 (15.9) | Hypertension A = 38%  Hypertension B = 48%  Diabetes = 32% | | CVD, DM | NI | NI | Hospital and Community clinics | England | English | Hypertension A = 86.0%, Hypertension B = 71.0%, Diabetes = 100.0% | Yes | NI |
|  | 77 | Study 1 = 241  Study 2 = 282  Total = 523 | Study 1 = 68.9 (12.1)  Study 2 = 64.7 (8.9) | Study 1 = 46.1%  Study 2 = 59.1% | | CVD | NI | NI | Hospital | Germany | German | 52.7% | NI | Yes |
|  | 92 | 523 | 72.9 (6.5) | 57.9% | | CVD | NI | NI | Hospital | Iran | Persian | NI | No | No |
|  | 102 | 128 | 71.1 (NI) | 52.0% | | CVD | NI | NI | Primary care | Germany | German | NI | NI | NI |
|  | 133 | 485 | 57.1 (12.8) | 49.3% | | CVD, DM | NI | 3.5 (1.9) | Tertiary care clinics | Jordan | Arabic | NI | No | Yes |
| A-14 | 74 | Hypertensive = 34  Non-hypertensive = 34  Total = 68 | Hypertensive = 65.4 (12.5)  Non-hypertensive = 65.5 (12.6) | 44.0% | | CVD | NI | NI | Hospital | Greece | Greek | NI | No | NI |
|  | 131 | 150 | NI | 41.0% | | CVD | NI | 7.0 (NI) | Hospital | Germany | German | 99.3% | No | Yes |
| ARMS-10 | 75 | 120 | 81.9 (5.2) | 55.8% | | CVD | NI | NI | Aged-care facility | China | Chinese | NI | No | NI |
| MALMAS | 33 | 43 | 61.9 (9.1) | 44.2% | | T2DM | 15.8 (9.4) | NI | Hospital | Malaysia | English | NI | NI | Yes |
|  | 34 | 136 | 58.1 (10.2) | 53.7% | | T2DM | 15.6 (8.3) | 7.5 (3.0) | Hospital | Malaysia | English | NI | No | Yes |
|  | 45 | 100 | NI | 48.0% | | T2DM | NI | NI | Hospital | Malaysia | Malay | 96.2% | No | Yes |
| ARMS-D | 37 | 314 | 51.8 (11.7) | 65.0% | | T2DM | 7.7 (6.7) | 1.6 (0.7) | Hospital | United States | English | 89.0% | No | Yes |
| IADMAS | 38 | 80 | 55.3 (9.0) | 40.0% | | T2DM | 9.3 (7.0) | 1.6 (0.7) | Hospital | Iraq | Arabic | 95.2% | No | No |
| GMAS | 119 | 171 | 51.0 (16.7) | 49.7% | | CVD, DM | NI | NI | Hospital | Saudi Arabia | English | 85.5% | No | No |
|  | 128 | 161 | 54.1 (1.1) | 40.4% | | CVD, DM | NI | NI | Hospital | Pakistan | Urdu | 91.0% | No | No |
|  | 129 | 196 | NI | 52.6% | | CVD, DM | NI | NI | Hospital | Pakistan | English | 91.6% | No | No |
|  | 134 | 282 | 43.1 (5.8) | 55.3% | | CVD, DM | NI | NI | Hospital | Saudi Arabia | Arabic | NI | No | No |
|  | 65 | 500 | NI | 71.0% | | T2DM | NI | NI | Health Center | Sudan | Arabic | NI | No | No |
|  | 135 | 256 | 59.6 (14.0) | 47.3% | | CVD, DM | NI | NI | Hospital and Community health service center | China | Chinese | NI | No | Yes |
|  | 136 | 177 | 59.4 (8.7) | 67.8% | | CVD, T2DM | NI | NI | Hospital | Vietnam | Vietnamese | 83.49% | No | No |
| MAQ | 97 | 299 | 59.7 (14.2) | 39.46% | | CVD | NI | NI | Hospital | India | Kannada, Malayalam | NI | No | NI |
| MMAS-5 | 98 | 159 | 62.0 (12.0) | 64.0% | | CVD | NI | NI | Primary care clinics | United States | English | NI | No | Yes |
| ProMAS | 49 | 304 | 60.8 (6.8) | 33.0% | | T2DM | 12.3 (7.7) | NI | Hospital | Netherlands | Dutch | 72.0% | No | Yes |
|  | 130 | 370 | 68.0 (7.1) | 38.11% | | CVD, DM | NI | NI | Web survey | Netherlands | Dutch | NI | No | NI |
| ARMS‐7 | 127 | 100 | 63.2 (9.5) | 61.0% | | CVD, DM | NI | 4.7 (2.2) | Hospital | Turkey | Turkish | NI | NI | NI |
| 5-item questionnaire | 109 | 806 | 59.0 (10.0) | 71.0% | | CVD | NI | NI | Ambulatory | United States | English | NI | NI | Yes |
| 3-item questionnaire | 137 | 459 | 64.3 (9.8) | 42.5% | | CVD, DM | NI | 8.1 (5.4) | Pragmatic trial | United States | English | 43.5% | NI | Yes |
| AS | 112 | PRIORITY study = 298; ANTEY study = 201 | PRIORITY: 62.5 (9.2); ANTEY: 71.1 (8.7) | PRIORITY: 48.0%; ANTEY: 41.3% | | CVD | NI | NI | Observational studies | Russia | Russian | NI | No | Yes |
| 12-item questionnaire | 66 | 30 | 51.4 (10.5) | 60.0% | | T2DM | NI | NI | Hospital | india | English | NI | No | No |
| Mascard | 113 | 219 | 65.0 (NI) | NI | | CVD | NI | 8 | Hospital | France | French | NI | No | NI |

Note: ARMS = Adherence to Refills and Medication Scale; AS = Adherence Scale; CI = Conflicts of interest; CVD = Cardiovascular diseases; DD = Disease duration; DM = Diabetes mellitus; DMAS-7 = 7-item Diabetes Medication Adherence Scale; GMAS = General Medication Adherence Scale; IADMAS = Iraqi Anti-Diabetic Medication Adherence Scale; LMAS-14 = Fourteen-item Lebanese Medication Adherence Scale; MALMAS = Malaysian Medication Adherence Scale; MAQ = Medication Adherence Questionnaire; MARS-5 = 5-item Medication Adherence Report Scale; Mascard = Medication Adherence Scale in Cardiovascular disorders; MEDS = Medication Adherence Estimation and Differentiation Scale; MGT = Morisky-Green test; MMAS-5 = 5-item adapted Morisky Medication Adherence Scale; MMAS-8 = 8-item Morisky Medication Adherence Scale; MNPS = Medication Non-persistence Scale; MTA = Measurement of Treatment Adherence; MTA-Insulin = Measurement of Treatment Adherence - Insulin; MTA-OA = MTA-Oral Antidiabetics; NI = Not informed; NMU = number of medications in use; PROM = Patient-reported outcome measures; ProMAS = Probabilistic Medication Adherence Scale; RR = Response rate; SD = Standard deviation; SMAQ = Simplified Medication Adherence Questionnaire; T2DM = Type 2 diabetes mellitus.
