# Supplementary material for: Quality of measurement properties of medication adherence instruments in cardiovascular diseases and type 2 diabetes mellitus: a systematic review and meta-analysis
Source: Syst Rev. 2023 Nov 22;12:222. doi: 10.1186/s13643-023-02340-z (PMC10664314; doi:10.1186/s13643-023-02340-z)
Supplement: Supplementary file 4 — Additional file 4. Quality of studies on the PROM development and content validity. [file 13643_2023_2340_MOESM4_ESM.docx]

Additional file 4. Quality of studies on the PROM development and content validity.

| **PROM** | **Reference** | **PROM development** | | | | | | **Content validity** | | | | |
| --- | --- | --- | --- | --- | --- | --- | --- | --- | --- | --- | --- | --- |
|  |  |  |  |  |  |  |  | **Asking patients** | | | **Asking experts** | |
|  |  | **Concept elicitation** | **Total PROM design** | **Comprehensibility** | **Comprehensiveness** | **Total cognitive interview or pilot study** | **Total PROM development** | **Relevance** | **Comprehensiveness** | **Comprehensibility** | **Relevance** | **Comprehensiveness** |
| MMAS-8 | 28 | - | - | - | - | - | - | - | - | D | - | - |
|  | 67 | - | - | - | - | - | - | D | D | D | - | - |
|  | 68 | - | - | - | - | - | - | - | - | D | - | - |
|  | 73 | - | - | - | - | - | - | - | - | D | - | - |
|  | 76 | - | - | - | - | - | - | - | - | D | - | - |
|  | 41 | - | - | - | - | - | - | - | - | D | - | - |
|  | 86 | - | - | - | - | - | - | - | - | D | - | - |
|  | 88 | - | - | - | - | - | - | - | - | D | - | - |
|  | 91 | - | - | - | - | - | - | - | - | D | - | - |
|  | 93 | - | - | - | - | - | - | - | - | D | - | - |
|  | 47 | - | - | - | - | - | - | - | - | D | - | - |
|  | 100 | - | - | - | - | - | - | - | - | D | - | - |
|  | 104 | - | - | - | - | - | - | - | - | D | - | - |
|  | 57 | - | - | - | - | - | - | - | - | D | - | - |
|  | 108 | - | - | - | - | - | - | - | - | D | - | - |
|  | 61 | - | - | - | - | - | - | - | - | - | D | D |
| MEDS | 114 | - | I | I | I | I | I | I | I | I | - | D |
| MTA - OA | 32 | - | - | - | - | - | - | - | D | D | D | D |
| MTA - Insulin | 32 | - | - | - | - | - | - | - | D | D | D | D |
| A-14 | 131 | - | I | - | - | I | I | - | - | - | - | - |
| MARS-5 | 77 | - | - | - | - | - | - | - | - | D | - | - |
| ARMS-12 | 123 | - | I | D | D | D | I | - | - | - | - | - |
|  | 44 | - | - | - | - | - | - | - | - | D | - | - |
|  | 106 | - | - | - | - | - | - | - | - | D | - | - |
|  | 132 | - | - | - | - | - | - | - | - | D | - | - |
| MGT | 52 | - | - | - | - | - | - | D | - | - | D | D |
| MTA | 105 | - | - | - | - | - | - | - | D | D | D | - |
| ARMS-7 | 127 | - | - | - | - | - | - | - | - | D | - | - |
| IADMAS | 38 | - | I | D | D | D | I | - | - | - | - | - |
| GMAS | 128 | - | I | D | D | D | I | - | - | D | D | - |
|  | 129 | - | - | - | - | - | - | - | - | D | - | - |
|  | 134 | - | - | - | - | - | - | - | - | D | - | - |
|  | 136 | - | - | - | - | - | - | - | - | D | - | - |
| ProMAS | 130 | D | D | D | D | D | D | - | - | - | - | - |
| 12-item questionnaire | 66 | - | I | - | - | I | I | - | - | - | D | D |
| Mascard | 113 | D | D | D | D | D | D | - | - | D | D | D |

Note: ARMS = Adherence to Refills and Medication Scale; D = Doubtful; GMAS = General Medication Adherence Scale; I = Inadequate; IADMAS = Iraqi Anti-Diabetic Medication Adherence Scale; MALMAS = Malaysian Medication Adherence Scale; MARS-5 = 5-item Medication Adherence Report Scale; Mascard = Medication Adherence Scale in Cardiovascular disorders; MEDS = Medication Adherence Estimation and Differentiation Scale; MGT = Morisky-Green test; MMAS-8 = 8-item Morisky Medication Adherence Scale; MTA = Measurement of Treatment Adherence; MTA-Insulin = Measurement of Treatment Adherence - Insulin; MTA-OA = MTA-Oral Antidiabetics; PROM = Patient-reported outcome measures; ProMAS = Probabilistic Medication Adherence Scale; - = Not evaluated.
