# Supplementary material for: Quality of measurement properties of medication adherence instruments in cardiovascular diseases and type 2 diabetes mellitus: a systematic review and meta-analysis
Source: Syst Rev. 2023 Nov 22;12:222. doi: 10.1186/s13643-023-02340-z (PMC10664314; doi:10.1186/s13643-023-02340-z)
Supplement: Supplementary file 5 — Additional file 5. Quality of studies on measurement properties. [file 13643_2023_2340_MOESM5_ESM.docx]

Additional file 5. Quality of studies on measurement properties.

| **PROM** | **Reference** | **Structural validity** | **Internal consistency** | **Reliability** | **Criterion validity** | **Construct validity** | | **Responsiveness** |
| --- | --- | --- | --- | --- | --- | --- | --- | --- |
|  |  |  |  |  |  | **Convergent validity** | **Known groups validity** | **Comparison before and after intervention** |
| MMAS-8 | 28 | D | V | - | - | - | - | - |
|  | 67 | A | V | - | V | V | - | - |
|  | 30 | - | V | - | V | - | - | - |
|  | 68 | A | V | - | - | - | - | - |
|  | 73 | V | V | - | - | V | V | - |
|  | 76 | - | V | D | V | - | - | - |
|  | 35 | A | V | - | - | - | - | - |
|  | 79 | A | V | D | V | - | - | - |
|  | 80 | V | V | - | V | V | - | - |
|  | 82 | - | - | D | - | - | - | - |
|  | 39 | D | V | - | V | V | - | - |
|  | 84 | - | V | - | V | - | - | - |
|  | 121 | A | V | - | I | - | - | - |
|  | 40 | A | V | - | - | - | - | - |
|  | 41 | - | V | D | V | V | - | - |
|  | 86 | D | V | I | - | - | A | - |
|  | 43 | - | - | - | I | - | - | - |
|  | 87 | V | V | I | V | V | V | - |
|  | 88 | V | V | I | - | - | - | - |
|  | 90 | - | - | - | V | - | - | - |
|  | 46 | V | V | I | V | V | - | - |
|  | 91 | - | D | - | - | - | - | - |
|  | 93 | A | V | I | - | - | - | - |
|  | 94 | - | V | D | - | - | - | - |
|  | 47 | A | V | I | V | V | - | - |
|  | 99 | - | V | - | V | V | V | - |
|  | 100 | A | V | - | V | - | V | - |
|  | 124 | - | V | - | V | - | - | - |
|  | 48 | - | V | - | V | V | - | - |
|  | 101 | - | V | - | V | - | - | - |
|  | 50 | A | V | D | - | I | V | - |
|  | 103 | - | - | - | V | I | - | - |
|  | 104 | - | V | D | - | V | - | - |
|  | 53 | - | - | - | V | - | - | - |
|  | 55 | V | V | D | - | V | - | - |
|  | 57 | V | V | - | V | - | - | - |
|  | 108 | V | V | - | V | - | - | - |
|  | 58 | - | V | - | V | - | - | - |
|  | 59 | - | - | - | V | - | - | - |
|  | 60 | - | V | I | - | - | D | - |
|  | 61 | A | V | D | - | - | - | - |
|  | 62 | A | V | D | - | - | - | - |
| SMAQ | 29 | D | I | - | I | - | - | - |
|  | 63 | D | V | - | I | - | - | - |
| MEDS | 114 | V | V | - | V | V | - | - |
| MNPS | 115 | V | V | - | V | - | - | - |
| DMAS-7 | 31 | A | D | - | V | V | - | - |
|  | 36 | - | V | - | V | V | - | - |
| ARMS-12 | 116 | - | - | - | - | V | - | - |
|  | 44 | A | V | - | I | V | - | - |
|  | 123 | A | V | I | I | V | - | - |
|  | 106 | A | V | - | I | - | - | - |
|  | 64 | - | - | - | - | V | - | - |
|  | 132 | V | V | D | - | - | - | - |
| MGT | 69 | - | V | D | V | V | - | - |
|  | 70 | - | - | - | - | - | V | - |
|  | 118 | - | - | - | V | V | - | - |
|  | 78 | - | - | - | V | - | - | - |
|  | 81 | D | V | - | V | - | - | - |
|  | 83 | - | V | - | - | - | - | - |
|  | 85 | - | - | - | V | - | - | - |
|  | 122 | - | - | - | V | V | - | - |
|  | 89 | - | V | - | V | V | - | - |
|  | 95 | - | - | - | V | - | - | - |
|  | 96 | - | - | - | V | - | - | - |
|  | 102 | - | - | - | V | V | - | - |
|  | 51 | A | V | - | I | - | - | - |
|  | 52 | V | V | I | - | - | - | - |
|  | 53 | - | - | - | V | - | - | - |
|  | 125 | A | V | - | - | - | - | - |
|  | 54 | - | V | D | V | V | - | - |
|  | 56 | - | - | - | V | - | - | - |
|  | 107 | - | - | - | V | - | - | - |
|  | 110 | - | V | - | V | - | - | - |
| MTA - OA | 32 | - | V | - | - | I | - | - |
| MTA - Insulin | 32 | - | V | - | - | I | - | - |
| LMAS-14 | 71 | A | V | - | I | D | - | - |
|  | 111 | A | V | - | - | - | - | - |
| MTA | 72 | V | - | - | - | - | - | - |
|  | 120 | - | - | - | V | V | - | - |
|  | 42 | - | V | - | V | - | - | - |
|  | 105 | - | V | - | - | V | - | - |
|  | 126 | - | V | - | V | - | - | - |
| MARS-5 | 117 | D | V | I | I | V | - | - |
|  | 77 | - | V | D | - | V | - | - |
|  | 92 | V | V | - | V | - | - | - |
|  | 102 | - | - | - | V | V | - | - |
|  | 133 | V | V | - | - | - | - | - |
| A-14 | 74 | - | D | - | - | - | V | - |
|  | 131 | - | D | - | - | V | - | - |
| ARMS-10 | 75 | A | I | D | - | - | - | - |
| MALMAS | 33 | - | V | D | - | I | - | - |
|  | 34 | - | V | D | V | V | - | - |
|  | 45 | - | V | I | V | V | - | - |
| ARMS-D | 37 | A | V | - | V | V | - | - |
| IADMAS | 38 | - | V | D | V | V | - | - |
| GMAS | 119 | V | V | D | V | V | - | - |
|  | 128 | V | V | D | I | - | D | - |
|  | 129 | V | V | D | I | V | - | - |
|  | 134 | V | V | - | V | V | A | - |
|  | 65 | V | I | - | - | - | D | - |
|  | 135 | V | - | - | - | - | - | - |
|  | 136 | - | V | D | - | V | - | - |
| MAQ | 97 | - | V | I | - | - | - | - |
| MMAS-5 | 98 | - | - | - | - | - | - | V |
| ProMAS | 49 | - | - | - | - | V | - | - |
|  | 130 | V | V | - | - | V | - | - |
| ARMS-7 | 127 | V | V | I | - | - | - | - |
| 5-item questionnaire | 109 | - | - | - | V | V | - | - |
| 3-item questionnaire | 137 | - | - | - | V | - | - | - |
| AS | 112 | - | V | - | - | V | - | - |
| 12-item questionnaire | 66 | - | V | - | - | - | - | - |
| Mascard | 113 | A | V | - | D | - | - | - |

Note: A = Adequate; ARMS = Adherence to Refills and Medication Scale; AS = Adherence Scale; D = Doubtful; DMAS-7 = 7-item Diabetes Medication Adherence Scale; GMAS = General Medication Adherence Scale; I = Inadequate; IADMAS = Iraqi Anti-Diabetic Medication Adherence Scale; LMAS-14 = Fourteen-item Lebanese Medication Adherence Scale; MALMAS = Malaysian Medication Adherence Scale; MAQ = Medication Adherence Questionnaire; MARS-5 = 5-item Medication Adherence Report Scale; Mascard = Medication Adherence Scale in Cardiovascular disorders; MEDS = Medication Adherence Estimation and Differentiation Scale; MGT = Morisky-Green test; MMAS-5 = 5-item adapted Morisky Medication Adherence Scale; MMAS-8 = 8-item Morisky Medication Adherence Scale; MNPS = Medication Non-persistence Scale; MTA = Measurement of Treatment Adherence; MTA-Insulin = Measurement of Treatment Adherence - Insulin; MTA-OA = MTA-Oral Antidiabetics; PROM = Patient-reported outcome measures; ProMAS = Probabilistic Medication Adherence Scale; SMAQ = Simplified Medication Adherence Questionnaire; V = Very good.
